# Fine taxonomic sampling of nervous systems within Naididae (Annelida: Clitellata) reveals evolutionary lability and revised homologies of annelid neural components

**DOI:** 10.1186/s12983-015-0100-6

**Published:** 2015-04-18

**Authors:** Eduardo E Zattara, Alexandra E Bely

**Affiliations:** Department of Biology, University of Maryland, College Park, MD 20740 USA; Current address: Department of Biology, Indiana University, 915 E. Third Street, Myers Hall 150, Bloomington, IN 47405-7107 USA

**Keywords:** Ancestral character estimation, Annelida, Clitellata, Comparative morphology, Evolution, Homology, Naididae, Nervous systems, Neurophylogeny

## Abstract

**Introduction:**

An important goal for understanding how animals have evolved is to reconstruct the ancestral features and evolution of the nervous system. Many inferences about nervous system evolution are weak because of sparse taxonomic sampling and deep phylogenetic distances among species compared. Increasing sampling within clades can strengthen inferences by revealing which features are conserved and which are variable within them. Among the Annelida, the segmented worms, the Clitellata are typically considered as having a largely conserved neural architecture, though this view is based on limited sampling.

**Results:**

To gain better understanding of nervous system evolution within Clitellata, we used immunohistochemistry and confocal laser scanning microscopy to describe the nervous system architecture of 12 species of the basally branching family Naididae. Although we found considerable similarity in the nervous system architecture of naidids and that of other clitellate groups, our study identified a number of features that are variable within this family, including some that are variable even among relatively closely related species. Variable features include the position of the brain, the number of ciliary sense organs, the presence of septate ventral nerve cord ganglia, the distribution of serotonergic cells in the brain and ventral ganglia, and the number of peripheral segmental nerves.

**Conclusions:**

Our analysis of patterns of serotonin immunoreactive perikarya in the central nervous system indicates that segmental units are not structurally homogeneous, and preliminary homology assessments suggest that whole sets of serotonin immunoreactive cells have been gained and lost across the Clitellata. We also found that the relative position of neuroectodermal and mesodermal segmental components is surprisingly evolutionarily labile; in turn, this revealed that scoring segmental nerves by their position relative to segmental ganglia rather than to segmental septa clarifies their homologies across Annelida. We conclude that fine taxonomic sampling in comparative studies aimed at elucidating the evolution of morphological diversity is fundamental for proper assessment of trait variability.

**Electronic supplementary material:**

The online version of this article (doi:10.1186/s12983-015-0100-6) contains supplementary material, which is available to authorized users.

## Introduction

Complex nervous systems are characteristic of eumetazoan taxa and, because their study can help to understand organismal function and evolution, they have been of particular interest to zoologists for several centuries [1-4]. Nervous systems play crucial roles integrating internal and external information into physiological and behavioral responses [2]. While incredibly diverse across major animal groups, nervous system architectures tend to be, by comparison, relatively well conserved within phyla [1,2]. As a result, many studies aimed at understanding the evolution of animal nervous systems have drawn conclusions from comparisons of only a few representatives from widely distant groups (e.g., flies and mice) [3-6]. Inferences from such studies are typically based on the similarities identified across these distantly related species, but these inferences hinge on the assumption that the traits in question are invariable at lower taxonomic levels. In order to make strong inferences about the evolution of animal nervous systems, their structure needs to be investigated in a broad array of taxa and with fine taxonomic sampling.

**Figure 1:**
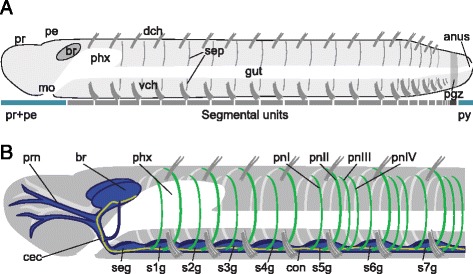
Overview of the naidid ground plan. **A)** Basic annelid body plan. The annelid body consists of an anterior non-segmental region composed of the prostomium (pr) and peristomium (pe), followed by a variable number of segments (grey bars), and a posterior non-segmental region, the pygidium (py). In front of the pygidium is the posterior growth zone (pgz), where new segments are made. **B)** Generalized structure of the nervous system in naidids. This schematic shows the anterior central nervous system (blue), ventral nerve cord neuropil (yellow) and peripheral nervous system (green). Anterior is to the left in this and all figures unless otherwise indicated. Labels: br: brain; cec: circumesophageal connective; con: interganglion connective; dch: dorsal chaetae; gut: ciliated gut; mo: mouth; pe: peristomium; pgz: posterior growth zone; phx: pharynx; pnI-IV: peripheral segmental nerve I-IV; pr: prostomium; prn: prostomial nerves; py: pygidium; s*X*g: segment *x* ganglion; seg: subesophageal ganglion; sep: intersegmental septum; vch: ventral chaetae.

The nervous system of the phylum Annelida (segmented worms) comprises a central nervous system (CNS), composed of an anterior dorsal brain linked via circumesophageal connectives to a ventral nerve cord that is segmentally ganglionated, and a peripheral nervous system (PNS) composed of nerves branching off of the CNS components (Figure 1). Based on descriptions from a limited number of primarily polychaete species (summarized by Bullock and Horridge [2]), the annelid nervous system was originally inferred to have a highly conserved ground plan. However, more recent studies on a broader range of annelids have revealed enormous variation of the annelid nervous system, especially regarding the morphology of the ventral nerve cord and the number and pattern of peripheral nerves, raising new questions about the ancestral architecture and evolution of the annelid nervous system [7].

The Clitellata are a large annelid subclade to which most freshwater and terrestrial annelids belong. The nervous system of clitellates has often been considered to be a simpler and less variable version of the nervous system typical of the primarily marine polychaetes; however, this inference is based on studies of a few clitellate species, mostly earthworms and leeches, with rather specialized morphology [8-10] and which may not closely reflect the ancestral clitellate condition. Clitellates comprise Naididae (water nymph worms), Crassiclitellata (earthworms), Enchytraeidae (pot worms), Lumbriculidae (blackworms) and Hirudina (leeches). The Naididae (*sensu* Erséus *et al.* [11]) are the sister clade to most other clitellates [9,11,12] and knowledge of naidid nervous system architecture is thus of particular importance for inferring how the nervous system has evolved within the clitellates, what the ancestral clitellate nervous system was like, and how it relates to the nervous system of closely related polychaetes.

Available studies of nervous system structure in naidids are few and are difficult to analyze comparatively. Older descriptions based on direct observation, light microscopy, and histological sectioning [13-15] provide different kinds of information than newer studies using immunohistochemistry and whole-mount confocal laser scanning microscopy [16-19]. Studies using consistent techniques, sampling at a fine taxonomic scale, and analyzing data in a phylogenetic framework are needed in order to reconstruct the ancestral naidid nervous system architecture and how it has evolved. Such studies can identify conservative and variable elements of the nervous system and should be particularly useful in identifying possible homologies between neural elements (e.g., nerves, cell types) across species, a task usually made challenging by the high degree of serial duplication characteristic of nervous system evolution.

In this paper, we describe and compare the nervous system architecture of 12 species of Naididae Ehrenberg, 1828 (*sensu* Erseus *et al.* [11]), representing four out of seven naidid subfamilies: Tubificinae - *Tubifex tubifex*; Pristininae - *Pristina leidyi* and *Pristina æquiseta*; Rhyacodrilinae - *Monopylephorus rubroniveus;* and Naidinae - *Dero digitata, Dero furcata, Allonais paraguayensis, Paranais litoralis, Amphichaeta* sp., *Chaetogaster diaphanus, Nais stolci* and *Stylaria lacustris*. We base our descriptions on adult individuals immunostained for acetylated-alpha-tubulin and serotonin, known to label a significant fraction of the neurites and some perikarya [16-21], along with labeled phalloidin to visualize muscular F-actin and DAPI as a nuclear counterstain, and imaged using confocal laser scanning microscopy. We focus in particular on the location and organization of immunoreactive elements of the brain and ventral nerve cord, the topological relationship between the ventral ganglia and the mesodermal septa, and the number and branching architecture of peripheral nerves. Based on our new descriptions and available published data, we identify conserved and variable elements of the naidid nervous system and propose possible homologies for some of these elements. We discuss our findings in the context of current knowledge about the phylogenetic relationships within this family, as well as relationships within the Clitellata and Annelida more broadly, providing insight into the evolution of the nervous system within these groups.

## Results and discussion

An important goal for understanding how animals have evolved is to reconstruct the ancestral features and evolution of the nervous system. Many inferences about nervous system evolution are weak, though, because taxonomic sampling is sparse and phylogenetic distances between species compared are deep. Increasing sampling within specific clades can strengthen such inferences by revealing which features are conserved and which are variable within these groups. In the Annelida, the segmented worms, considerable variation in nervous system architecture has been reported for marine polychaete families [7] but the terrestrial and freshwater Clitellata are typically viewed as having a simple and conserved nervous system [7,15]. However, this view is based on information from a limited number of species spanning this clade and, importantly, no detailed comparative studies within subgroups, such as within families, are available to provide insight into variability and conservation of neural architecture.

To address this gap, we characterized the morphology of the nervous system in 12 species of naidids using immunohistochemistry and confocal laser scanning microscopy. In the interest of brevity, we provide detailed descriptions and diagrams as Supplementary Information, including diagrams of the nervous system of 10 species (Additional file 1: Figure S1, Additional file 2: Figure S2, Additional file 3: Figure S3, Additional file 4: Figure S4, Additional file 5: Figure S5, Additional file 6: Figure S6, Additional file 7: Figure S7, Additional file 8: Figure S8, Additional file 9: Figure S9 and Additional file 10: Figure S10), an overview of a generalized naidid body segment (Additional file 11: Figure S11), image panels showing data for all species (Additional file 12: Figure S12, Additional file 13: Figure S13, Additional file 14: Figure S14, Additional file 15: Figure S15, Additional file 16: Figure S16, Additional file 17: Figure S17 and Additional file 18: Figure S18), and morphological descriptions for each species (Additional file 19). In our descriptions, we use whenever possible the terminology defined by Richter *et al.* [22]. A summary of the character states for all neural traits we found to be variable is provided in Table 1.

**Table 1:** **Character state of variable traits in the naidid nervous sytem**

	**Species**	***Tubifex tubifex***	***Pristina aequiseta***	***Pristina leidyi***	***Monopyleporus rubroniveus***	***Dero digitata***	***Dero furcata***	***Allonais paraguayensis***	***Paranais litoralis***	***Amphichaeta sp.***	***Chaetogaster diaphanus***	***Nais stolci***	***Stylaria lacustris***
	**Subfamily**	**T**	**P**	**P**	**R**	**N**	**N**	**N**	**N**	**N**	**N**	**N**	**N**
Traits													
Anterior nervous system	brain, anterior edge	pr	pr	pr	s1	pe	pr/pe	pe	s1	pr	s1	pr/pe	pr/pe
	brain, posterior edge	pe/s1	pe/s1	pe/s1	s2	s1	s1	s1	s2	pe	s1	s1	pe/s1
	#brain SIR cells	2/6	2	2	2	4	4/6	2	10	2	0	8	4
	#ciliary sense organs	1	2	2	0*	2	2	2	2	2	4/6	2	2
	position ciliary sense organs	br	br	br	NA*	br	br	br	pr	pr	br/pr	br	br
	prostomium shape	cone	prob.	prob.	blunt	cone	cone	cone	blunt	cone	lips	cone	prob.
	eyes	no	no	no	no	no	no	no	no	no	no	yes	yes
Ventral nerve cord ganglia	#parachaetal	0-3	0-2	1-2	1-4	1-4	3-4	2-6	1	2	1-2	1-2	1-2
	#axillar	0-2	1	1	1(2)	1	1	1-2	1-2	1	1	1	1
	#central	0-1	0-2	1-3	1-2	1-3	2	1-2	0-2	0	1-2	1-2	1-2
	#rear	0-many	0-1	0-1	1-2	1	1	1	1	1	1	1	1
	#segments with ant. SIR pattern	3	4	4	4	4	4	4	4	4	3	4	4
	#medullary ant. segments	4	4	4	?	5	4	5	3	2	2	3 + 2	4
	first septum	2/3	2/3	2/3	3/4	3/4	3/4	3/4	3/4	2/3	3, 4/5	3/4	3/4
	ganglion type	non-sept	sept	sept	non-sept*	non-sept	sept	non-sept	sept	non-sept	non-sept	sept	non-sept
PNS	#seg. nerves	4	4	4	4*	4	4	4	4	4	5	4	4
	#segments with ant. PNS	2	4	4	0	4	4	4	1	2	4	4	2

Summary of main nervous system traits found to be variable across the twelve species of Naididae presented in this study. Character states with an asterisk (*) are based on observations of poor quality images and should not be considered as confirmed. See Main Text and Additional file 19 for explanation of traits. Abbreviations by row: Subfamily - T: Tubificinae; P: Pristininae; R: Rhyacodrilinae; N: Naidinae; brain - pr: prostomium, pe: peristomium, s1: chaetigerous segment 1; s2: chaetigerous segment 2; x/y: boundary between x and y; prostomium shape - prob: proboscis; ganglion type – non-sep: non-septate, sep: septate.

Below, we first synthesize the results of our comparative analysis of the nervous system morphology of the 12 naidid species we studied, giving an overview of the common patterns of nervous system components, followed by remarks on their variability. We then discuss the implications of our findings for understanding the stability or lability of neural traits, and the consequences of finding the appropriate homology criteria for inferring the naidid, clitellate and annelid ancestor.

### Overview of naidid nervous system components

The general body and nervous system morphology of all naidid species examined follows the basic clitellate plan (Figure 1A, B and Additional file 11: Figure S11). The nervous system of naidids has three main components: the anterior brain and associated peripheral nervous system, the ganglionated ventral nerve cord, and the segmental peripheral nerves (Figure 1B). The brain, located dorsal to the mouth, is a paired bilobed structure composed of an outer cell cortex (comprising neuron cell bodies and supporting cells) surrounding an inner neuropil (formed by cell free neurites, or neuronal processes), and is linked to the ventral nerve cord by paired circumesophageal connectives, which also connect to paired sets of prostomial peripheral nerves. The prostomium is usually cone-shaped, but may be blunt (*Monopylephorus, Paranais*), elongated into a proboscis (*Pristina, Stylaria*), or very reduced (*Chaetogaster*). *Stylaria* and *Nais* have a pair of lateral pigment-cup eyes located near the posterior edge of the prostomium; other species we studied are eyeless. The ventral nerve cord runs longitudinally down the length of the animal, between the ventral blood vessel and the ventral body wall (Additional file 11: Figure S11). It is composed of clusters of cell bodies (ganglia) linked by short connectives. There is one ganglion per segment, plus a subesophageal ganglion at the anterior end of the cord (Figure 1B); the cell cortex is trough-shaped and a neuropil runs through the trough (Additional file 11: Figure S11). In each segment, a number of peripheral segmental nerves (variously referred to in the literature as ring, circular, peripheral or segmental nerves) branch off perpendicular to the nerve cord (Figure 1B, Additional file 11: Figure S11A). These nerves, designated nerves I to IV based on the antero-posterior order of their roots along the ganglion, pass through the body wall’s muscle layers and run between the muscle and outer epidermis to the dorsal side of the animal.

The following sections describe and compare the anterior nervous system, ventral nerve cord and peripheral nervous system across our study species; within each section, common patterns and conserved elements are described first, followed by a description of the variable elements.

### Anterior nervous system: brain and prostomial nerves

**Figure 2:**
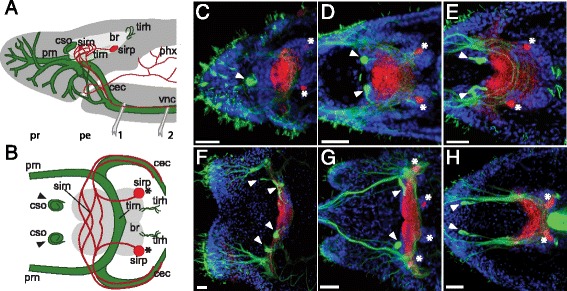
Naidid anterior nervous system. **A**-**B)** General morphology of the anterior nervous system in naidids in lateral **(A)** and dorsal **(B)** views. Brain cell bodies are represented in light grey, acetyl-tubulin immunoreactive (acTIR) structures in green, and serotonin immunoreactive (SIR) structures in red. **C-H)** Diversity in naidid anterior nervous system morphology, as represented by 6 out of the 12 species studied; see also Additional file 12: Figure S12 for data on the full set. Note the variation in the position and number of ciliary sense organs (arrowheads) relative to the SIR brain neuropil, visible as a red mass of SIR neurites; note also the location and number of SIR perikarya (asterisks). Images are intensity sum projection of dorsal Z-stacks of *Tubifex tubifex* **(C)**, *Pristina leidyi* **(D)**, *Dero furcata* **(E)**, *Chaetogaster diaphanus* **(F)**, *Stylaria lacustris* **(G)** and *Paranais litoralis* **(H)**. Specimens were stained for DNA (blue), serotonin (red) and acetyl-tubulin (green). br: brain; cec: circumesophageal connectives; cso: ciliary sense organs (arrowheads); phx: pharynx; prn: prostomial nerves; sirn: serotonin immunoreactive neuropil; sirp: serotonin immunoreactive perikarya (asterisks); tirh: acetyl-tubulin immunoreactive hairs; tirn: acetyl-tubulin immunoreactive neurites, vnc: ventral nerve cord. Scale bars: 25 μm.

**Figure 3:**
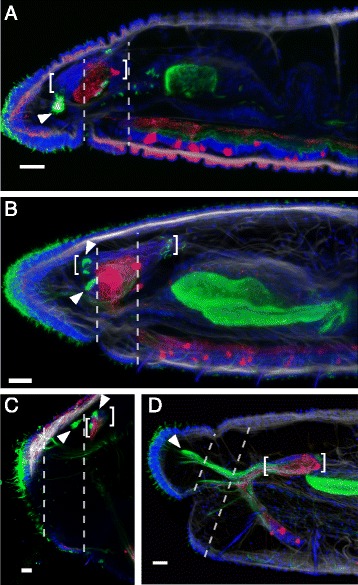
Variation in position of the brain and ciliary sense organs, as represented by 4 naidid species. Images are intensity sum projection of sagittal Z-stacks. Brain boundaries are shown by paired brackets; approximate prostomium/peristomium and peristomium/segment 1 boundaries are marked by dashed lines; ciliary sense organs are indicated by arrowheads. The brain is located almost completely within the prostomium and peristomium in *Tubifex tubifex* **(A)**, peristomium and segment 1 in *Allonais paraguayensis* **(B)**, segment 1 in *Chaetogaster diaphanus* **(C)** and back in segments 1 and 2 in *Paranais litoralis* **(D)**. Specimens were stained for DNA (blue), serotonin (red), acetyl-tubulin (green), and F-actin (white). The dense acetyl-tubulin staining near the center of the animal in A, B, and D corresponds to the heavily ciliated pharynx. Scale bars: 25 μm.

**Figure 4:**
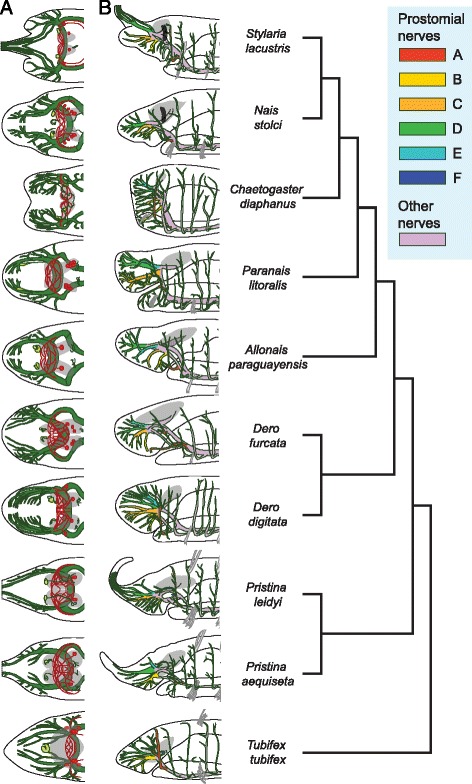
Variation in the architecture of the anterior nervous system across 10 naidid species. **A)** Schematic drawings of the anterior nervous system in dorsal view. Brain lobes are shown in grey, serotonin immunoreactive perikarya and neurites in red, ciliary sense organs in light green, and other acetyl-tubulin immunoreactive nerves in dark green. **B)** Schematic drawings of the prostomial/peristomial nerves in lateral view, color-coded to highlight putative homology assignments. The phylogenetic relationships among the species are shown to the right and are based on recent molecular analyses, as described in the Methods section. Brain is shown in grey; black patches in *Nais stolci* and *Stylaria lacustris* are lateral pigmented eyespots.

The anterior nervous system is composed of common elements connected to each other in a similar manner across all 12 species studied (Figures 2 and 3, Additional file 12: Figure S12 and Additional file 13: Figure S13). The brain is formed by a lobulated cell cortex connected by a dense neuropil, and in most species is located behind the prostomium, spanning the peristomium and segment 1 (exemplified in Figure 3B by *Allonais*). The anterior part of the brain neuropil has a network of serotonin immunoreactive (SIR) neurites, while acetyl-tubulin immunoreactive (acTIR) neurites form an arc across the posterior part (Figure 2A, B). Behind the neuropil, one or more pairs of SIR perikarya are found in the posterior lobe. At the posterior edge of the brain is a set of acTIR hairs that are often associated with muscle fibers attached to that point and to the dorsal body wall. A pair of acTIR ciliary sense organs, evidenced by a coiled neurite structure, are located in front of the brain (Figure 2C-H, Additional file 12: Figure S12, Additional file 13: Figure S13, and Additional file 14: Figure S14). The acTIR tracts in the brain neuropil and the connectives project forward and arborize into a series of prostomial nerves that innervate a dense collection of epidermal acTIR sensory hair cells on the prostomium surface (Figure 4 and Additional file 14: Figure S14). Based on the relative location of nerve roots branching off the circumesophageal connectives, the general patterns of nerve arborization, and the prostomial regions innervated, we tentatively identify six major prostomial nerve branches in the naidids (branches A to F, color coded in Figure 4B). Given the limited number of specimens and range of ages examined here, these homology assignments are necessarily preliminary, but should prove useful as a guide for future studies.

While this general pattern of anterior nervous system elements is shared by most species, we found considerable variation in the location of the brain, number of SIR perikarya, number and location of acTIR ciliary sense organs, and structure and origin of acTIR prostomial nerves across the species examined in this study. With respect to the position of the brain (Figure 3 and Additional file 13: Figure S13), we found that *Tubifex* has a brain that is displaced anteriorly, straddling the prostomium/peristomium (Figure 3A and Additional file 13: Figure S13A); *Chaetogaster*, which has a reduced prostomium, has a relatively small brain located level with the chaetae of segment 1 (Figure 3C and Additional file 13: Figure S13J); and *Monopylephorus* and *Paranais* have a brain that is displaced posteriorly into segments 1 and 2 (Figure 3D, Additional file 13: Figure S13D and S13H). The number and location of SIR perikarya in the brain also varies among species, even between close relatives (Figure 4A; see also Table 1). We detected no SIR perikarya in *Chaetogaster*, one pair in both *Pristina* species, *Monopylephorus, Amphichaeta* and *Allonais*, two pairs in *Stylaria* and *Dero digitata*, three pairs in *Tubifex* and *Dero furcata*, four pairs in *Nais* and five pairs in *Paranais*. Interestingly, we only detected a single pair of SIR perikarya in the brain of a recently hatched *Tubifex* (data not shown), instead of the three pairs scored in older worms, suggesting that the number of SIR perikarya may also vary with developmental stage. All species examined have a pair of acTIR ciliary sense organs located at the anterior edge of the brain with the exception of *Tubifex,* which has a single, medial organ which may represent fusion of the original pair, and *Chaetogaster*, which has two pairs of ciliary sense organs (Figures 2, 3, 4A, Additional file 12: Figure S12, Additional file 13: Figure S13, and Additional file 14: Figure S14). *Paranais, Amphichaeta* and *Chaetogaster* also differ from the rest of the species in that the acTIR ciliary sense organs are located at a short distance from the brain, rather than against the anterior edge, and are connected to the main neuropil by acTIR neurite bundles. Although the number of major prostomial nerve branches is largely similar across species, the location of roots of the prostomial acTIR nerves differs according to differences in the shape of the prostomium, and some nerves are absent (Figure 4 and Additional file 14: Figure S14): for example, the dorsal projecting nerve F is not found in *Nais* and *Stylaria*. In species where the prostomium elongates into a proboscis, namely *Pristina* and *Stylaria* (which likely evolved prostomium elongation independently), nerve D projects forward to innervate this structure (Figure 4B). While nerve A is closest to the eye in *Nais* and *Stylaria*, we could not verify whether these were actually connected due to signal masking by the eye’s pigment.

### Ventral nerve cord

**Figure 5:**
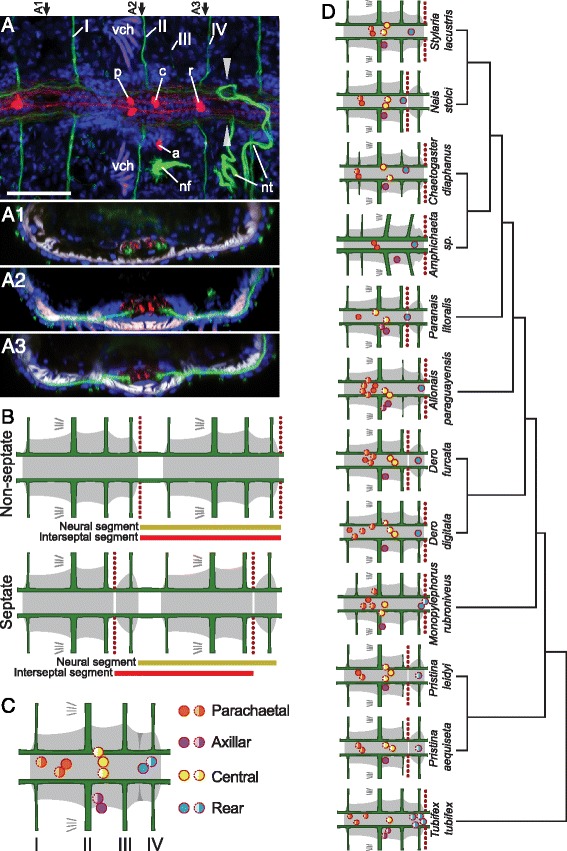
Conservation and variation of the segmental nervous system in naidids. **A)** Structure of a ventral nerve cord ganglion. Image A is an intensity sum projection of a ventral view of a trunk segment from *Allonais paraguayensis*, with transverse reconstructions to show the structure of the connective (A1) and ventral ganglia at two levels (A2, A3). Specimen was stained for DNA (blue), serotonin (red), acetyl-tubulin (green)), and F-actin (white). Segmental nerves are labeled I-IV; serotonin immunoreactive perikarya are within the parachaetal (p), central (c), axillar (a) or rear (r) group; ventral chaetae (vch) are visible due to birefringence. The paired arrowheads mark the position of the mesodermal septum. The looping, acetyl-tubulin positive structures in the lower and right part of the image correspond to a ciliated nephridium (nf: nephridial funnel; nt: nephrotubule). Scale bar: 25 μm. **B)** Diagram of non-septate and septate ganglia. Dashed vertical lines represent the mesodermal septa, and horizontal bars indicate the span of a “neural segment” (defined as an entire ganglion and the interganglionic space anterior to it) and an “interseptal segment” (defined as the region between two consecutive septa). **C)** Generalized pattern of serotonin immunoreactive perikarya in a generic naidid trunk segment. Full circles represent cells that are always or almost always present, while half-circles represent cells whose presence varies among species, individuals and/or segments. **D)** Nervous system structure of a typical trunk segment for each of the 12 species studied. Diagrams show typical pattern of serotonin immunoreactive perikarya of the ganglion (colored according to putative homology group assignments shown in **C)**, location of peripheral nerve roots and location of septa. The phylogenetic relationships among the species are shown to the right and are based on recent molecular analyses, as described in the Methods section.

**Figure 6:**
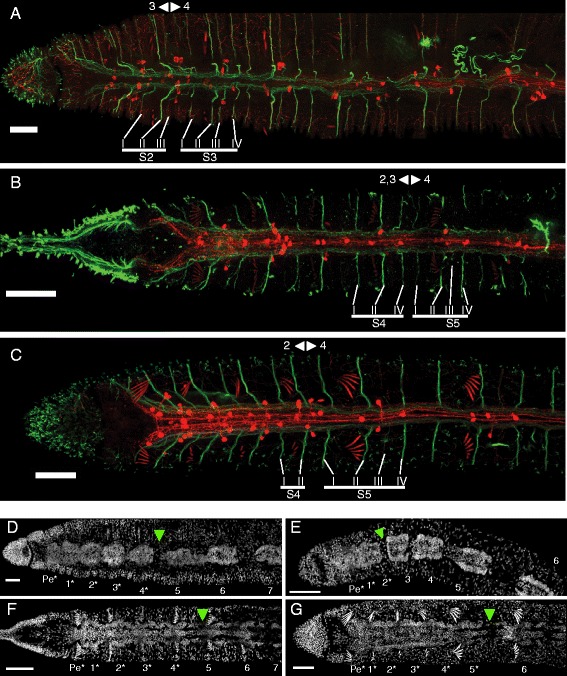
Central and peripheral nervous system of anterior segments of naidids. **A**-**C)** Ventral maximum intensity projections of the anterior end of *Tubifex tubifex* **(A)**, *Pristina aequiseta* **(B)** and *Allonais paraguayensis* **(C)**, showing the ventral nerve cord neuropil and segmental peripheral nerves. Labeled acetyl-tubulin immunoreactive nerves (green) and serotonin immunoreactive nerves and perikarya (red) consistently show a different pattern in anterior-most segments as compared to more posterior segments, but the level at which this transition occurs varies across species, as well as across the elements of the nervous system. The transition between the anterior and posterior pattern of segmental nerves is indicated by the back-to-back arrowheads (number of segmental nerves per segment indicated beside arrowheads). Nerve identity is shown below for segments flanking the boundary. **D**-**G)** Ventral views of DAPI stained specimens of *Tubifex tubifex* **(D)**, *Amphichaeta* sp. **(E)**, *Pristina leidyi* **(F)**, and *Allonais paraguayensis* **(G)**. The green arrowhead marks the location of the anterior-most connective; anterior segments forming a medullary superganglion are labeled by asterisks. Scale bars: 25 μm.

**Figure 7:**
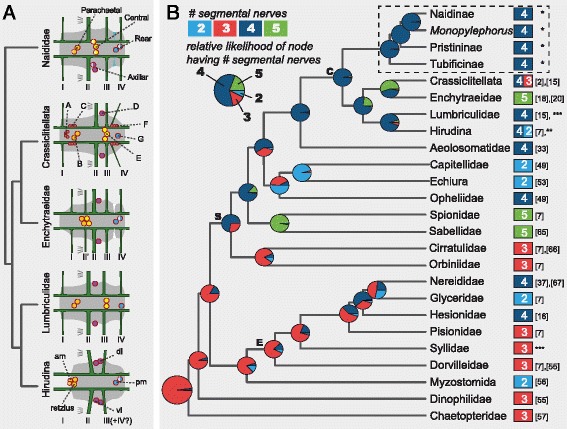
Phylogenetic distribution of serotonin immunoreactive perikarya and peripheral nerve patterns. **A)** Reconstruction of the basal architecture of a trunk ventral ganglion across clitellate groups, based on this study and previous reports [20,27,29,32,35,36]. Colored circles represent serotonin immunoreactive perikarya, with color representing putative homologous groups; A to G cells in Crassiclitellata refer to cell groups defined for *Lumbricus terrestris* [35]; anteromedial (am), dorsolateral (dl), ventrolateral (vl), posteromedial (pm) and Retzius cells in Hirudina refer to cell groups defined for leech species [27,29,32]. Segmental peripheral nerves are shown aligned according to their position along the ganglion. **B)** Phylogenetic mapping of the number of segmental peripheral nerves across Annelida, and maximum likelihood estimations of the ancestral state at each node. Pie charts illustrate the relative likelihood of a given node having had each character state, and were calculated in R [63] using the *ace* function from the *ape* package [64]. Annelid relationships are based on recent phylogenetic studies [50,52]. Labeled ancestral nodes are for Errantia (E) polychaetes, Sedentaria (S) polychaetes and Clitellata (C); the dashed box highlights data from this paper. Nerve number for each group is based on information from one or a few species, except for Naididae, where ancestral condition was estimated from our 12 study species. References are indicated on the right, in brackets for published works; *: data from this study; **R. Hessling, personal communications in [7]; ***E.E. Zattara, unpublished observations.

The ventral nerve cord has a similar architecture in all studied species (Figures 5, 6, 7, Additional file 15: Figure S15, Additional file 16: Figure S16 and Additional file 17: Figure S17). It is formed by a continuous neuropil running through segmentally iterated ganglia, with the neuropil containing acetyl-tubulin immunoreactive (acTIR) and serotonin immunoreactive (SIR) neurites that form longitudinal nerve tracts. The acTIR neurites are found lateroventrally while SIR neurites tend to be medial and dorsal (Figure 5A, A1 and Additional file 15: Figure S15). These nerve tracts are linked by segmentally iterated transverse commissures of variable acTIR neurite density. Phalloidin staining indicates that the ventral cord is sheathed by a thin muscular tunic (Figure 5A1). In all ventral nerve cord ganglia a number of SIR perikarya are found connected to the longitudinal SIR nerve tracts. Based on their location in the ganglion relative to the peripheral nerve roots, we recognize four positional types of SIR perikarya (Figure 5C): 1) parachaetal cells, located within the anterior third of the ganglion, approximately medial to the neuropil and level with the ventral chaetae, between nerve roots I and II; 2) axillar cells, located within the middle third of the ganglion, lateroventrally outside of the neuropil, either to the left or right, and always behind nerve root II; 3) central cells, located more medially within the middle third of the ganglion and closer to the neuropil but level with the axillar cells; and 4) rear cells, located within the posterior third of the ganglion and medial to the neuropil, between nerve roots III and IV. Notably, in all species examined, the anterior-most ganglia have a pattern of SIR perikarya that is clearly different from more posterior segments (Figure 6A-C and Additional file 16: Figure S16). Within a single trunk ganglion, SIR cells are asymmetrically distributed, mostly evidenced by the presence of axillar cells at only one side; however, consecutive ganglia show alternating mirror symmetry with respect to the mid-sagittal plane. In contrast, anterior ganglia have a larger number of SIR perikarya and these are arranged symmetrically with respect to the mid-sagittal plane. Based on counts from 1–4 individuals per species (~30 specimens across all species), the following general patterns emerge: a) on average, there are approximately 80% more SIR perikarya in anterior ganglia than in more posterior ganglia; b) the subesophageal ganglion has fewer SIR perikarya than the ganglia of the anterior segments, but more than more posterior ganglia; c) ganglia of segments 1 and 2 have about the same number of SIR perikarya per segment, and this number is the highest in the body; d) ganglia of segments 3 and 4 have fewer perikarya than segments 1 and 2, but more than more posterior ganglia. In all cases, SIR perikarya of the anterior ganglia are arranged in a bilaterally symmetric pattern resembling what would be obtained by the superposition of perikarya from two consecutive more posterior ganglia. In all species, the ganglia of the peristomium and anterior-most segments adjoin one another, forming a superganglion that adopts a medullary configuration (Figure 6D-G and Additional file 17: Figure S17). These ganglia, especially the first three, are displaced posteriorly relative to other segmental organs, as compared to their position in more posterior segments.

Despite the common architecture of the ventral nerve cord, we found considerable variation among species in the specific location of SIR perikarya in the ganglia, the location along the body where perikarya switch from the above described anterior pattern to the general distribution pattern found in the rest of the segments, and the number of anterior segments forming a medullary superganglion. We also noticed unexpected inter-species variability in the position of the mesodermal segmental septa relative to the ventral nerve cord ganglia. A general pattern of the ganglion SIR cells for each species is shown in Figure 5D; however, these patterns should be considered as approximate, since there is considerable variation from segment to segment even within the same worm. Interpretation is further complicated by the fact that some perikarya show weaker serotonin signal in some segments, and their detection may depend both on their developmental state and the quality of immunostaining. Despite these caveats, we found that the asymmetric trunk pattern of SIR perikarya begins at segment 5 in all species studied (Figure 6B-C and Additional file 16: Figure S16), with the exception of *Tubifex* and *Amphichaeta,* where it starts at segment 4 (Figure 6A, Additional file 16: Figure S16F). The extent of the anterior medullary superganglion is more variable: it includes two ganglia in *Chaetogaster* (Additional file 17: Figure S17E) and *Amphichaeta* (Figure 6E), three ganglia in *Paranais* (Additional file 17: Figure S17D), four ganglia in *Tubifex* (Figure 6D)*, Pristina* (Figure 6F and Additional file 17: Figure S17A)*, Dero furcata* (Additional file 17: Figure S17C)*,* and *Stylaria* (Additional file 17: Figure S17G), and five ganglia in *Dero digitata* (Additional file 17: Figure S17B) and *Allonais* (Figure 6G). In *Nais*, we observed a gap between ganglion 3 and 4, and another between 5 and 6, but not between 4 and 5 (Additional file 17: Figure S17F). *Nais* also was the only species in which we detected SIR perikarya outside of the central nervous system, specifically as segmentally repeated single cells embedded in the body wall, over or close to the lateral line, a subepidermal, lateral cord of cells of unknown function (Additional file 18: Figure S18).

Although a ganglionated ventral nerve cord is present in all species, we found that the position of the ganglia relative to the segmental septa is variable, and specifically takes one of two configurations: non-septate or septate (Figure 5B). Each ganglion is composed of three cell clusters, one anterior, one central and one posterior (Figure 5A). In non-septate ganglia, the intersegmental septum is located behind the posterior cluster, so the whole ganglion is located within the segment; in septate ganglia, the septum is located between the central and the posterior cluster, so the ganglion straddles two segments (Figure 5B). *Tubifex, Monopylephorus, Dero digitata, Allonais, Amphichaeta*, *Chaetogaster* and *Stylaria* have non-septate ganglia, while both *Pristina* species, *Dero furcata, Paranais* and *Nais* have septate ganglia (Figure 5D); note that this trait varies even between phylogenetically close species pairs (e.g. *Stylaria* and *Nais*; *Dero furcata* and *Dero digitata*).

### Peripheral nervous system

The main component of the naidid peripheral nervous system is a system of segmentally iterated acTIR nerves that originate from the neuropil of the ventral nerve cord, exit through the ganglia, pass ventrally through the body wall muscle layer and run subepidermally towards the segment’s dorsum (Figures 1B, 5A and Additional file 11: Figure S11A). Each nerve innervates a number of epidermal sensory hairs. We found that certain nerves can be found in all species examined, while others appear to have been recently gained or lost; we also found that the number of segmental nerves is reduced in the anterior-most segments of most species.

All examined species have four segmental nerves in each trunk segment, except for *Chaetogaster diaphanus* which has five (Figure 5D, Additional file 15: Figure S15). We designated the segmental nerves as nerves I to IV, according to the antero-posterior order in which they branch from the ganglion. The largest nerves are nerve I, located in the anterior half of the segment, and nerve II, which is always located just posterior to the chaetal plane (defined by the ventral and dorsal chaetal bundles) and which sends branches anteriorly to a set of epidermal sensory structures closely associated with the chaetae. Nerves III and IV are usually smaller, and one of the two is generally associated with the intersegmental septum; which nerve this is depends on whether the ganglion is septate or not (Figure 5B). Some species diverge from this general pattern, however (Figure 5D): in *Pristina leidyi* (Additional file 15: Figure S15C), *Pristina aequiseta* (Additional file 15: Figure S15B)*,* and *Allonais paraguayensis* (Figure 5A) nerve III is very small or absent; in *Amphichaeta* sp. (Additional file 15: Figure S15G) and *Chaetogaster diaphanus* (Additional file 15: Figure S15H) there is a small fifth nerve located between nerves I and II (labeled as “2” in arabic numerals in Additional file 15: Figure S15G, H); in *Amphichaeta* sp. we could not detect nerve IV and nerve III is displaced backwards.

We performed an ancestral character estimation analysis to reconstruct the evolution of trunk segment nerve number across annelids, using our data and published information from across the phylum. This analysis indicates that four segmental nerves is the most likely state for the last common ancestor of the naidids (Figure 7B). Our analysis also suggests that the number of segmental nerves has been evolutionarily labile, increasing and decreasing at several points during the evolution of annelids, and that basal stem annelids most likely had three segmental nerves.

The segmental nerve patterns described above are consistent across all segments within a species with the exception of the anterior-most segments, which have fewer nerves the closer they are to the anterior end (Figure 6A-C, Additional file 16: Figure S16). Species vary considerably in the degree of nerve number reduction and in the number of anterior segments that show reduced nerve number. In most species, the full complement of nerves begins in segment 5. However, it begins in segment 3 in *Tubifex*, *Amphichaeta* and *Stylaria*, segment 2 in *Paranais* and in segment 1 (no reduction) in *Monopylephorus.*

### Fine taxonomic sample of Naididae reveals conservation and lability of neural traits

To gain better understanding of nervous system evolution within Clitellata, we described and made a comparative analysis of the nervous system of 12 species of Naididae, a basally branching clitellate family [9,23-25]. Although we found many similarities between nervous system architecture in naidids and that of other clitellate groups [15,20,23], our study identified many features that are variable within this family of clitellates, including some that are variable even among relatively closely related species (Table 1). Variable features of the nervous system include the location of the brain, the number of ciliary sense organs, the presence of non-septate or septate ganglia along the ventral nerve cord, the distribution of serotonergic cells in the brain and ventral ganglia, and the number of peripheral nerves. Below, we discuss interspecific and intra-individual variation in the distribution of serotonin immunoreactive perikarya in the central nervous system and potential homologies between Naididae and other clitellates; we address the unexpected interspecific variability in the position of segmental septa relative to ventral ganglia; and we show how using ganglia rather than body segments to identify peripheral nerves can help reveal nerve homologies across Annelida. We end by highlighting the importance of fine taxonomic sampling in comparative studies aimed at elucidating the evolution of morphological diversity.

### Patterns of serotonin immunoreactive perikarya in the annelid central nervous system indicate that segmental units are not structurally homogeneous

The central nervous system of annelids typically includes serotonin immunoreactive (SIR) cells, which are putatively associated with motor neurons [17,26-31]. The distribution patterns of these cells vary among species and body regions, and have been suggested to be potentially useful taxonomic traits [17]. Our data on 12 species of naidid annelids show that SIR perikarya distribution patterns in the brain and ventral nerve cord can vary considerably across species and even within individuals, both along the antero-posterior body axis and potentially between developmental stages. Despite this variability, the positions of serotonin-positive perikarya in the ventral nerve cord ganglia show consistent enough patterns to suggest putative homologies both within naidids and between naidids and other clitellate groups.

We found that the number of paired serotonin immunoreactive (SIR) perikarya in the brain varies across naidids. While a single pair of SIR perikarya is the most common arrangement for the group, the range is quite broad, from no SIR perikarya in *Chaetogaster* to 5 pairs in *Paranais*. The number of perikarya does not appear to be related in any obvious manner to species attributes such as size, habitat or preferred movement type. While in most species the number of serotonin-positive perikarya is lower in the brain than in the body ganglia, we found that the reverse is true in both *Nais* and *Paranais*. Thus, the number of perikarya in the brain is not always less than that in the ventral ganglia, as had been previously suggested [17]. Our observation that *Tubifex* juveniles had only two serotonin-positive cells in the brain while adult worms have six cells suggests that this number can change post-embryonically; caution is recommended when using the number of serotonin-positive cells in the brain as a diagnostic feature for species identification.

In all but the anterior-most segments, serotonin immunoreactive (SIR) perikarya in ventral ganglia have an asymmetrical distribution, with consecutive segments having patterns that are mirror-images of one another. This alternating pattern has been previously described in three naidid species, all three being naidines [17]; our study extends the distribution of this pattern in naidids and close relatives to seven other naidines, a rhyacodriline, two pristinines, and a tubificine. Such an alternating pattern is also present in leeches [27,29,32], a more distantly related group of clitellates, and even the polychaetous *Aeolosoma* sp. has a single yet alternating serotonergic cell in each ventral ganglion [33]. Thus, the alternating pattern is not an autapomorphy of Naididae, as suggested elsewhere [17], but a feature common to many clitellates and related annelids. The developmental processes that generate this pattern have not been studied in naidids, but during development of the leech species *Helobdella triserialis*, *Theromizon rude* and *Hirudo medicinalis* paired serotonergic precursor cells in consecutive segments make contact with each other and one member of each pair undergoes cell death in a pattern that alternates across segments, setting up a similar alternating pattern of unpaired cells as seen in the naidids [27,29,32]. Whether a similar mechanism is responsible for the SIR perikarya distribution in naidids remains to be determined.

In contrast to trunk ganglia, serotonin immunoreactive (SIR) perikarya in the anterior-most ganglia of naidids are more numerous and show a symmetrical pattern that roughly matches the overlay of two consecutive trunk ganglia. In naidines, anterior segments often develop post-embryonically by paratomic fission [34], and it has been suggested that the symmetrical pattern of SIR perikarya in those segments results from such segments being at an earlier developmental stage in which differential cell death in consecutive segments has not yet taken place [17]. However, our study indicates that anterior segments have a symmetrical pattern not only in fissioning species but in two non-fissioning species as well, *Tubifex* and *Monopylephorus*. Furthermore, in the pristinines, six anterior segments form during fission [19] yet only the four anterior-most segments show symmetrically arranged SIR perikarya, indicating that an asymmetrical pattern can be established during fission as well.

Our finding that naidids have a different distribution of serotonin immunoreactive (SIR) perikarya in anterior segments as compared to more posterior segments is consistent with data from several other annelid groups. In the crassiclitellate earthworm *Lumbricus terrestris*, a subesophageal medullary superganglion formed by two fused anterior ganglia has over three times as many SIR perikarya as trunk ganglia, while segments 4–10 have twice as many [28]. In the leech *Hirudo medicinalis*, eight SIR perikarya per ganglion are found in the six anterior ganglia, with four of these being fused into a subesophageal medullary ganglion and two remaining as free ganglia; in contrast, only seven SIR perikarya per ganglion are found in more posterior cells [26]. In the freshwater polychaete *Aeolosoma* sp., the anterior-most two ganglia have four SIR perikarya, while remaining trunk ganglia have just a single SIR cell [33]. Thus, the presence of two clearly different distribution patterns of SIR perikarya along the body, with a clear break at a specific point along the antero-posterior axis, is widespread among clitellates and is even found outside of this clade. The location of this transition between the two patterns is different for different groups, however. It would be interesting to investigate whether other nervous system components and other organ systems also show a similar antero-posterior break, and whether the presence of such boundaries indicates some degree of “cephalization” of the anterior-most segments.

### Preliminary homology assessments suggest gains and losses of serotonin immunoreactive cell sets across the Clitellata

Based on our studies of naidids, serotonin immunoreactive (SIR) perikarya within the ventral nerve cord ganglia appear to show a common pattern across species. These cells form four spatially segregated sets of cells, which we termed parachaetal, central, axillar and rear cells (Figure 5C). When the naidid pattern is compared with SIR cell patterns reported for representative crassiclitellates (*Lumbricus terrestris* and *Eisenia foetida*) [35,36], enchytraeids (*Enchytraeus crypticus*) [20], hirudines (*Helobdella triserialis*, *Theromizon rude* and *Hirudo medicinalis*) [27,29,32] and lumbriculids (*Lumbriculus variegatus*, E.E.Z. unpublished observations), a very preliminary homology assignment across the clitellates is possible, using as criteria the axial position of the cells along the length of the ganglion and the topological relationship of the cells to the peripheral nerve roots (Figure 7A). By these criteria, the naidid parachaetal cells might be homologous to *Lumbricus*’ B cells, the leech’s anteromedial or Retzius cells, and unnamed anterior cells found in *Enchytraeus* and *Lumbriculus*; the central cells might be homologous to E cells in *Lumbricus* and to unnamed midline cells in *Enchytraeus* and *Lumbriculus*, while being absent in hirudines; the axillar cells might be homologous to D cells in *Lumbricus*, ventro and dorsolateral cells in hirudines, and unnamed lateral cells in *Enchytraeus* and *Lumbriculus*; and the rear cells might be homologous to G cells in *Lumbricus*, posteromedial cells in hirudines, and unnamed midline posterior cells in *Enchytraeus* and *Lumbriculus*. Interestingly, *Lumbricus terrestris* seems to have a number of SIR cell sets clearly absent in more distantly related groups (namely, A, C and F cells). The total number of cells per trunk ganglion varies significantly among groups: naidids reported here have 4–11 cells, leeches [26,27,29], enchytraeids [20] and lumbriculids (E.E.Z. unpublished observations) have 7–9 cells, while earthworms have 30–80 cells [35,36]. The homology assignments we propose here are necessarily tentative, and future studies including developmental and neuronal connectivity studies are needed to confirm them; nonetheless, even this preliminary comparison across clitellates highlights that whole sets of serotonin immunoreactive cells have been gained or lost throughout the evolution of this group of annelids.

### The relative position of neuroectodermal and mesodermal segmental components is evolutionarily labile

An unexpected finding from our study is that the position of the segmental ganglia relative to the septa (which are used to define morphological segment boundaries) varies across species, and even among close relatives. We found that some naidids have non-septate ganglia (in which the entire ganglion falls between consecutive septa) while others have septate ganglia (in which the ganglion straddles two consecutive segments, with a septum falling across the ganglion). Among our study species, we found examples of ganglion type being variable among closely related genera (*Nais* and *Stylaria*), and even within a single genus (*Dero digitata* and *Dero furcata*). Among other clitellates, ganglion type is also variable. Currently available descriptions indicate that *Lumbricus* and *Eisenia* (both in Crassiclitellata) as well as *Lumbriculus* (Lumbriculidae) have non-septate ganglia [15,23], while *Enchytraeus* (Enchytraeidae) has septate ganglia [20]; leeches cannot be categorized since they have no septa at all [23]. Among polychaetes, septate ganglia have been described in the nereids *Neanthes*, *Platynereis* and *Hediste* [37] and this ganglion type is not uncommon in other polychaete groups as well [7]. Although variability in ganglion type can be inferred from the literature, sampling density remains sparse outside of the naidids and none of these previously published observations have been made within a comparative framework; to our knowledge, this is the first report showing that ganglion type can vary even between closely related species. Given our observations, we conclude that the relative position of neuroectodermal elements (i.e., neural ganglia) and mesodermal elements (i.e., septa) of each segment can experience frequent shifts over evolutionary time, and these elements should not be assumed to be in the same register within a group.

We hypothesize that the evolutionary lability of the relative position of neural ganglia and mesodermal septa in clitellates likely reflects the considerable developmental independence of ectodermal and mesodermal teloblastic bandlets during embryogenesis [38,39]. As indicated by work done in *Tubifex hattai* [38-40] and several leech species [41-44], in clitellates much of a segment’s ectodermal and mesodermal tissues arises through teloblastic growth, in which a small set of large stem cells (four ectodermal and one mesodermal teloblast pairs) divide asymmetrically leaving behind bandlets of founder cells that will each form components of one or two consecutive segments. Ablation experiments in *Tubifex* suggest that ectodermal segmentation comprises an initial autonomous morphogenetic stage, followed by mesoderm-dependent alignment of segmental elements [39]. Mesodermal segmentation, on the other hand, does not require segmented ectoderm [38]. Similar results have been reported for leeches [45-47]. Given this degree of independence in the development of these two tissue layers, evolutionary changes in ectoderm/mesoderm alignment may arise relatively easily within clitellates.

Interestingly, even though the relative position of septa and ganglia varies across species, we found that all septate ganglia are septate in a similar manner, with the septum consistently located at two-thirds of the ganglion length. This uniformity in the configuration of septate ganglia suggests that there may be developmental constraints restricting the possible locations of the septa relative to the ganglia. Furthermore, the septate/non-septate condition appears to be fixed within a species, suggesting a strong genetic control. The developmental mechanisms that keep ectoderm and mesoderm in the same register intraspecifically while allowing for interspecific shifts in Naididae are unknown and warrant further investigation.

### The homologies of segmental nerves are clarified by scoring their position relative to segmental ganglia rather than to segmental septa

Among our study species, the most common configuration of the peripheral nervous system was four segmental nerves per trunk ganglion. We did find variation in this pattern, however, with *Chaetogaster* having a fifth small nerve located between nerves I and II, *Pristina* spp. and *Allonais* having nerve III very reduced, and *Amphichaeta* having no detectable nerve IV but a posteriorly displaced nerve III.

Given the variation in ganglion septation we found in our study, we determined that the common approach of naming nerves based on their position within the segment [19,20,37], that is, relative to segmental septa, might not reflect underlying nerve homologies. Instead, we based our naming scheme for segmental nerves on the order of nerve roots along the segmental ganglion (rather than position within the segment), such that nerve I is the anterior-most nerve emanating from the ganglion, nerve II is the next most anterior nerve, etc. In addition to the order of nerve roots along the ganglion, we also considered in our naming scheme the relative sizes and innervation patterns of these nerves, to account for the possibility that certain nerves may be gained or lost over evolutionary time. Using this approach, the arrangement, relative size and innervated structures of nerve roots along the ganglion are almost identical in *Dero furcata* and *Dero digitata* (which, respectively, have septate and non-septate ganglia), whereas a septum-based naming scheme would entail nerves with quite different sizes and innervation patterns being assigned the same nerve number, and nerves associated with the same structures being assigned different nerve numbers (e.g., the nerve innervating the chaetae would be the third nerve in *D. furcata* and the second nerve in *D. digitata*). The homologies of segmental nerves across species that are implied by our naming scheme follow from our expectation that relative slippage of the mesodermal/ectodermal boundaries (see above) is more likely than concerted change in size and innervation patterns of all segmental nerves; thus, naming nerves based on their position along the ganglion rather than relative to mesodermal septa is more likely to reflect nerve homologies. We would discourage others from using septum boundaries to identify segmental nerves in annelids, and encourage the use of ganglion boundaries instead; such an approach should facilitate efforts to trace how segmental nerves evolved in clitellates and other annelids.

Based on our data and ancestral character estimation (*ace)* analysis, four peripheral nerves per trunk ganglion is the most likely ancestral state for Naididae. Outside of the naidids but still among the clitellates, the number of nerves described is four for *Lumbriculus* [48], five for *Enchytraeus* [7,18,20], three for *Lumbricus* [23], and two for adult leeches [23,26,27] (although four nerves are described in embryos of *Erpobdella octoculata*; R. Hessling, unpublished observations in [7]). Under the assumption that these species are representative of their respective groups, our ancestral character estimation analysis supports four peripheral nerves as the ancestral state for Clitellata as a whole (Figure 7B). Out of the four nerves, nerve II is larger and associates with the chaetae in members of Naididae, in *Lumbriculus variegatus* [48] and in *Lumbricus terrestris* [15]. Nerve II is also associated with chaetae in *Enchytraeus crypticus* [20], but it is smaller than the following nerve (Figure 7A). Since five rather than four nerves are present in this species, we speculate that enchytraeids must have either evolved a novel nerve (II’ in Figure 7A), intercalated between nerves I and II, or duplicated nerve II (II’ and II in Figure 7A).

How does this basal clitellate state for segmental nerve number relate to the rest of Annelida? Within polychaetous annelids, the number of nerves reported ranges from none (*Trilobodrilus hermaphroditus*) to eight or more (*Protodrilus* sp.) [7,49]. Putative nerve homologies among taxa have been difficult to establish, in part due to a poor understanding of deep phylogenetic relationships among annelid taxa. However, recent progress in understanding annelid phylogeny [50-52] now provides a stronger framework for mapping of variation in segmental nerves across the phylum, including for ancestral character estimation to reconstruct the most likely number of nerves at the main nodes of the annelid tree, as we have done here (Figure 7B) [7,18,20,23,37,48,49,53-57]. Our analysis supports a previous claim that three segmental nerves represents the ancestral condition for the phylum [2,7]. Interestingly, the analysis suggests that a fourth nerve evolved either at the base of the Sedentaria, the clade of mostly less motile worms within which the clitellates are nested, or shortly thereafter, after the cirratulids + orbinids lineage branched off. According to this reconstruction, the two-nerve state of capitellids and echiurids and the five-nerve state of sabellids and spionids would both be derived from a four-nerve condition. Even if our specific results regarding ancestral character estimation for nerve number at each node are later revised, it is clear that segmental nerves unquestionably have been gained and lost several times during annelid evolution. Finer taxonomic sampling would allow a more precise mapping of novel origins and losses of segmental nerves, which in turn could reveal useful groups in which to study the evolution of neuronal elements within annelids.

## Conclusions

Our comparative description of the nervous system of several species of Naididae and the resulting identification of common patterns and differences in nervous system architecture in this group highlight the potential insights that can be gleaned from comparative morphological studies made at a fine taxonomic scale. Such studies have the power to confirm the deep conservation of certain widespread traits, whose evolution may be strongly constrained by functional or developmental constraints, but also to reveal highly labile traits that can change readily during evolution. Furthermore, such fine-scale comparative studies can contradict prior inferences about which characters are unlikely to vary, as we have shown here, for example, for the septate/non-septate ganglion condition. Obtaining strong data for the conservation or lability of characters is particularly important since traits thought to be well conserved tend to be used as landmarks for homology assignments; thus we recommend ensuring that such conservation has been evaluated at a relatively fine taxonomic scale before using such traits more broadly as a basis for determining homologies.

## Methods

### Animal samples

Specimens of *Tubifex tubifex*, *Pristina leidyi*, *Pristina aequiseta*, *Dero digitata*, *Dero furcata*, *Allonais paraguayensis*, *Paranais litoralis*, *Chaetogaster diaphanus*, *Nais stolci* and *Stylaria lacustris* were obtained from established laboratory cultures [58,59] and specimens of *Monopylephorus rubroniveus* and *Amphichaeta* sp. were field collected. Table 2 provides worm sources, culture conditions, and NCBI accession numbers for available COI and 16S sequence barcodes for the strains used. New COI or 16S sequences were obtained for *Paranais litoralis*, *Dero furcata*, *Pristina leidyi* and *Pristina aequiseta*; primers, PCR parameters, and sequencing methods are as described elsewhere [58].

**Table 2:** **Classification, source and culture conditions for study species**

**Subfamily**	**Species**	**Source**	**Culture conditions**	**Acc. number**
Tubificinae Vejdovsky, 1876	*Tubifex tubifex*	Western Fisheries Research Center, USGS, Sand Point, Lake Washington, WA, USA (supplied by C. Rasmussen)	Sand substrate in artificial spring water aerated flasks, 15C. Fed spirulina pellets.	GenBank: AF534866 (COI)
Pristininae Lastočkin, 1921	*Pristina leidyi*	Pond, Terrapin Softball Complex, University of Maryland, College Park, MD	Paper substrate in artificial spring water bowls, room temperature. Fed spirulina powder.	Genbank: KR296707 (16S rRNA)
Pristininae Lastočkin, 1921	*Pristina œquiseta*	Cichlid fish tanks, Biology/Psychology Building, University of Maryland, College Park, MD	Paper substrate in artificial spring water bowls, room temperature. Fed powdered fish food flakes.	Genbank: KR296708 (16S rRNA)
Rhyacodrilinae Hrabě, 1963	*Monopylephorus rubroniveus*	Charleston, SC, USA	N.A.	GenBank: GQ355379 (COI)
Naidinae Ehrenberg, 1828	*Amphichaeta sp.*	Rhode River, Smithsonian Environmental Research Center, Edgewater, MD, USA	N.A.	GenBank: AF534829 (COI)
Naidinae Ehrenberg, 1828	*Chaetogaster diaphanus*	Edwards Lake, University of Maryland, College Park, MD, USA	Paper substrate in artificial spring water bowls, room temperature. Fed chopped *Allonais*.	GenBank: GQ355366 (COI)
Naidinae Ehrenberg, 1828	*Nais stolci*	Paint Branch, University of Maryland, College Park, MD, USA	Paper substrate in artificial spring water bowls, room temperature. Fed spirulina powder.	GenBank: GQ355369 (COI)
Naidinae Ehrenberg, 1828	*Stylaria lacustris*	Paint Branch, University of Maryland, College Park, MD, USA	Paper substrate in artificial spring water bowls, room temperature. Fed spirulina powder.	GenBank: AF534861 (COI)
Naidinae Ehrenberg, 1828	*Paranais litoralis*	Herring Bay, Fairhaven, MD, USA	Mud substrate in artificial brackish water bowls, 15C. Fed mud supplemented with fish food flakes.	Genbank: KP204261 (COI)
Naidinae Ehrenberg, 1828	*Allonais paraguayensis*	Ward’s Natural Science (sold as *Stylaria*). USA.	Paper substrate in artificial spring water bowls, room temperature. Fed rolled oats.	GenBank: AF534828 (COI)
Naidinae Ehrenberg, 1828	*Dero furcata*	Carolina Biological Supply (found inside a *Daphnia magna* flask)	Paper substrate in artificial spring water bowls, room temperature. Fed rolled oats.	Genbank: KP204260 (COI)
Naidinae Ehrenberg, 1828	*Dero digitata*	Edwards Lake, University of Maryland, College Park, MD	Paper substrate in artificial spring water bowls, room temperature. Fed rolled oats.	GenBank: GQ355368 (COI)

Classification, source and culture conditions for the 12 study species (Annelida: Clitellata: Naididae Ehrenberg, 1828, *sensu* Erseus *et al.* [11]). NCBI accession numbers for 16S rRNA (*Pristina* species) or cytochrome oxidase I (all other species) partial sequences are provided as barcoding reference.

### Immunocytochemistry

Samples were relaxed 10 min in cold (4°C) relaxant solution (10 mM MgCl_2_/5 mM NaCl/1 mM KCl/8% ethanol), fixed 30 min in 4% formaldehyde in 0.75x PBS, and rinsed in 1x PBS. Then they were permeabilized with 0.1% Triton-X in PBS (PBTx), blocked 1 h in 10% normal goat serum (NGS) in PBTx, and incubated 15–20 h at 4°C with mouse anti-acetylated-α-tubulin monoclonal antibody (T6793, Sigma, St. Louis, MO, USA) and rabbit anti-serotonin polyclonal antibodies (S5545, Sigma), both diluted 1:100 in blocking solution. Specimens were then washed in PBTx and incubated 15–20 h at 4°C in blocking solution containing Alexa-Fluor-647-conjugated goat anti-mouse IgG(H + L) antibodies (1:200, A21236, Invitrogen, Carlsbad, CA, USA) and Cy3-conjugated goat anti-rabbit IgG antibodies (1:200, 111-166-003, Jackson Immunoresearch,West Grove, PA, USA), 60 nM Alexa-Fluor-488 phalloidin (A12379, Invitrogen), and 10 μg/mL DAPI. After washing with PBTx and PBS, specimens were transferred through a graded glycerol series and mounted in 25 mM *n*-propyl-gallate in 75% glycerol/25% PBS.

### Image acquisition and analysis

Labeled specimens were mounted on glass slides and imaged using a Leica SP5X confocal laser scanning microscope (Leica, Wetzlar, Germany) under 20x or 40x oil immersion lenses. Z-stacks with 0.5-1.0 μm steps were acquired using the Leica LAS AF software. For each species, the anterior region (prostomium and first seven segments) and several mid-body segments were imaged from a total of at least 6 individuals (two each for lateral, ventral and dorsal anterior views, plus mid-body views). Z-stack images from different specimens and views were analyzed using ImageJ [60] (Bethesda, MD, USA) and Zen 2009 LE (Zeiss, Oberkochen, Germany) to infer the characteristic acetyl-tubulin immunoreactive (acTIR) and serotonin immunoreactive (SIR) elements of the nervous system, as well as other morphological landmarks. We used a combination of maximum intensity projections, depth-color-coded projections and 3D volume reconstructions to guide our interpretation. Based on these image data, we generated hand-drawn representative diagrams (lateral and ventro-dorsal views) of the morphology of anterior and mid-body regions of each species. Representative drawings of the nervous system and associated structures for each species were traced and colored using Adobe Illustrator CS3. We used these summary drawings along with the actual Z-stacks to compare the morphology of all twelve species.

### Phylogenetic analyses and ancestral character estimation

Phylogenetic relationships among the 12 study species were established primarily based on previously published studies [10,12,58,61,62]; where prior studies conflict, phylogenetic positions were resolved according to the results of a recent analysis using the largest dataset of naidid sequences yet analyzed (C. Erséus, personal communications). Relationships among clitellate and polychaete groups were also established based on previous studies [50-52]. Ancestral character estimation of peripheral nerve numbers was made using R [63] with the *ace* function from the *ape* package [64]; the trait value for each group was based on results from either this study or from existing reports for representatives of the group [2,7,15,16,20,37,49,55-57,65-67]. We used maximum likelihood estimation with a custom symmetrical transition rate matrix allowing single-step changes in character state.

### Availability of supporting data

The data supporting the results of this article are included within the article and its additional files.

## Additional files

Additional file 1: Figure S1. Nervous system of *Tubifex tubifex* (Clitellata: Naididae: Tubificinae). Drawings based on observations of individuals stained using anti-acetylated-alpha-tubulin and anti-serotonin antibodies, fluorescently labeled phalloidin and DAPI, imaged as Z-stacks under a confocal laser scanning microscope. Acetylated-tubulin immunoreactive (acTIR) neuropil shown in green, serotonin immunoreactive (SIR) neurites and perikarya shown in red, brain shown in dark gray in A and B, ventral nerve cord ganglia shown in dark grey in C and D, and intersegmental septa shown as dashed dark red lines. A) Lateral view of the anterior end showing ventral nerve cord, segmental peripheral nerves, prostomial nerves, circumesophageal connective and brain. SIR structures shown only for brain. B) Dorsal view of the anterior end, showing same structures as A, plus SIR elements in ventral nerve cord and pharyngeal plexus. C) Lateral view of a typical trunk body segment showing localization of peripheral nerve roots relative to ganglia, septa and chaetae; SIR elements not shown. D) Schematic of the structure of the ventral nerve cord showing localization of peripheral nerve roots relative to ganglia, septa and chaetae. Abbreviations: br: brain; cec: circumesophageal connectives; cso: ciliary sense organ; dch: dorsal chaetae (notochaetae); eye: pigment cup eye (not present in all species); gut: digestive tract; mo: mouth; php: pharyngeal plexus; phx: pharynx; prb: proboscis (not present in all species); prn: prostomial nerve; psn: peripheral segmental nerve; sep: mesodermal septum; sirl: serotonin immunoreactive lateral neuron (not present in all species); sirn: serotonin immunoreactive neuropil; sirp: serotonin immunoreactive perikaryon; tirn: acetyl-tubulin immunoreactive neuropil; vch: ventral chaetae (neurochaetae); vg: ventral ganglion; vnc: ventral nerve cord. Additional file 2: Figure S2. Nervous system of *Pristina aequiseta* (Clitellata: Naididae: Pristininae). Drawings based on specimens prepared and labeled as in Additional file 1: Figure S1. A) Lateral view of the anterior end showing ventral nerve cord, segmental peripheral nerves, prostomial nerves, circumesophageal connective and brain. Serotonin immunoreactive (SIR) structures shown only for brain and pharyngeal plexus. B) Dorsal view of the anterior end, showing same structures as A, plus SIR elements in ventral nerve cord and pharyngeal plexus. C) Lateral view of a typical trunk body segment showing localization of peripheral nerve roots relative to ganglia, septa and chaetae; SIR elements not shown. D) Schematic of the structure of the ventral nerve cord showing localization of peripheral nerve roots relative to ganglia, septa and chaetae. Abbreviations as in Additional file 1: Figure S1. Additional file 3: Figure S3. Nervous system of *Pristina leidyi* (Clitellata: Naididae: Pristininae). Drawings based on specimens prepared and labeled as in Additional file 1: Figure S1. A) Lateral view of the anterior end showing ventral nerve cord, segmental peripheral nerves, prostomial nerves, circumesophageal connective and brain. Serotonin immunoreactive (SIR) structures shown only for brain and pharyngeal plexus. B) Dorsal view of the anterior end, showing same structures as A, plus SIR elements in ventral nerve cord and pharyngeal plexus. C) Lateral view of a typical trunk body segment showing localization of peripheral nerve roots relative to ganglia, septa and chaetae; SIR elements not shown. D) Schematic of the structure of the ventral nerve cord showing localization of peripheral nerve roots relative to ganglia, septa and chaetae. Abbreviations as in Additional file 1: Figure S1. Additional file 4: Figure S4. Nervous system of *Dero digitata* (Clitellata: Naididae: Naidinae). Drawings based on specimens prepared and labeled as in Additional file 1: Figure S1. A) Lateral view of the anterior end showing ventral nerve cord, segmental peripheral nerves, prostomial nerves, circumesophageal connective and brain. Serotonin immunoreactive (SIR) structures shown only for brain and pharyngeal plexus. B) Dorsal view of the anterior end, showing same structures as A, plus SIR elements in ventral nerve cord and pharyngeal plexus. C) Lateral view of a typical trunk body segment showing localization of peripheral nerve roots relative to ganglia, septa and chaetae; SIR elements not shown except for lateral subepidermal perikarya. D) Schematic of the structure of the ventral nerve cord showing localization of peripheral nerve roots relative to ganglia, septa and chaetae. Abbreviations as in Additional file 1: Figure S1. Additional file 5: Figure S5. Nervous system of *Dero furcata* (Clitellata: Naididae: Naidinae). Drawings based on specimens prepared and labeled as in Additional file 1: Figure S1. A) Lateral view of the anterior end showing ventral nerve cord, segmental peripheral nerves, prostomial nerves, circumesophageal connective and brain. Serotonin immunoreactive (SIR) structures shown only for brain and pharyngeal plexus. B) Dorsal view of the anterior end, showing same structures as A, plus SIR elements in ventral nerve cord and pharyngeal plexus. C) Lateral view of a typical trunk body segment showing localization of peripheral nerve roots relative to ganglia, septa and chaetae; SIR elements not shown except for lateral subepidermal perikarya. D) Schematic of the structure of the ventral nerve cord showing localization of peripheral nerve roots relative to ganglia, septa and chaetae. Abbreviations as in Additional file 1: Figure S1. Additional file 6: Figure S6. Nervous system of *Allonais paraguayensis* (Clitellata: Naididae: Naidinae). Drawings based on specimens prepared and labeled as in Additional file 1: Figure S1. A) Lateral view of the anterior end showing ventral nerve cord, segmental peripheral nerves, prostomial nerves, circumesophageal connective and brain. Serotonin immunoreactive (SIR) structures shown only for brain and pharyngeal plexus. B) Dorsal view of the anterior end, showing same structures as A, plus SIR elements in ventral nerve cord and pharyngeal plexus. C) Lateral view of a typical trunk body segment showing localization of peripheral nerve roots relative to ganglia, septa and chaetae. D) Schematic of the structure of the ventral nerve cord showing localization of peripheral nerve roots relative to ganglia, septa and chaetae. Abbreviations as in Additional file 1: Figure S1. Additional file 7: Figure S7. Nervous system of *Paranais litoralis* (Clitellata: Naididae: Naidinae). Drawings based on specimens prepared and labeled as in Additional file 1: Figure S1. A) Lateral view of the anterior end showing ventral nerve cord, segmental peripheral nerves, prostomial nerves, circumesophageal connective and brain. Serotonin immunoreactive (SIR) structures shown only for brain and pharyngeal plexus. B) Dorsal view of the anterior end, showing same structures as A, plus SIR elements in ventral nerve cord and pharyngeal plexus. C) Lateral view of a typical trunk body segment showing localization of peripheral nerve roots relative to ganglia, septa and chaetae. D) Schematic of the structure of the ventral nerve cord showing localization of peripheral nerve roots relative to ganglia, septa and chaetae. Abbreviations as in Additional file 1: Figure S1. Additional file 8: Figure S8. Nervous system of *Chaetogaster diaphanus* (Clitellata: Naididae: Naidinae). Drawings based on specimens prepared and labeled as in Additional file 1: Figure S1. A) Lateral view of the anterior end showing ventral nerve cord, segmental peripheral nerves, prostomial nerves, circumesophageal connective and brain. Serotonin immunoreactive (SIR) structures shown only for brain. B) Dorsal view of the anterior end, showing same structures as A, plus SIR elements in ventral nerve cord and pharyngeal plexus. C) Lateral view of a typical trunk body segment showing localization of peripheral nerve roots relative to ganglia, septa and chaetae; SIR elements not shown. D) Schematic of the structure of the ventral nerve cord showing localization of peripheral nerve roots relative to ganglia, septa and chaetae. Abbreviations as in Additional file 1: Figure S1. Additional file 9: Figure S9. Nervous system of *Nais stolci* (Clitellata: Naididae: Naidinae). Drawings based on specimens prepared and labeled as in Additional file 1: Figure S1. A) Lateral view of the anterior end showing ventral nerve cord, segmental peripheral nerves, prostomial nerves, circumesophageal connective and brain. Serotonin immunoreactive (SIR) structures shown only for brain and pharyngeal plexus. B) Dorsal view of the anterior end, showing same structures as A, plus SIR elements in ventral nerve cord and pharyngeal plexus. C) Lateral view of a typical trunk body segment showing localization of peripheral nerve roots relative to ganglia, septa and chaetae; SIR elements not shown except for lateral subepidermal perikarya. D) Schematic of the structure of the ventral nerve cord showing localization of peripheral nerve roots relative to ganglia, septa and chaetae. Abbreviations as in Additional file 1: Figure S1. Additional file 10: Figure S10. Nervous system of *Stylaria lacustris* (Clitellata: Naididae: Naidinae). Drawings based on specimens prepared and labeled as in Additional file 1: Figure S1. A) Lateral view of the anterior end showing ventral nerve cord, segmental peripheral nerves, prostomial nerves, circumesophageal connective and brain. Serotonin immunoreactive (SIR) structures shown only for brain and pharyngeal plexus. B) Dorsal view of the anterior end, showing same structures as A, plus SIR elements in ventral nerve cord and pharyngeal plexus. C) Lateral view of a typical trunk body segment showing localization of peripheral nerve roots relative to ganglia, septa and chaetae. D) Schematic of the structure of the ventral nerve cord showing localization of peripheral nerve roots relative to ganglia, septa and chaetae. Abbreviations as in Additional file 1: Figure S1. Additional file 11: Figure S11. Naidid segment ground plan. A) Stereogram of a generic naidid segmental unit. B) Internal structure of a body segment. Parasagittal maximum intensity projection of a lateral Z-stack of *Stylaria lacustris* stained for DNA (blue - nuclei), F-actin (red - muscle), acetyl-tubulin (green - primarily peripheral nervous system and ciliated structures (e.g., nephridia and gut)) and serotonin (yellow - primarily ventral nerve cord). Labels: bw: body wall; chl: chloragogen cells; cm: circular muscle; dbv: dorsal blood vessel; dbw: dorsal body wall; dch: dorsal chaetae; ep: epidermis; gut: ciliated gut; ll: lateral line; lm: longitudinal muscle; nf: nephridial funnel; nph: nephridium; nt: nephrotubule; pnI-IV: peripheral segmental nerve I-IV; sep: intersegmental septum; vbv: ventral blood vessel; vbw: ventral body wall; vch: ventral chaetae; vg: ventral ganglion; vnc: ventral nerve cord. Additional file 12: Figure S12. Diversity in anterior nervous system morphology of 12 species of Naididae. Note the variation in the position and number of ciliary sense organs (arrowheads) relative to the SIR brain neuropil, visible as a red mass of SIR neurites; note also the location and number of SIR perikarya (asterisks). Images are intensity sum projections of dorsal Z-stacks of *Tubifex tubifex* (A), *Pristina aequiseta* (B), *Pristina leidyi* (C), *Monopylephorus rubroniveus* (D), *Dero digitata* (E)*, Dero furcata* (F), *Allonais paraguayensis* (G)*, Paranais litoralis* (H), *Amphichaeta* sp. (I)*, Chaetogaster diaphanus* (J), *Nais stolci* (K) and *Stylaria lacustris* (L). Specimens were stained for DNA (blue), serotonin (red) and acetyl-tubulin (green), except for *Monopylephorus* (D), where DNA was degraded and failed to stain. Scale bars: 25 μm. Additional file 13: Figure S13. Variation in position of the brain and ciliary sense organs of 12 species of Naididae. Images are intensity sum projection of sagittal Z-stacks. Brain boundaries are shown by paired brackets; approximate prostomium/peristomium and peristomium/segment 1 boundaries are marked by dashed lines; ciliary sense organs are indicated by arrowheads. *Tubifex tubifex* (A), *Pristina aequiseta* (B), *Pristina leidyi* (C), *Monopylephorus rubroniveus* (D), *Dero digitata* (E)*, Dero furcata* (F), *Allonais paraguayensis* (G)*, Paranais litoralis* (H), *Amphichaeta* sp. (I)*, Chaetogaster diaphanus* (J), *Nais stolci* (K) and *Stylaria lacustris* (L). Specimens were stained for DNA (blue), serotonin (red) and acetyl-tubulin (green), except for *Monopylephorus* (D), where DNA was degraded and failed to stain. Scale bars: 25 μm. Additional file 14: Figure S14. Diversity in prostomial innervation in 12 species of Naididae. Images are maximum intensity projections of sagittal Z-stacks, color coded by depth along the stack (relative color reference scale at bottom right). All panels show staining for acetylated-tubulin. Ciliary sense organs are indicated by arrowheads. Images are of *Tubifex tubifex* (A), *Pristina aequiseta* (B), *Pristina leidyi* (C), *Monopylephorus rubroniveus* (D), *Dero digitata* (E)*, Dero furcata* (F), *Allonais paraguayensis* (G)*, Paranais litoralis* (H), *Amphichaeta* sp. (I)*, Chaetogaster diaphanus* (J), *Nais stolci* (K) and *Stylaria lacustris* (L). Scale bars: 25 μm. Additional file 15: Figure S15. Diversity of ventral nerve cord ganglion architecture in 10 species of Naididae. Images are intensity sum projections of ventral Z-stacks. Specimens were stained for DNA (blue), serotonin (red) and acetyl-tubulin (green). Segmental nerves are labeled I-IV; alternative arabic numerals are shown in G and H. Ventral chaetae (ch) are visible due to birefringence. The paired arrowheads mark the position of the mesodermal septum. The looping, acetyl-tubulin positive structures in the image corresponds to ciliated nephridia (nf: nephridial funnel; nt: nephrotubule). *Tubifex tubifex* (A), *Pristina aequiseta* (B), *Pristina leidyi* (C), *Dero digitata* (D)*, Dero furcata* (E), *Paranais litoralis* (F), *Amphichaeta* sp. (G)*, Chaetogaster diaphanus* (H), *Nais stolci* (I) and *Stylaria lacustris* (J). *Monopylephorus* is not included here due to failure of DAPI staining and low quality of acITR detection. Scale bars: 25 μm. Additional file 16: Figure S16. Central and peripheral nervous system of anterior segments of 9 species of Naididae. Images are intensity sum projections of ventral Z-stacks showing the ventral nerve cord neuropil and segmental peripheral nerves. Labeled acetyl-tubulin immunoreactive nerves (green), and serotonin immunoreactive nerves and perikarya (red) consistently show a different pattern in anterior-most segments relative to more posterior segments, but the level at which this transition occurs varies across species, as well as across the elements of the nervous system. The transition between the anterior and posterior pattern of segmental nerves is indicated by occurs the opposed arrowheads (number of segmental nerves per segment indicated beside arrowheads). Nerve identity is shown below for segments flanking the boundary. Images are from *Pristina leidyi* (A), *Monopylephorus rubroniveus* (B), *Dero digitata* (C)*, Dero furcata* (D), *Paranais litoralis* (E), *Amphichaeta* sp. (F)*, Chaetogaster diaphanus* (G), *Nais stolci* (H) and *Stylaria lacustris* (I). Scale bars: 25 μm. Additional file 17: Figure S17. Diversity in architecture of anterior ventral nerve cord ganglia. Images are intensity sum projections of ventral Z-stacks showing cell nuclei. The green arrowhead marks the location of the anterior-most connective. Images are from *Pristina aequiseta* (A), *Dero digitata* (B)*, Dero furcata* (C), *Paranais litoralis* (D), *Chaetogaster diaphanus* (E), *Nais stolci* (F) and *Stylaria lacustris* (G). Scale bars: 25 μm. Additional file 18: Figure S18. Single serotonin-immunoreactive cells in the lateral body wall of *Nais stolci*. A,B) Representative images of two different segments from different individuals; maximum intensity projections of sagittal Z-stacks of representing thick optical sections level to the lateral body wall of mid-trunk segments. Specimens were stained for DNA (blue), serotonin (red) and acetyl-tubulin (green). ll: lateral line; psn: peripheral segmental nerve; sirl: serotonin-immunoreactive cell; vch: ventral chaetae. Scale bars: 25 μm. Additional file 19:
**Supplementary results.**

## Abbreviations

acTIR: acetyl-tubulin immunoreactive
CNS: Central nervous system
PNS: Peripheral nervous system
SIR: Serotonin immunoreactive
