# Supplementary figures and images for: Fine taxonomic sampling of nervous systems within Naididae (Annelida: Clitellata) reveals evolutionary lability and revised homologies of annelid neural components
Source: Front Zool. 2015 Apr 18;12:8. doi: 10.1186/s12983-015-0100-6 (PMC4424535; doi:10.1186/s12983-015-0100-6)

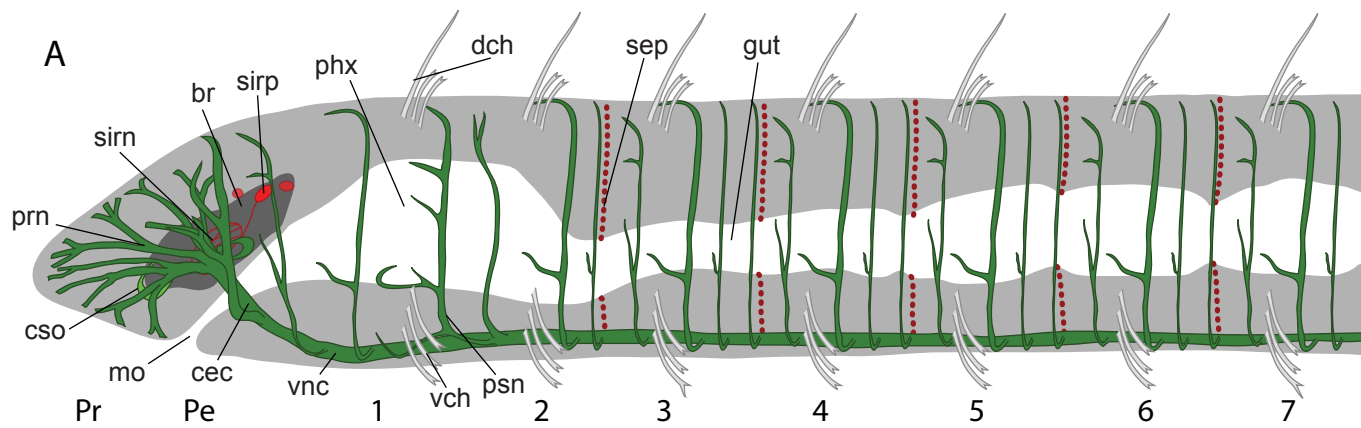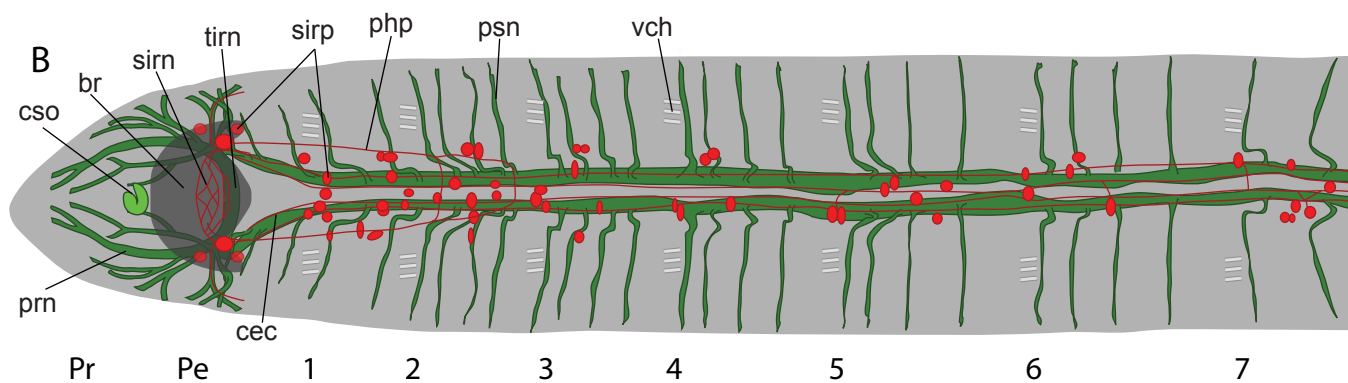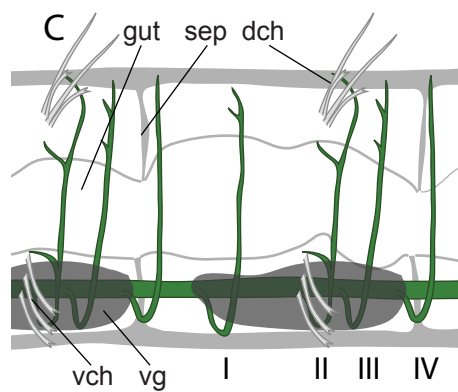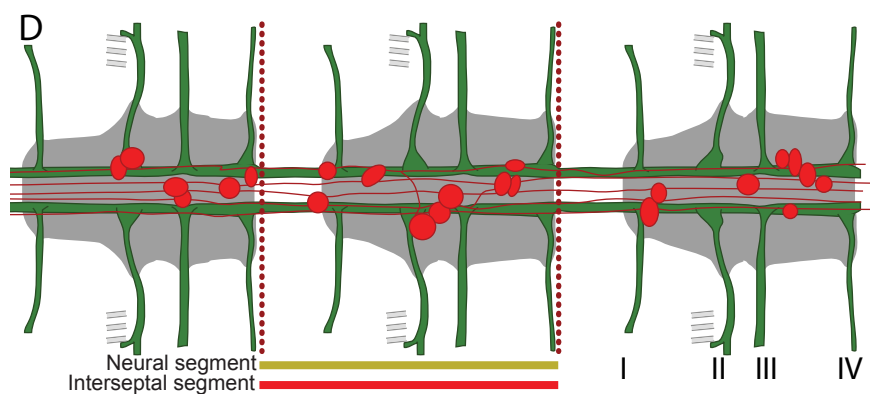

Supplement: Additional file 1: Figure S1. — Nervous system of Tubifex tubifex (Clitellata: Naididae: Tubificinae). Drawings based on observations of individuals stained using anti-acetylated-alpha-tubulin and anti-serotonin antibodies, fluorescently labeled phalloidin and DAPI, imaged as Z-stacks under a confocal laser scanning microscope. Acetylated-tubulin immunoreactive (acTIR) neuropil shown in green, serotonin immunoreactive (SIR) neurites and perikarya shown in red, brain shown in dark gray in A and B, ventral nerve cord ganglia shown in dark grey in C and D, and intersegmental septa shown as dashed dark red lines. A) Lateral view of the anterior end showing ventral nerve cord, segmental peripheral nerves, prostomial nerves, circumesophageal connective and brain. SIR structures shown only for brain. B) Dorsal view of the anterior end, showing same structures as A, plus SIR elements in ventral nerve cord and pharyngeal plexus. C) Lateral view of a typical trunk body segment showing localization of peripheral nerve roots relative to ganglia, septa and chaetae; SIR elements not shown. D) Schematic of the structure of the ventral nerve cord showing localization of peripheral nerve roots relative to ganglia, septa and chaetae. Abbreviations: br: brain; cec: circumesophageal connectives; cso: ciliary sense organ; dch: dorsal chaetae (notochaetae); eye: pigment cup eye (not present in all species); gut: digestive tract; mo: mouth; php: pharyngeal plexus; phx: pharynx; prb: proboscis (not present in all species); prn: prostomial nerve; psn: peripheral segmental nerve; sep: mesodermal septum; sirl: serotonin immunoreactive lateral neuron (not present in all species); sirn: serotonin immunoreactive neuropil; sirp: serotonin immunoreactive perikaryon; tirn: acetyl-tubulin immunoreactive neuropil; vch: ventral chaetae (neurochaetae); vg: ventral ganglion; vnc: ventral nerve cord. [file 12983_2015_100_MOESM1_ESM.pdf]

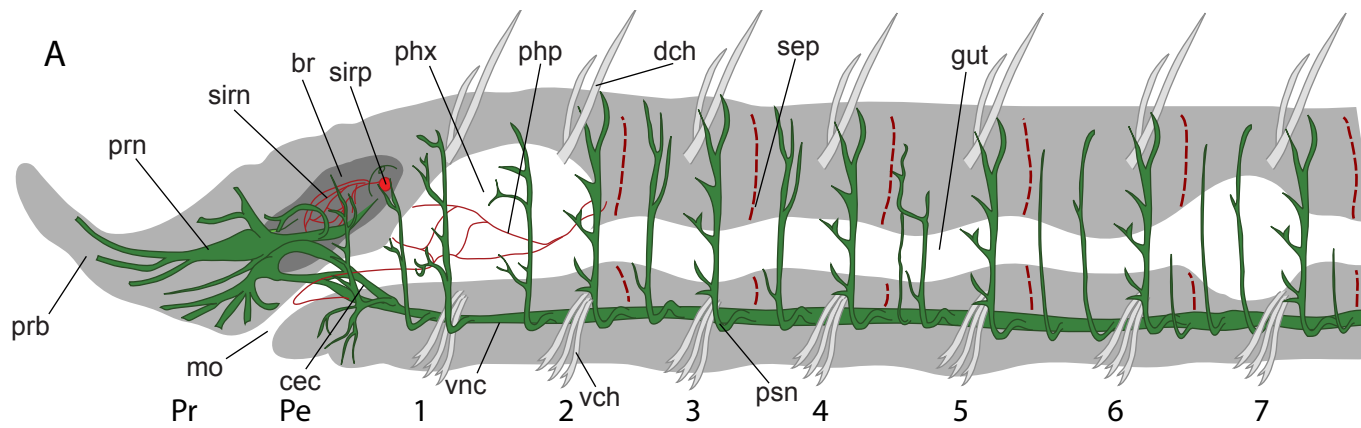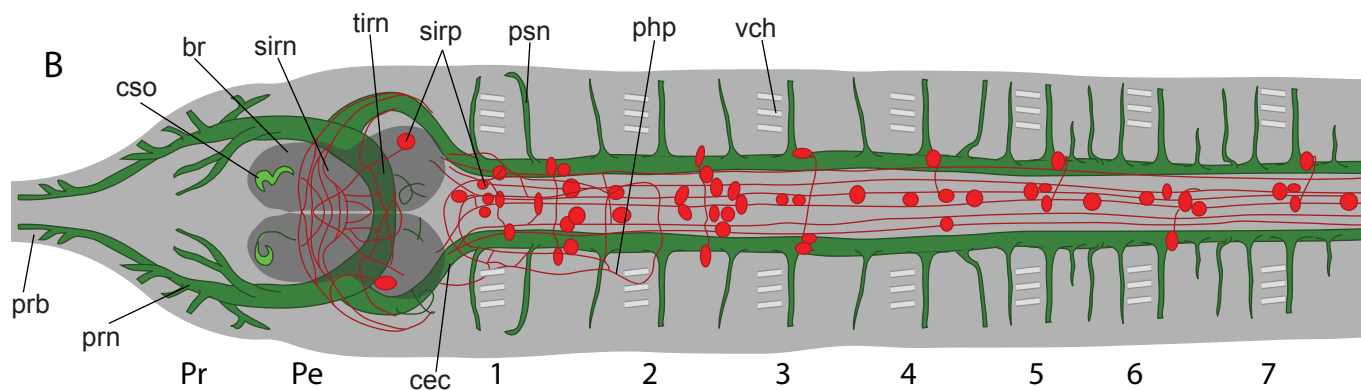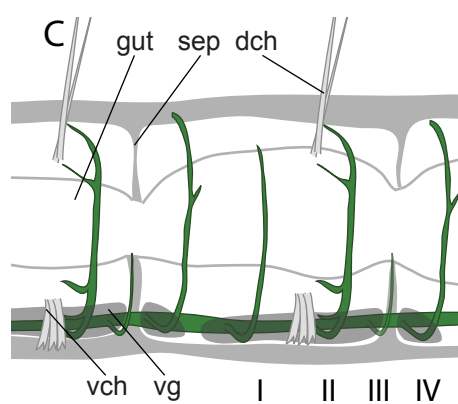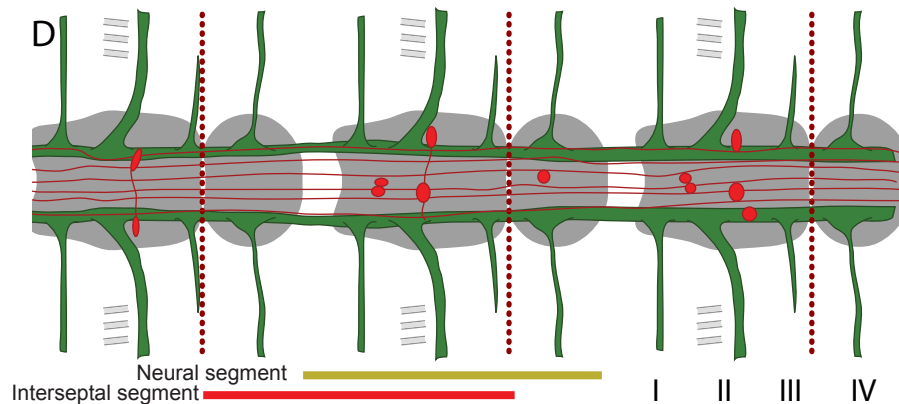

Supplement: Additional file 2: Figure S2. — Nervous system of Pristina aequiseta (Clitellata: Naididae: Pristininae). Drawings based on specimens prepared and labeled as in Additional file 1: Figure S1. A) Lateral view of the anterior end showing ventral nerve cord, segmental peripheral nerves, prostomial nerves, circumesophageal connective and brain. Serotonin immunoreactive (SIR) structures shown only for brain and pharyngeal plexus. B) Dorsal view of the anterior end, showing same structures as A, plus SIR elements in ventral nerve cord and pharyngeal plexus. C) Lateral view of a typical trunk body segment showing localization of peripheral nerve roots relative to ganglia, septa and chaetae; SIR elements not shown. D) Schematic of the structure of the ventral nerve cord showing localization of peripheral nerve roots relative to ganglia, septa and chaetae. Abbreviations as in Additional file 1: Figure S1. [file 12983_2015_100_MOESM2_ESM.pdf]

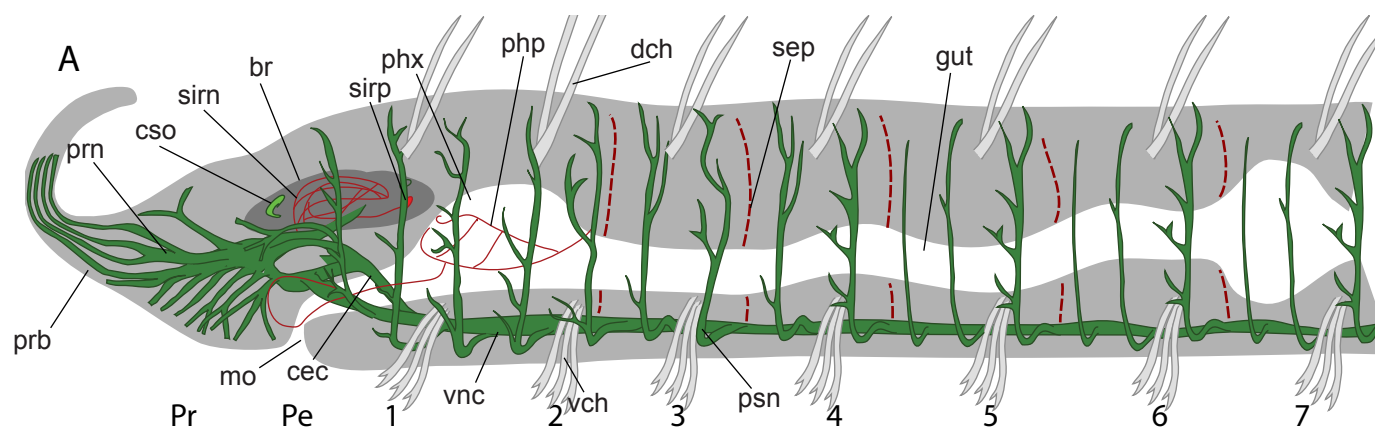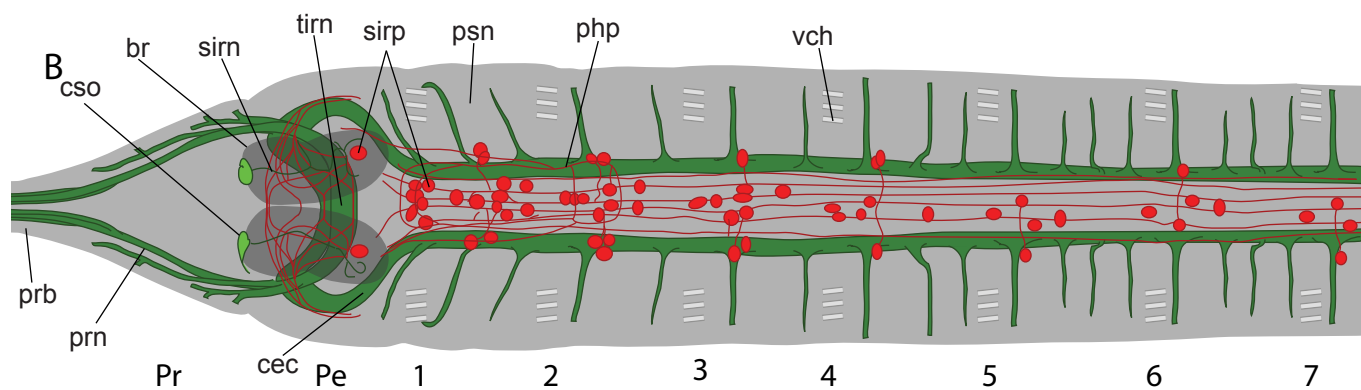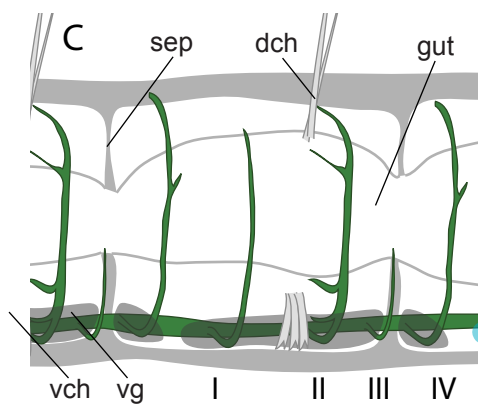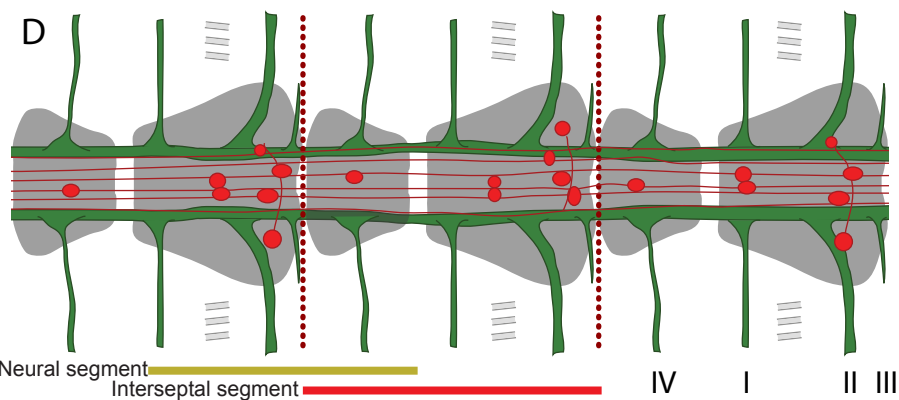

Supplement: Additional file 3: Figure S3. — Nervous system of Pristina leidyi (Clitellata: Naididae: Pristininae). Drawings based on specimens prepared and labeled as in Additional file 1: Figure S1. A) Lateral view of the anterior end showing ventral nerve cord, segmental peripheral nerves, prostomial nerves, circumesophageal connective and brain. Serotonin immunoreactive (SIR) structures shown only for brain and pharyngeal plexus. B) Dorsal view of the anterior end, showing same structures as A, plus SIR elements in ventral nerve cord and pharyngeal plexus. C) Lateral view of a typical trunk body segment showing localization of peripheral nerve roots relative to ganglia, septa and chaetae; SIR elements not shown. D) Schematic of the structure of the ventral nerve cord showing localization of peripheral nerve roots relative to ganglia, septa and chaetae. Abbreviations as in Additional file 1: Figure S1. [file 12983_2015_100_MOESM3_ESM.pdf]

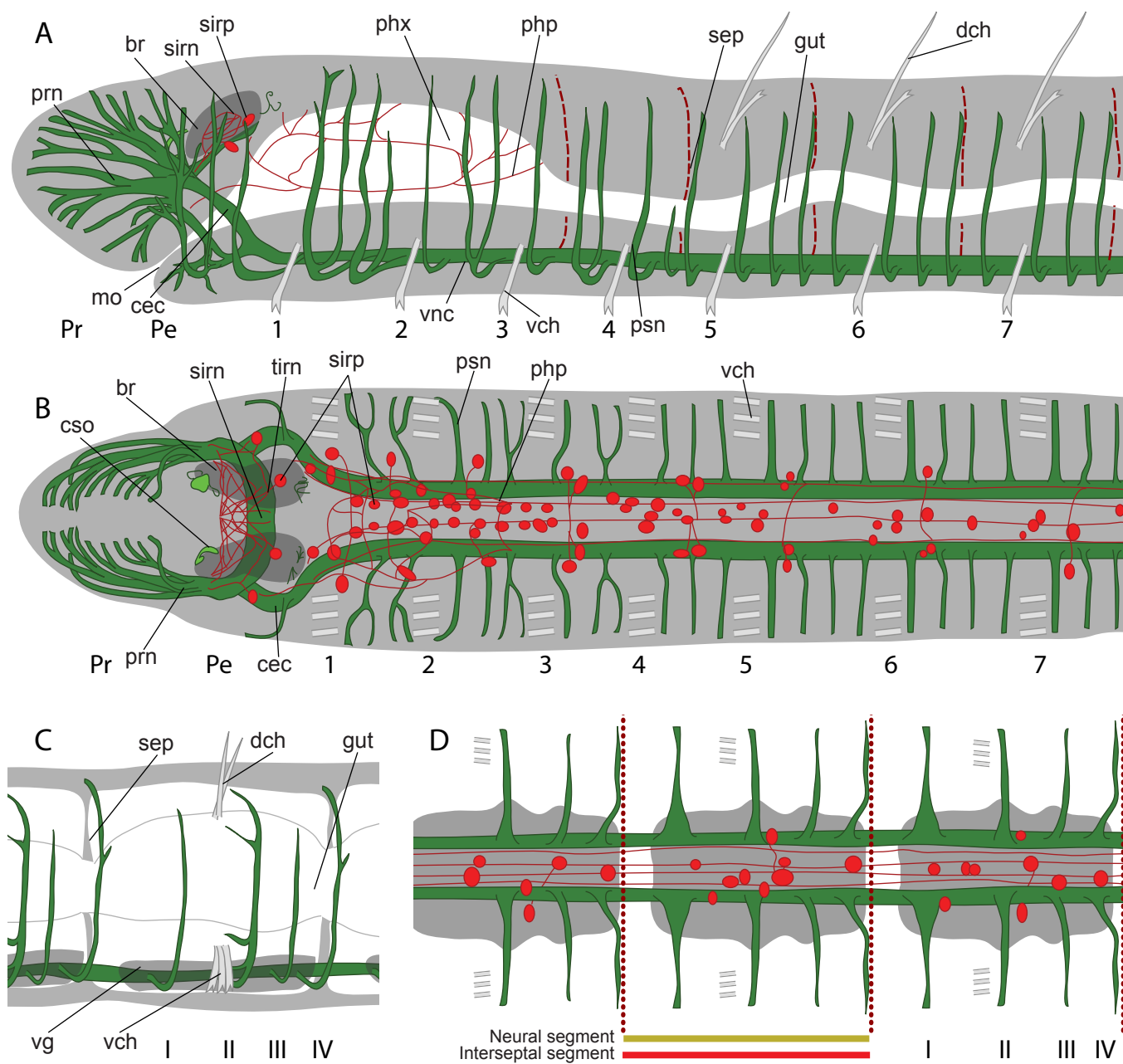

Supplement: Additional file 4: Figure S4. — Nervous system of Dero digitata (Clitellata: Naididae: Naidinae). Drawings based on specimens prepared and labeled as in Additional file 1: Figure S1. A) Lateral view of the anterior end showing ventral nerve cord, segmental peripheral nerves, prostomial nerves, circumesophageal connective and brain. Serotonin immunoreactive (SIR) structures shown only for brain and pharyngeal plexus. B) Dorsal view of the anterior end, showing same structures as A, plus SIR elements in ventral nerve cord and pharyngeal plexus. C) Lateral view of a typical trunk body segment showing localization of peripheral nerve roots relative to ganglia, septa and chaetae; SIR elements not shown except for lateral subepidermal perikarya. D) Schematic of the structure of the ventral nerve cord showing localization of peripheral nerve roots relative to ganglia, septa and chaetae. Abbreviations as in Additional file 1: Figure S1. [file 12983_2015_100_MOESM4_ESM.pdf]

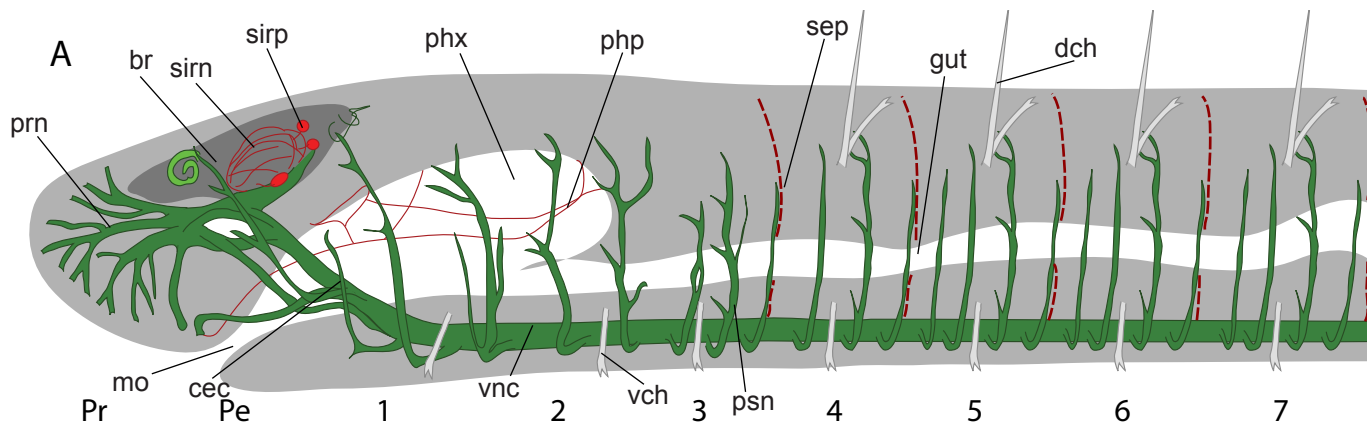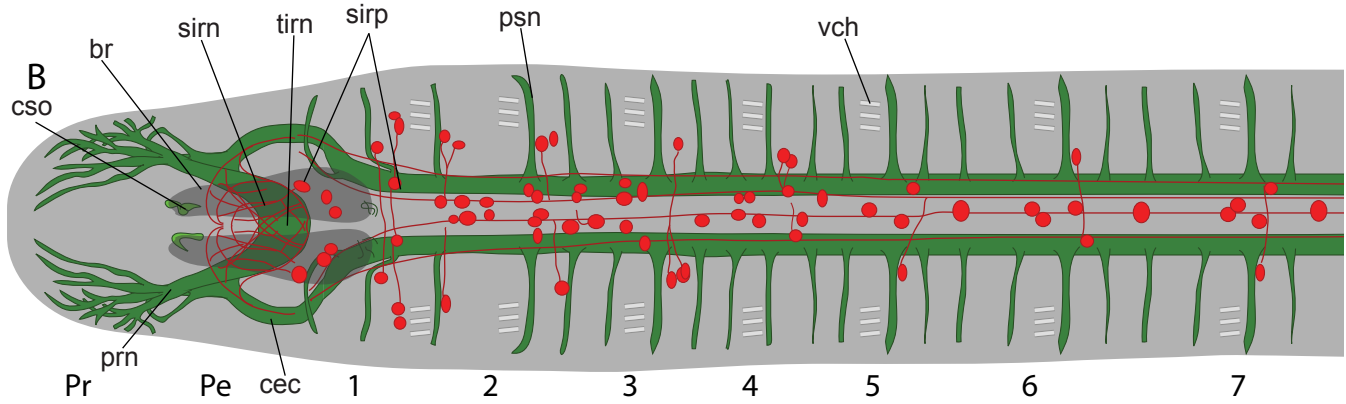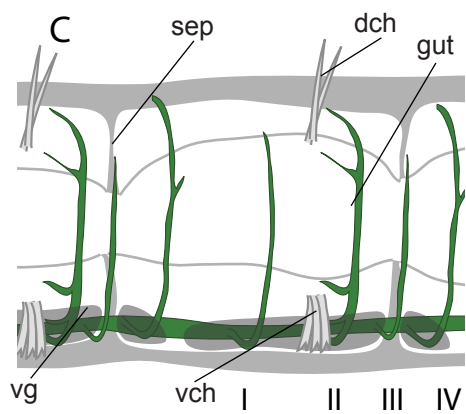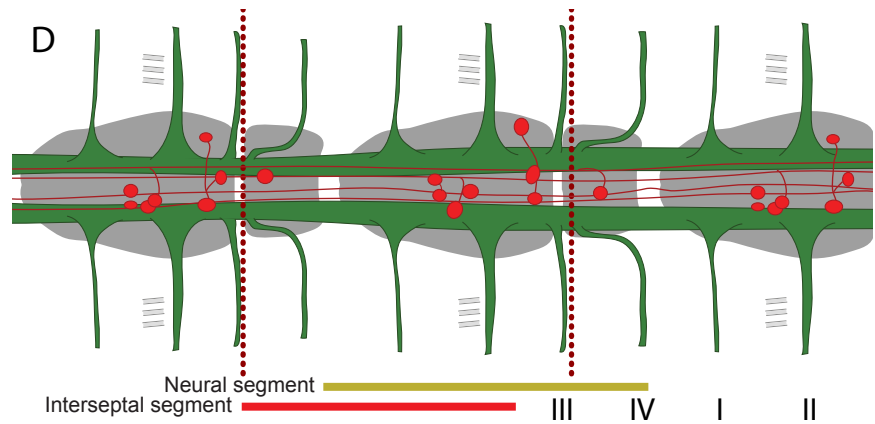

Supplement: Additional file 5: Figure S5. — Nervous system of Dero furcata (Clitellata: Naididae: Naidinae). Drawings based on specimens prepared and labeled as in Additional file 1: Figure S1. A) Lateral view of the anterior end showing ventral nerve cord, segmental peripheral nerves, prostomial nerves, circumesophageal connective and brain. Serotonin immunoreactive (SIR) structures shown only for brain and pharyngeal plexus. B) Dorsal view of the anterior end, showing same structures as A, plus SIR elements in ventral nerve cord and pharyngeal plexus. C) Lateral view of a typical trunk body segment showing localization of peripheral nerve roots relative to ganglia, septa and chaetae; SIR elements not shown except for lateral subepidermal perikarya. D) Schematic of the structure of the ventral nerve cord showing localization of peripheral nerve roots relative to ganglia, septa and chaetae. Abbreviations as in Additional file 1: Figure S1. [file 12983_2015_100_MOESM5_ESM.pdf]

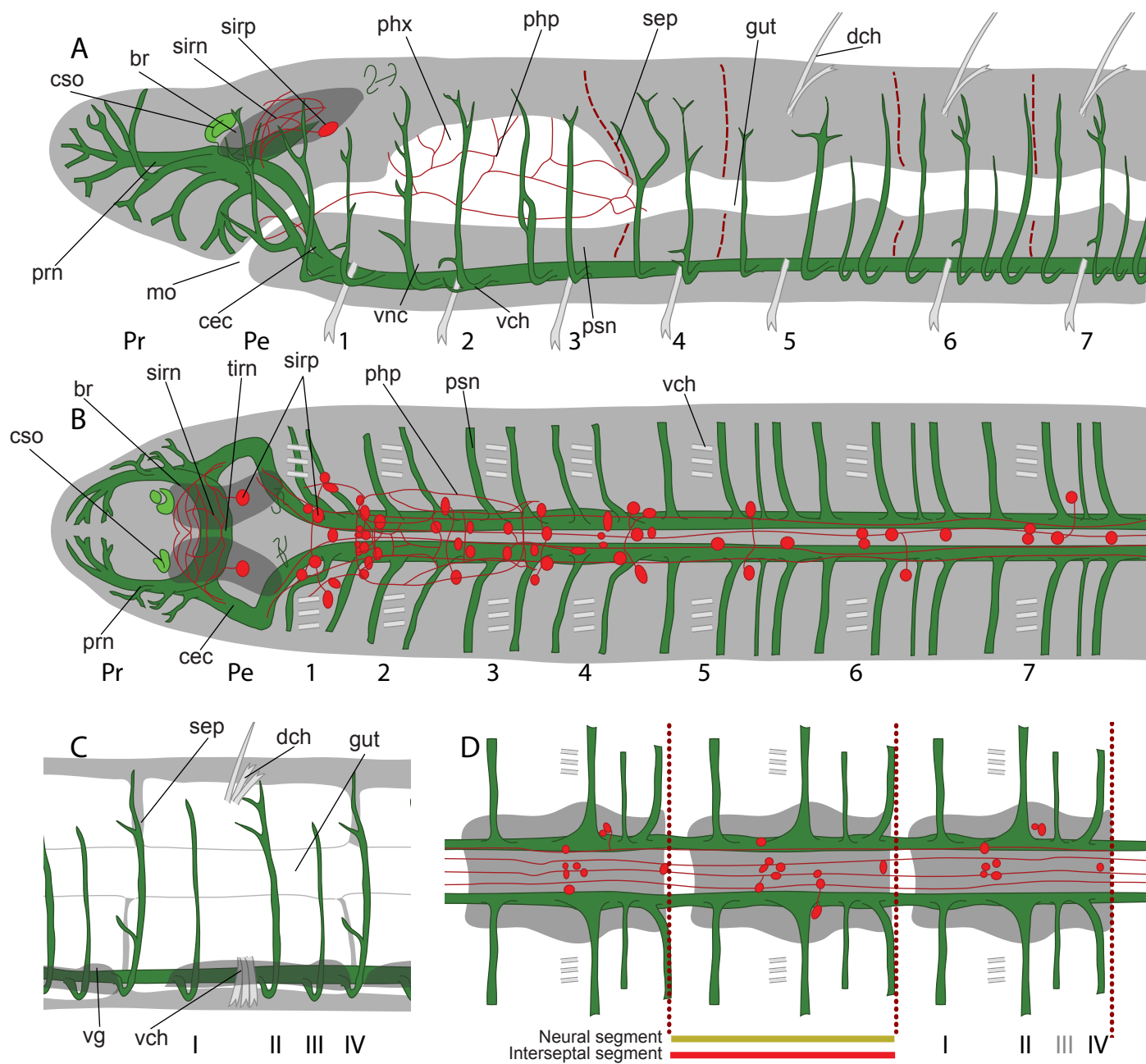

Supplement: Additional file 6: Figure S6. — Nervous system of Allonais paraguayensis (Clitellata: Naididae: Naidinae). Drawings based on specimens prepared and labeled as in Additional file 1: Figure S1. A) Lateral view of the anterior end showing ventral nerve cord, segmental peripheral nerves, prostomial nerves, circumesophageal connective and brain. Serotonin immunoreactive (SIR) structures shown only for brain and pharyngeal plexus. B) Dorsal view of the anterior end, showing same structures as A, plus SIR elements in ventral nerve cord and pharyngeal plexus. C) Lateral view of a typical trunk body segment showing localization of peripheral nerve roots relative to ganglia, septa and chaetae. D) Schematic of the structure of the ventral nerve cord showing localization of peripheral nerve roots relative to ganglia, septa and chaetae. Abbreviations as in Additional file 1: Figure S1. [file 12983_2015_100_MOESM6_ESM.pdf]

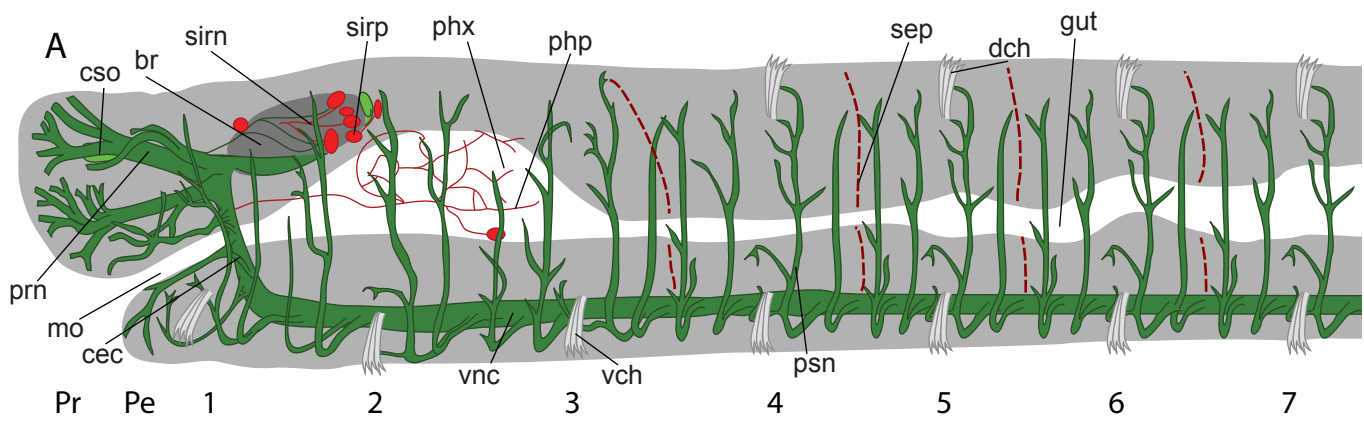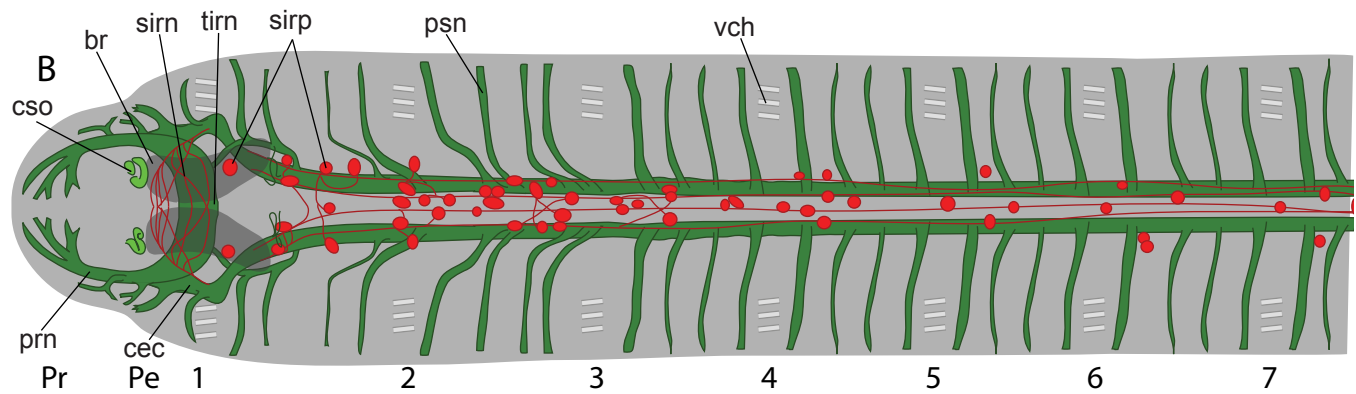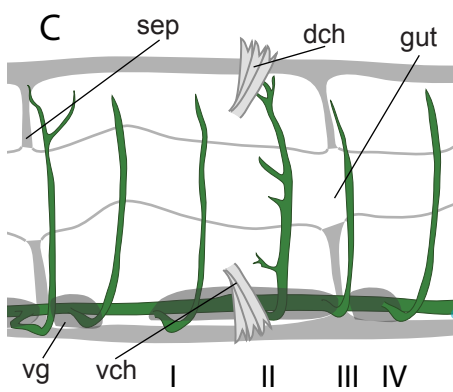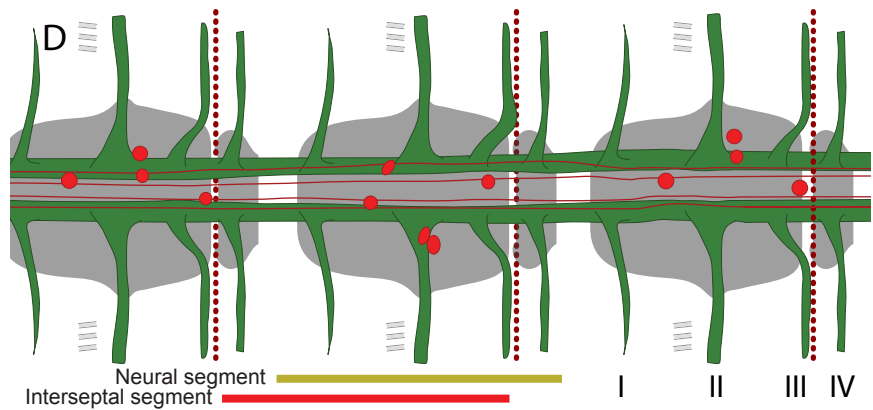

Supplement: Additional file 7: Figure S7. — Nervous system of Paranais litoralis (Clitellata: Naididae: Naidinae). Drawings based on specimens prepared and labeled as in Additional file 1: Figure S1. A) Lateral view of the anterior end showing ventral nerve cord, segmental peripheral nerves, prostomial nerves, circumesophageal connective and brain. Serotonin immunoreactive (SIR) structures shown only for brain and pharyngeal plexus. B) Dorsal view of the anterior end, showing same structures as A, plus SIR elements in ventral nerve cord and pharyngeal plexus. C) Lateral view of a typical trunk body segment showing localization of peripheral nerve roots relative to ganglia, septa and chaetae. D) Schematic of the structure of the ventral nerve cord showing localization of peripheral nerve roots relative to ganglia, septa and chaetae. Abbreviations as in Additional file 1: Figure S1. [file 12983_2015_100_MOESM7_ESM.pdf]

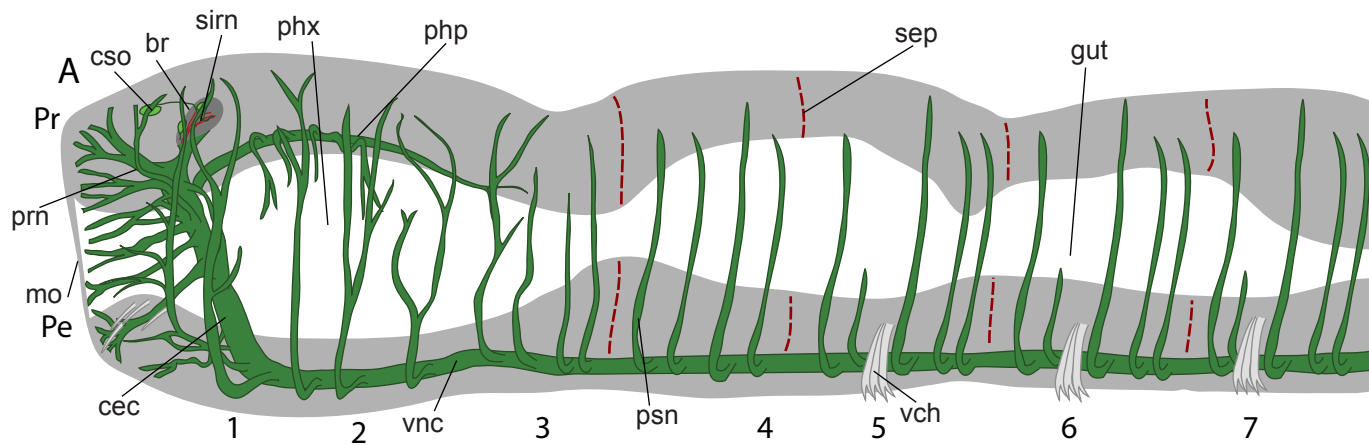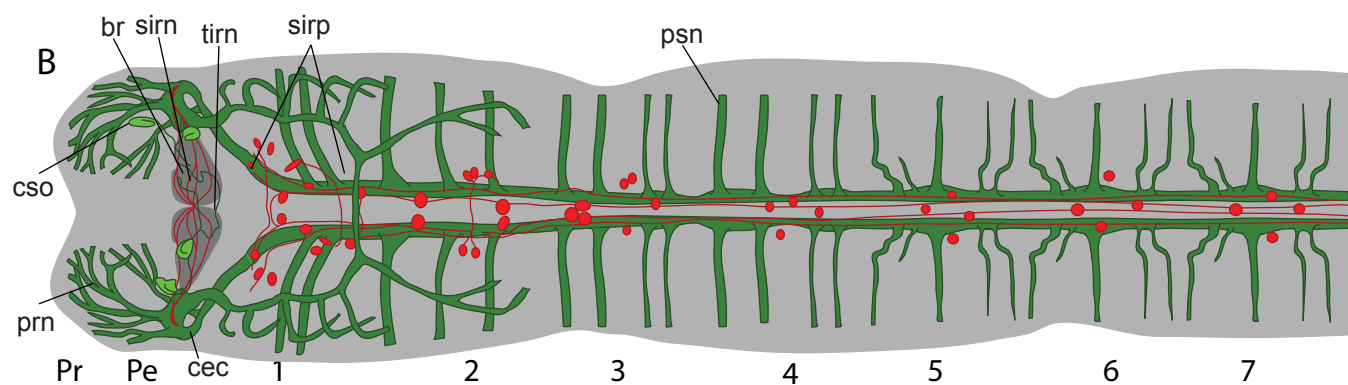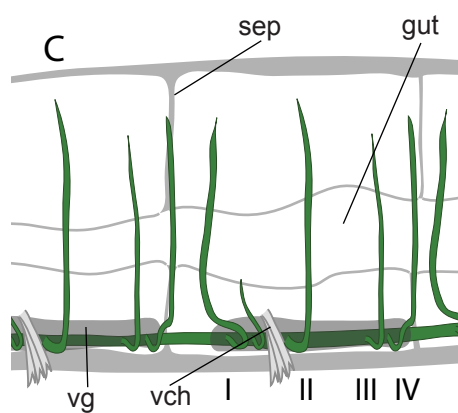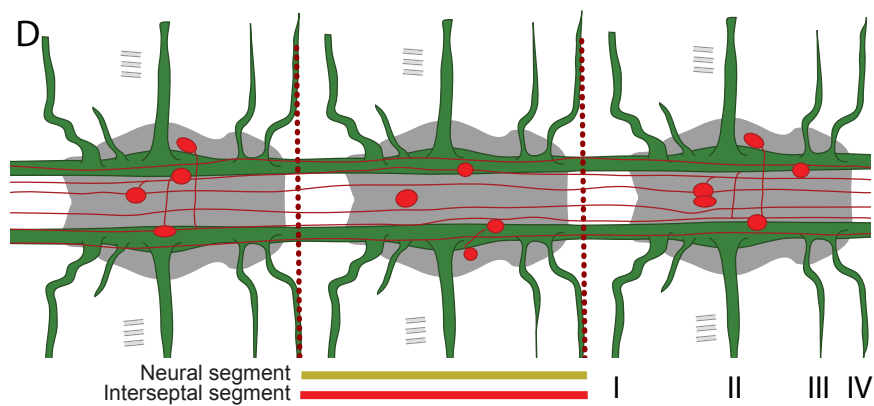

Supplement: Additional file 8: Figure S8. — Nervous system of Chaetogaster diaphanus (Clitellata: Naididae: Naidinae). Drawings based on specimens prepared and labeled as in Additional file 1: Figure S1. A) Lateral view of the anterior end showing ventral nerve cord, segmental peripheral nerves, prostomial nerves, circumesophageal connective and brain. Serotonin immunoreactive (SIR) structures shown only for brain. B) Dorsal view of the anterior end, showing same structures as A, plus SIR elements in ventral nerve cord and pharyngeal plexus. C) Lateral view of a typical trunk body segment showing localization of peripheral nerve roots relative to ganglia, septa and chaetae; SIR elements not shown. D) Schematic of the structure of the ventral nerve cord showing localization of peripheral nerve roots relative to ganglia, septa and chaetae. Abbreviations as in Additional file 1: Figure S1. [file 12983_2015_100_MOESM8_ESM.pdf]

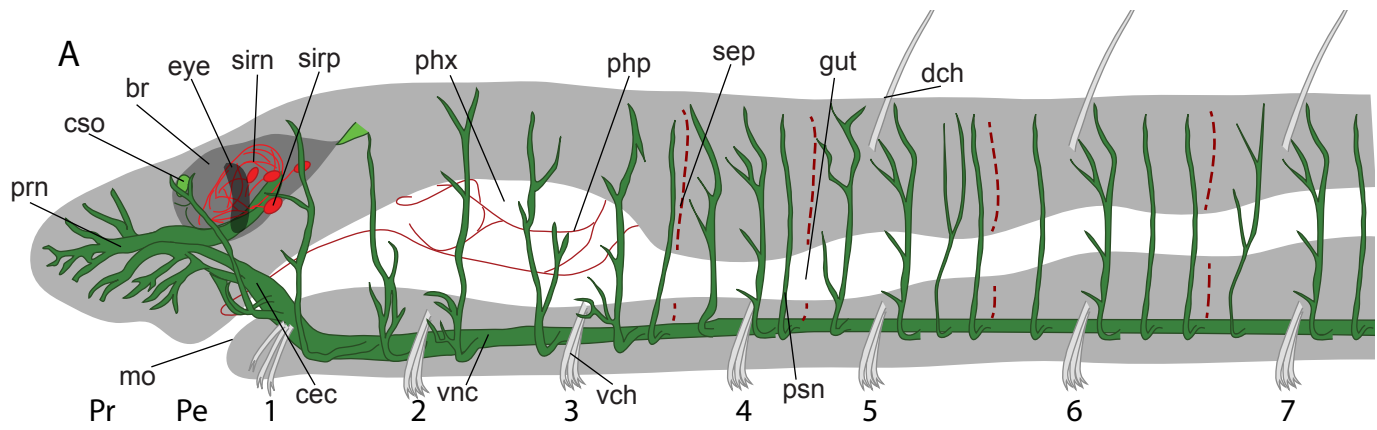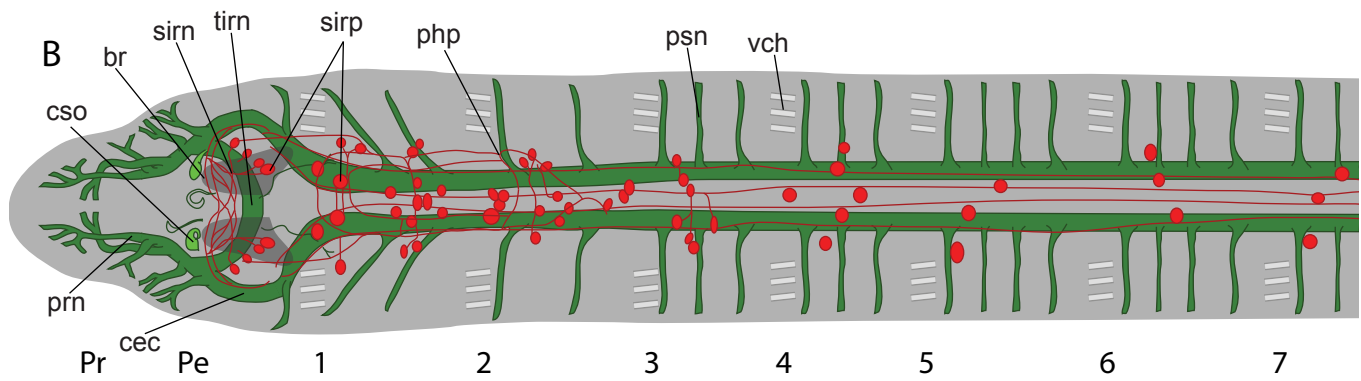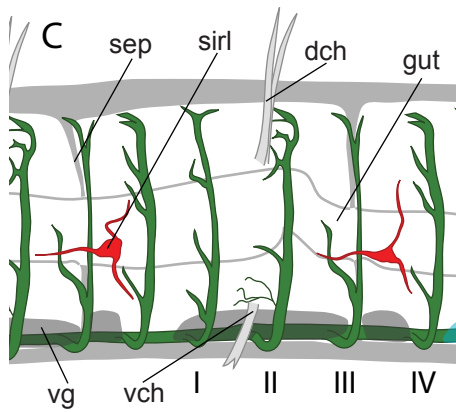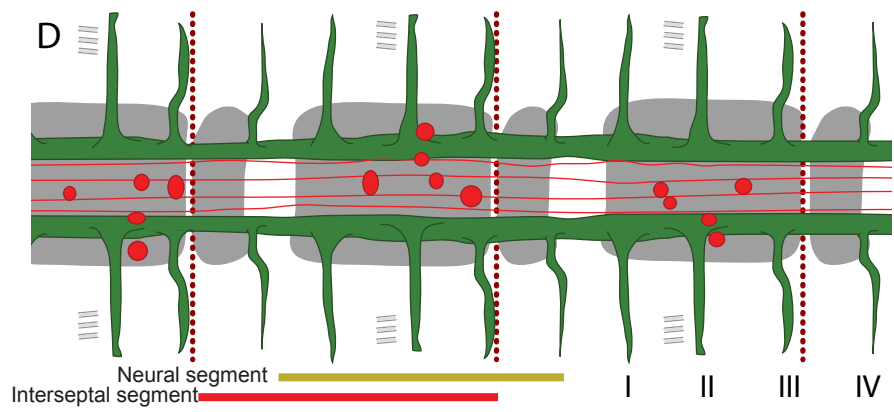

Supplement: Additional file 9: Figure S9. — Nervous system of Nais stolci (Clitellata: Naididae: Naidinae). Drawings based on specimens prepared and labeled as in Additional file 1: Figure S1. A) Lateral view of the anterior end showing ventral nerve cord, segmental peripheral nerves, prostomial nerves, circumesophageal connective and brain. Serotonin immunoreactive (SIR) structures shown only for brain and pharyngeal plexus. B) Dorsal view of the anterior end, showing same structures as A, plus SIR elements in ventral nerve cord and pharyngeal plexus. C) Lateral view of a typical trunk body segment showing localization of peripheral nerve roots relative to ganglia, septa and chaetae; SIR elements not shown except for lateral subepidermal perikarya. D) Schematic of the structure of the ventral nerve cord showing localization of peripheral nerve roots relative to ganglia, septa and chaetae. Abbreviations as in Additional file 1: Figure S1. [file 12983_2015_100_MOESM9_ESM.pdf]

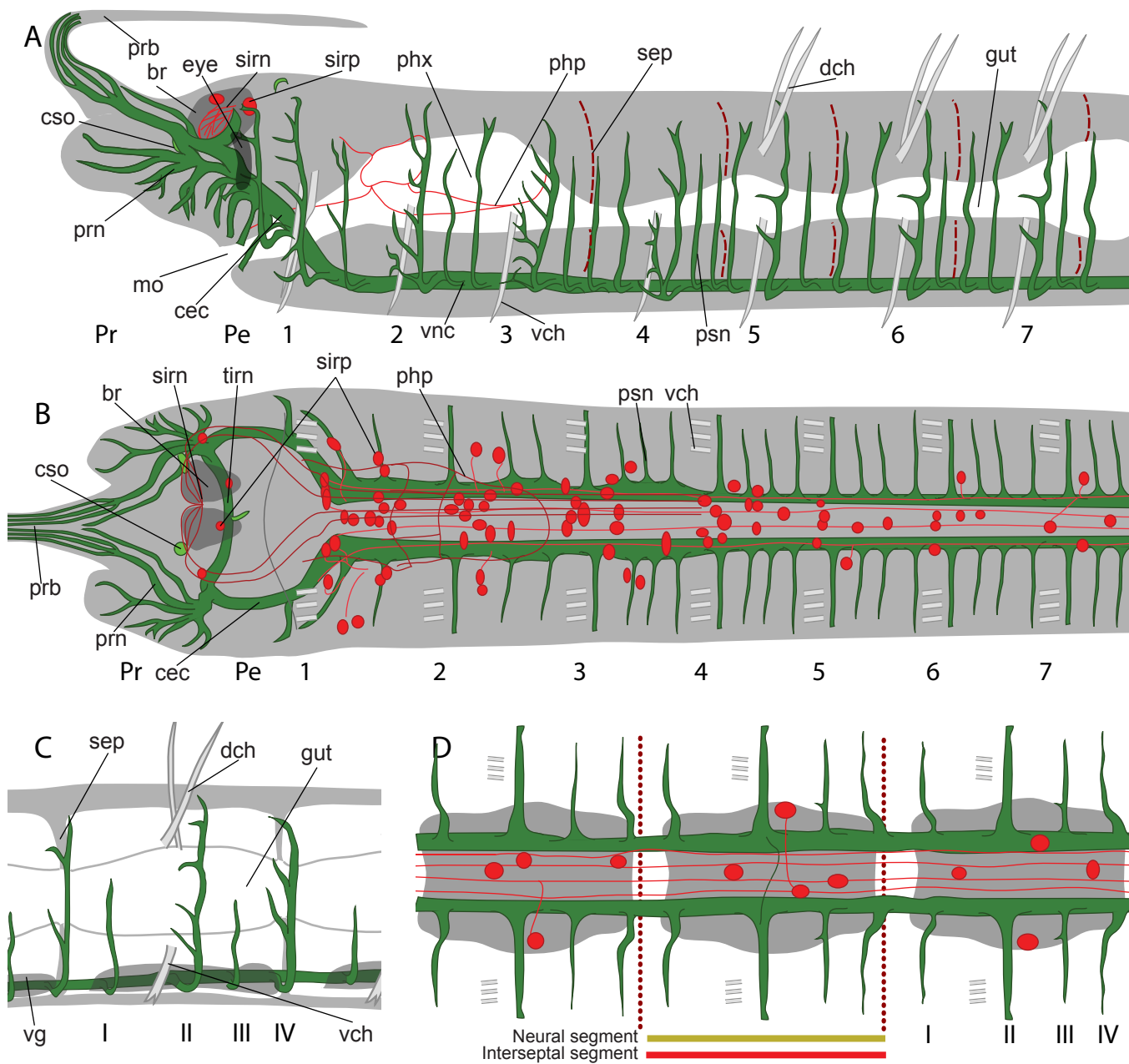

Supplement: Additional file 10: Figure S10. — Nervous system of Stylaria lacustris (Clitellata: Naididae: Naidinae). Drawings based on specimens prepared and labeled as in Additional file 1: Figure S1. A) Lateral view of the anterior end showing ventral nerve cord, segmental peripheral nerves, prostomial nerves, circumesophageal connective and brain. Serotonin immunoreactive (SIR) structures shown only for brain and pharyngeal plexus. B) Dorsal view of the anterior end, showing same structures as A, plus SIR elements in ventral nerve cord and pharyngeal plexus. C) Lateral view of a typical trunk body segment showing localization of peripheral nerve roots relative to ganglia, septa and chaetae. D) Schematic of the structure of the ventral nerve cord showing localization of peripheral nerve roots relative to ganglia, septa and chaetae. Abbreviations as in Additional file 1: Figure S1. [file 12983_2015_100_MOESM10_ESM.pdf]

A

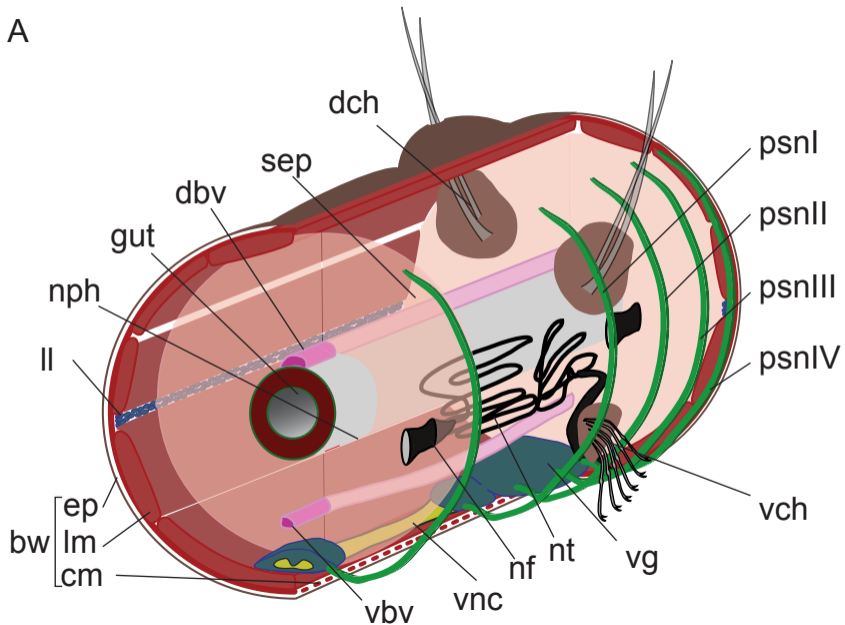

B

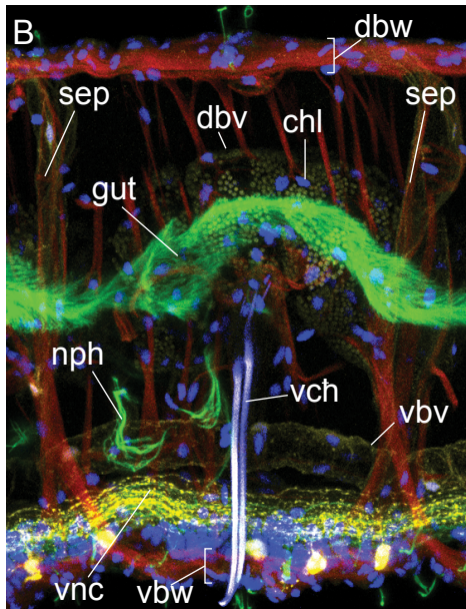

Supplement: Additional file 11: Figure S11. — Naidid segment ground plan. A) Stereogram of a generic naidid segmental unit. B) Internal structure of a body segment. Parasagittal maximum intensity projection of a lateral Z-stack of Stylaria lacustris stained for DNA (blue - nuclei), F-actin (red - muscle), acetyl-tubulin (green - primarily peripheral nervous system and ciliated structures (e.g., nephridia and gut)) and serotonin (yellow - primarily ventral nerve cord). Labels: bw: body wall; chl: chloragogen cells; cm: circular muscle; dbv: dorsal blood vessel; dbw: dorsal body wall; dch: dorsal chaetae; ep: epidermis; gut: ciliated gut; ll: lateral line; lm: longitudinal muscle; nf: nephridial funnel; nph: nephridium; nt: nephrotubule; pnI-IV: peripheral segmental nerve I-IV; sep: intersegmental septum; vbv: ventral blood vessel; vbw: ventral body wall; vch: ventral chaetae; vg: ventral ganglion; vnc: ventral nerve cord. [file 12983_2015_100_MOESM11_ESM.pdf]

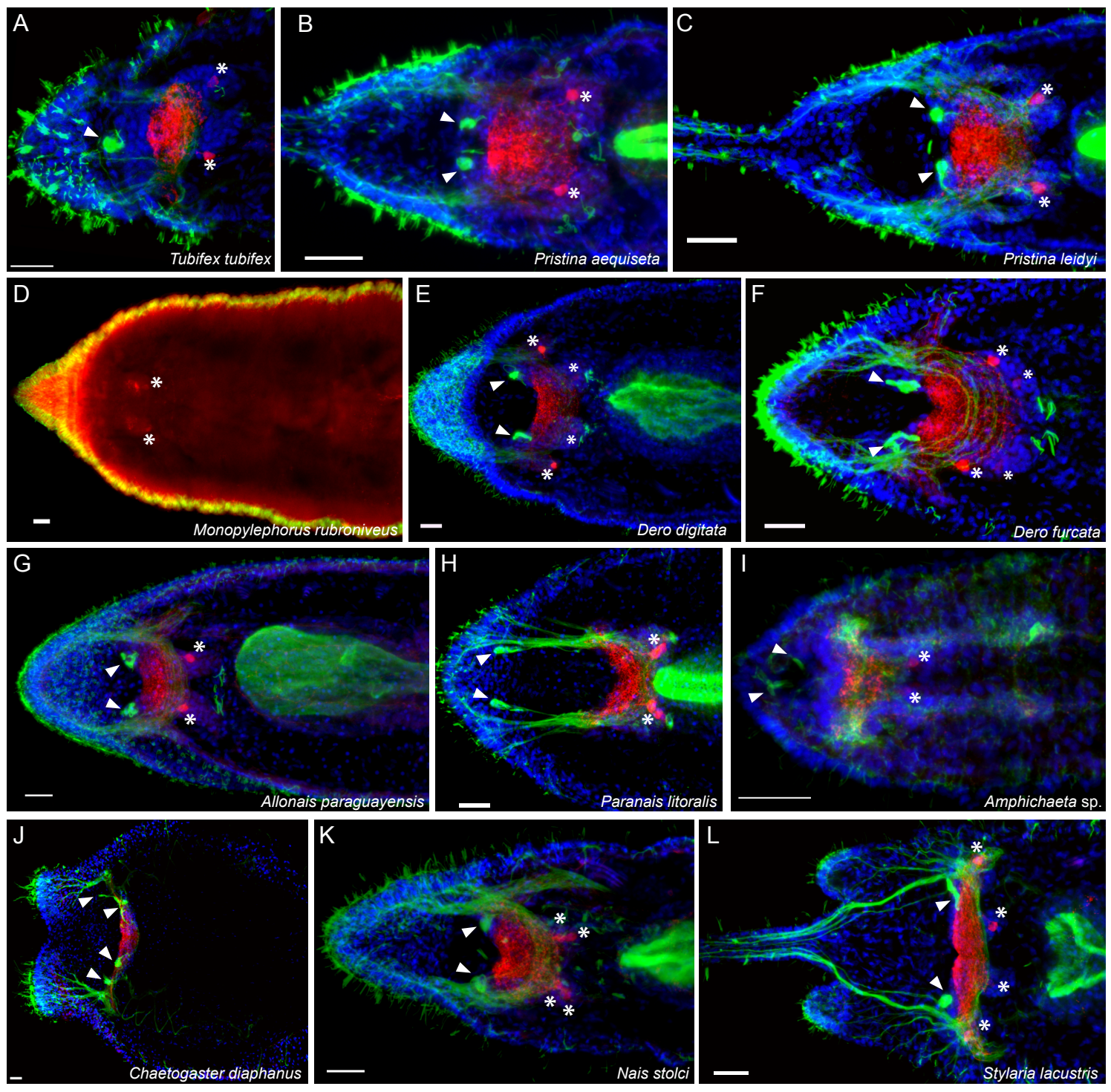

Supplement: Additional file 12: Figure S12. — Diversity in anterior nervous system morphology of 12 species of Naididae. Note the variation in the position and number of ciliary sense organs (arrowheads) relative to the SIR brain neuropil, visible as a red mass of SIR neurites; note also the location and number of SIR perikarya (asterisks). Images are intensity sum projections of dorsal Z-stacks of Tubifex tubifex (A), Pristina aequiseta (B), Pristina leidyi (C), Monopylephorus rubroniveus (D), Dero digitata (E), Dero furcata (F), Allonais paraguayensis (G), Paranais litoralis (H), Amphichaeta sp. (I), Chaetogaster diaphanus (J), Nais stolci (K) and Stylaria lacustris (L). Specimens were stained for DNA (blue), serotonin (red) and acetyl-tubulin (green), except for Monopylephorus (D), where DNA was degraded and failed to stain. Scale bars: 25 μm. [file 12983_2015_100_MOESM12_ESM.pdf]

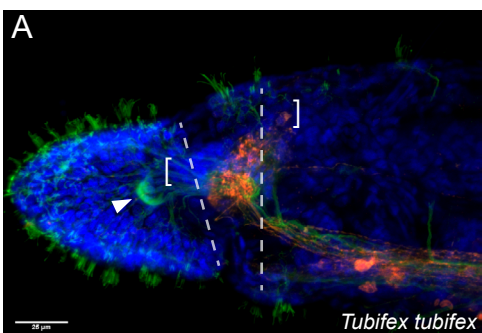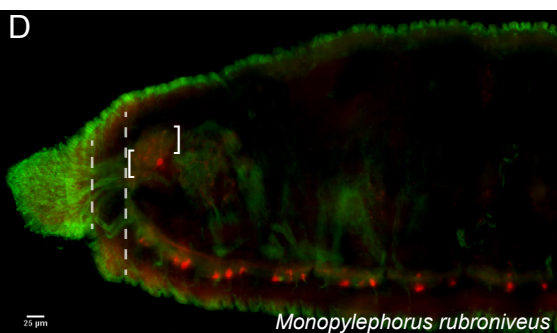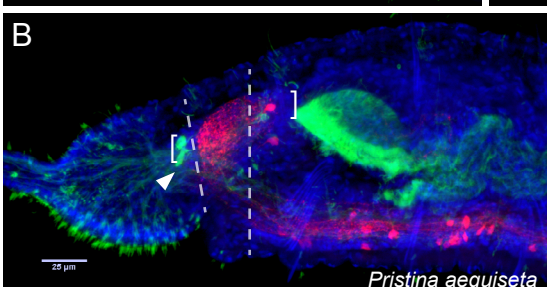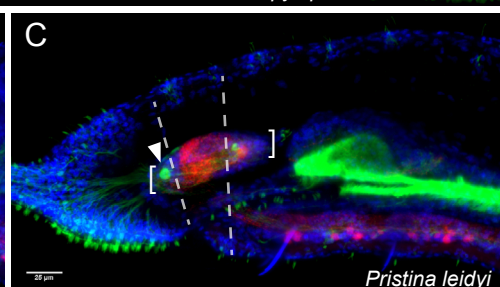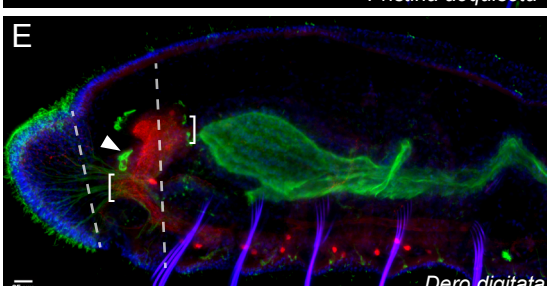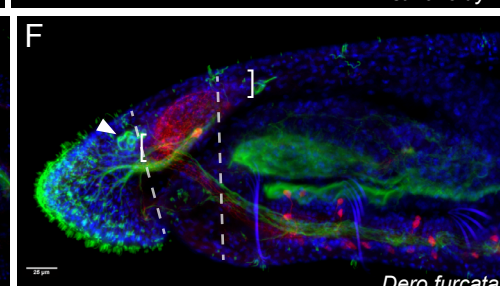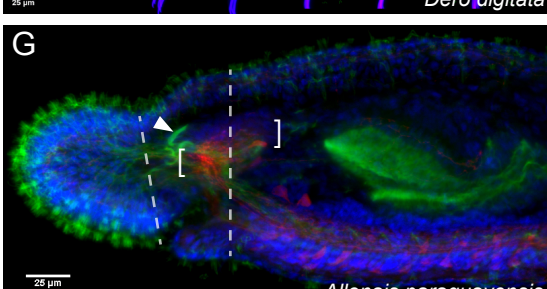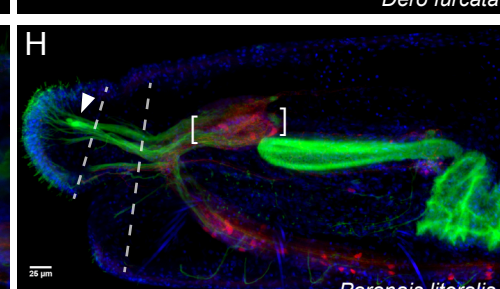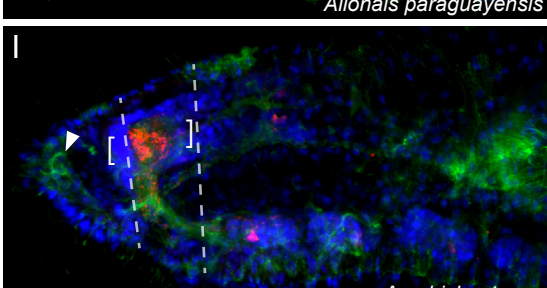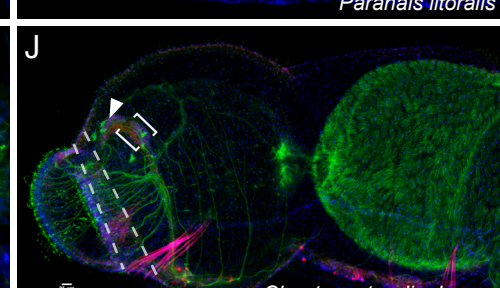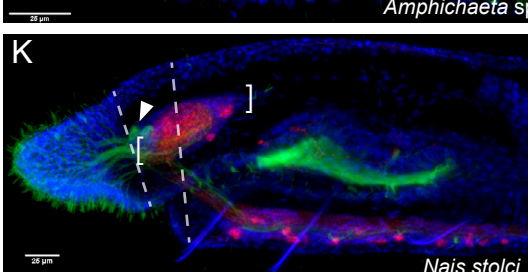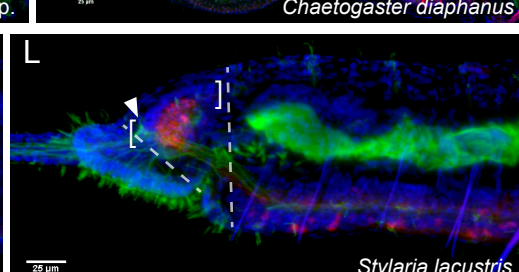

Supplement: Additional file 13: Figure S13. — Variation in position of the brain and ciliary sense organs of 12 species of Naididae. Images are intensity sum projection of sagittal Z-stacks. Brain boundaries are shown by paired brackets; approximate prostomium/peristomium and peristomium/segment 1 boundaries are marked by dashed lines; ciliary sense organs are indicated by arrowheads. Tubifex tubifex (A), Pristina aequiseta (B), Pristina leidyi (C), Monopylephorus rubroniveus (D), Dero digitata (E), Dero furcata (F), Allonais paraguayensis (G), Paranais litoralis (H), Amphichaeta sp. (I), Chaetogaster diaphanus (J), Nais stolci (K) and Stylaria lacustris (L). Specimens were stained for DNA (blue), serotonin (red) and acetyl-tubulin (green), except for Monopylephorus (D), where DNA was degraded and failed to stain. Scale bars: 25 μm. [file 12983_2015_100_MOESM13_ESM.pdf]

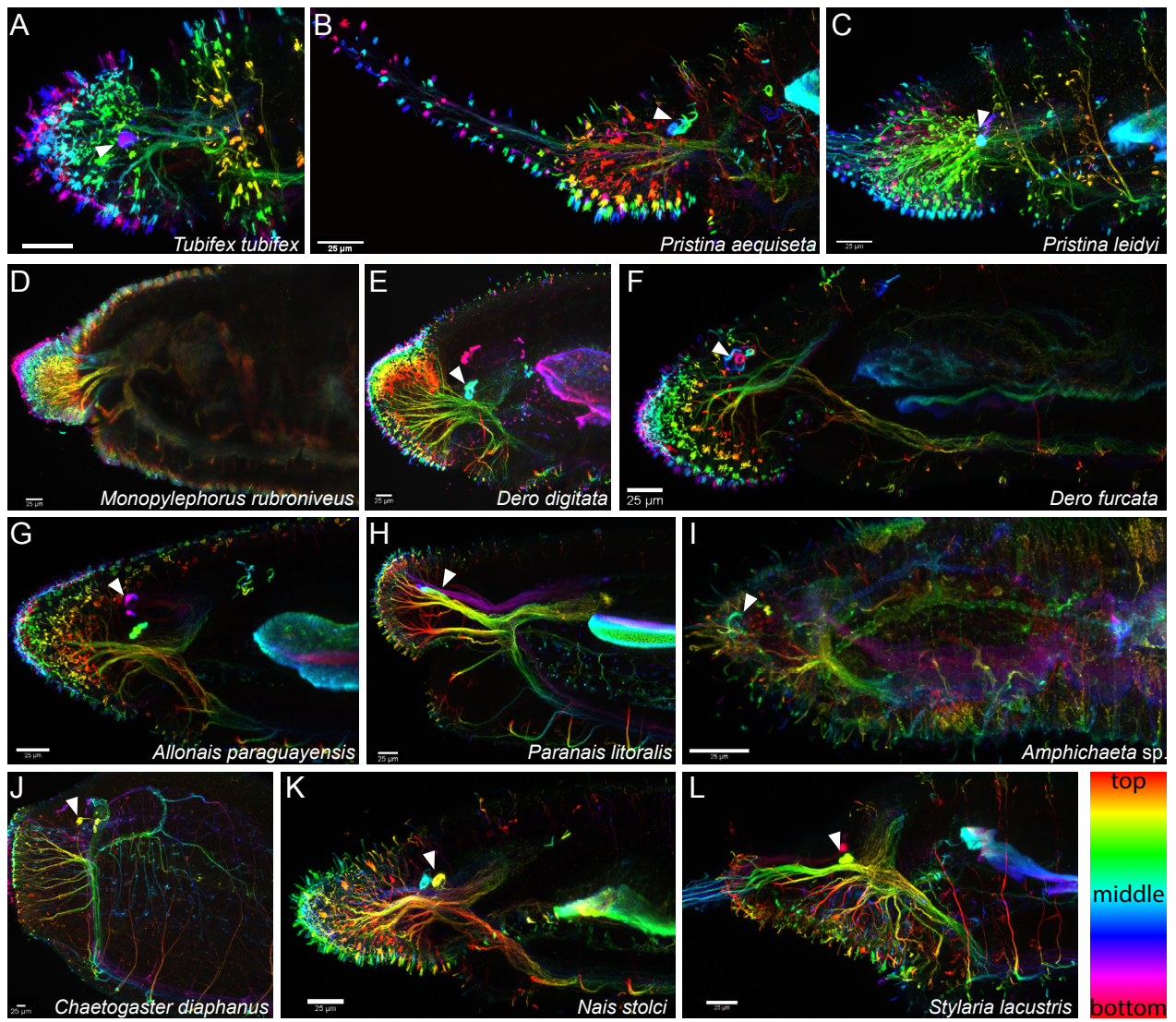

Supplement: Additional file 14: Figure S14. — Diversity in prostomial innervation in 12 species of Naididae. Images are maximum intensity projections of sagittal Z-stacks, color coded by depth along the stack (relative color reference scale at bottom right). All panels show staining for acetylated-tubulin. Ciliary sense organs are indicated by arrowheads. Images are of Tubifex tubifex (A), Pristina aequiseta (B), Pristina leidyi (C), Monopylephorus rubroniveus (D), Dero digitata (E), Dero furcata (F), Allonais paraguayensis (G), Paranais litoralis (H), Amphichaeta sp. (I), Chaetogaster diaphanus (J), Nais stolci (K) and Stylaria lacustris (L). Scale bars: 25 μm. [file 12983_2015_100_MOESM14_ESM.pdf]

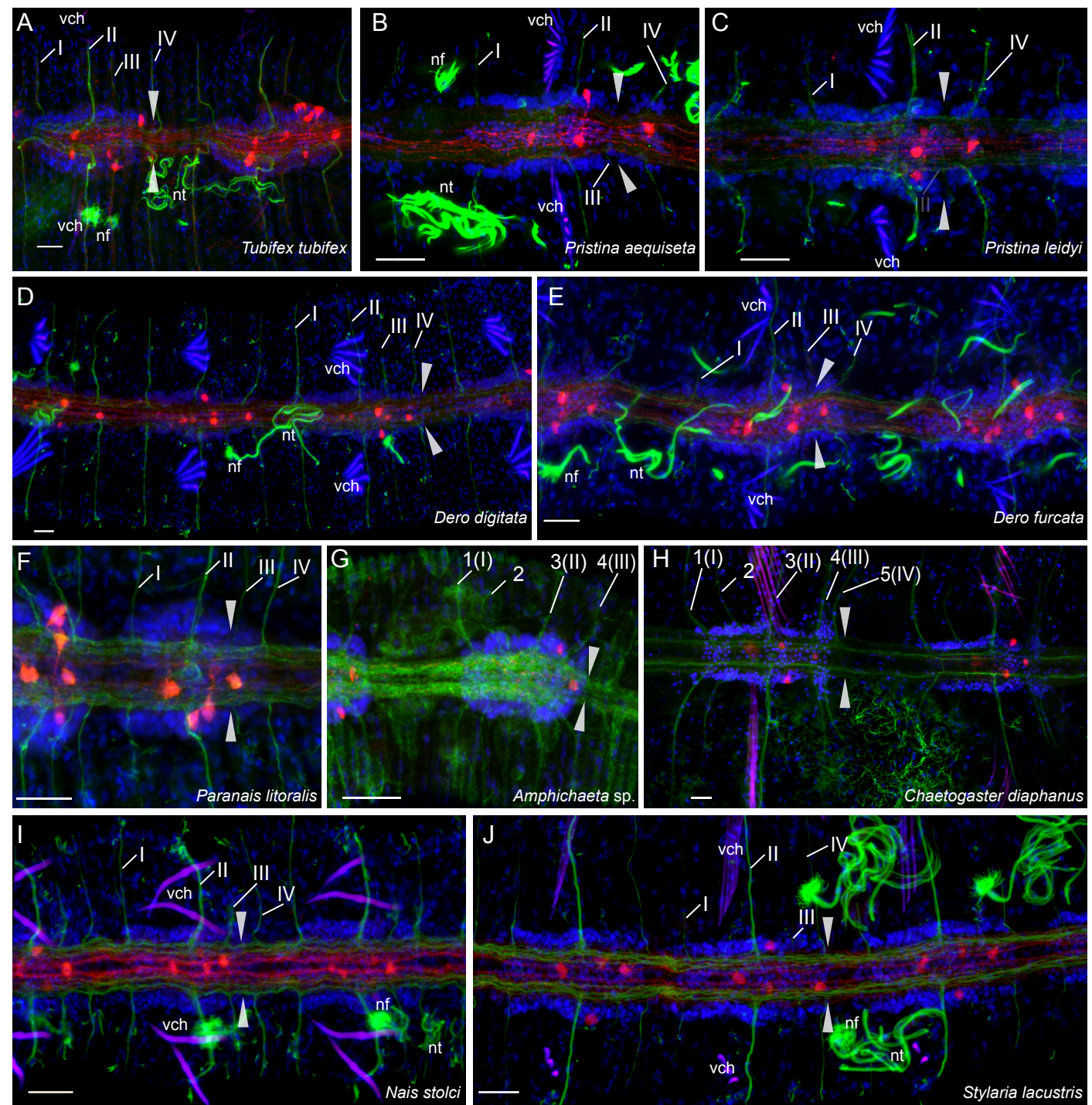

Supplement: Additional file 15: Figure S15. — Diversity of ventral nerve cord ganglion architecture in 10 species of Naididae. Images are intensity sum projections of ventral Z-stacks. Specimens were stained for DNA (blue), serotonin (red) and acetyl-tubulin (green). Segmental nerves are labeled I-IV; alternative arabic numerals are shown in G and H. Ventral chaetae (ch) are visible due to birefringence. The paired arrowheads mark the position of the mesodermal septum. The looping, acetyl-tubulin positive structures in the image corresponds to ciliated nephridia (nf: nephridial funnel; nt: nephrotubule). Tubifex tubifex (A), Pristina aequiseta (B), Pristina leidyi (C), Dero digitata (D), Dero furcata (E), Paranais litoralis (F), Amphichaeta sp. (G), Chaetogaster diaphanus (H), Nais stolci (I) and Stylaria lacustris (J). Monopylephorus is not included here due to failure of DAPI staining and low quality of acITR detection. Scale bars: 25 μm. [file 12983_2015_100_MOESM15_ESM.pdf]

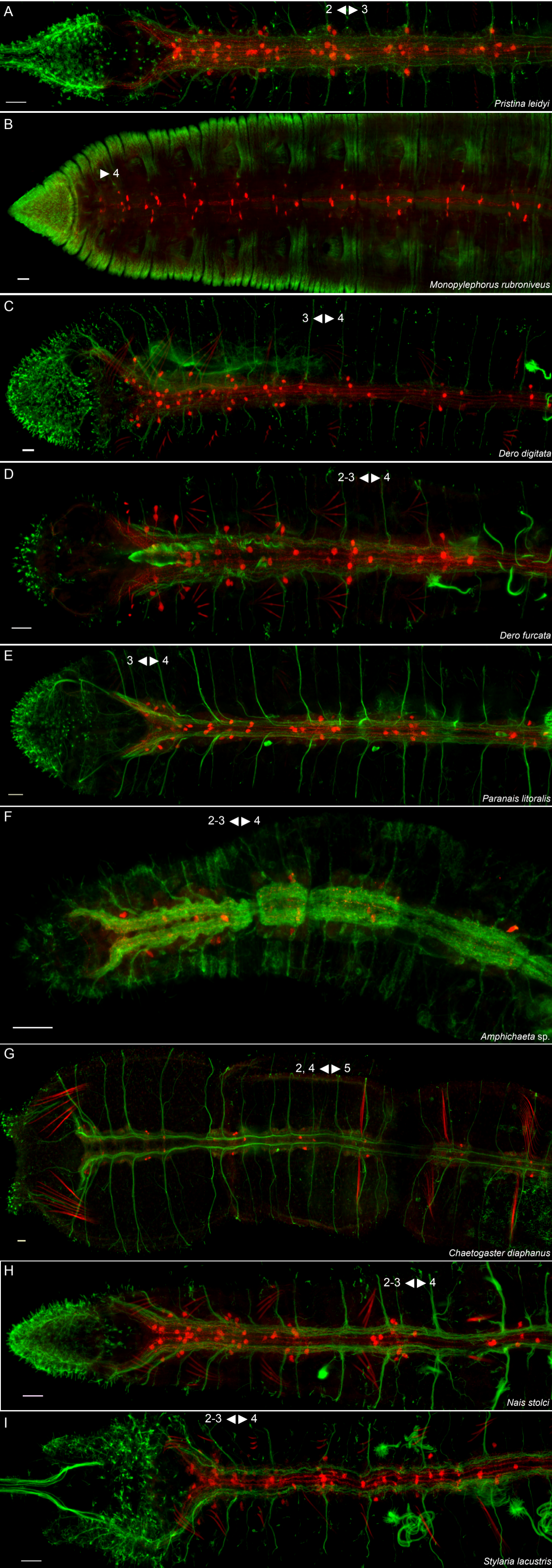

Supplement: Additional file 16: Figure S16. — Central and peripheral nervous system of anterior segments of 9 species of Naididae. Images are intensity sum projections of ventral Z-stacks showing the ventral nerve cord neuropil and segmental peripheral nerves. Labeled acetyl-tubulin immunoreactive nerves (green), and serotonin immunoreactive nerves and perikarya (red) consistently show a different pattern in anterior-most segments relative to more posterior segments, but the level at which this transition occurs varies across species, as well as across the elements of the nervous system. The transition between the anterior and posterior pattern of segmental nerves is indicated by occurs the opposed arrowheads (number of segmental nerves per segment indicated beside arrowheads). Nerve identity is shown below for segments flanking the boundary. Images are from Pristina leidyi (A), Monopylephorus rubroniveus (B), Dero digitata (C), Dero furcata (D), Paranais litoralis (E), Amphichaeta sp. (F), Chaetogaster diaphanus (G), Nais stolci (H) and Stylaria lacustris (I). Scale bars: 25 μm. [file 12983_2015_100_MOESM16_ESM.pdf]

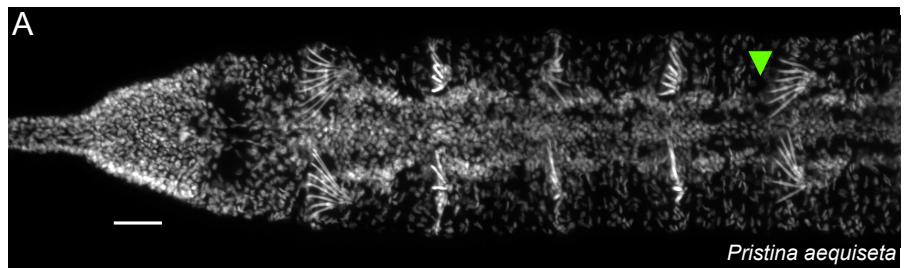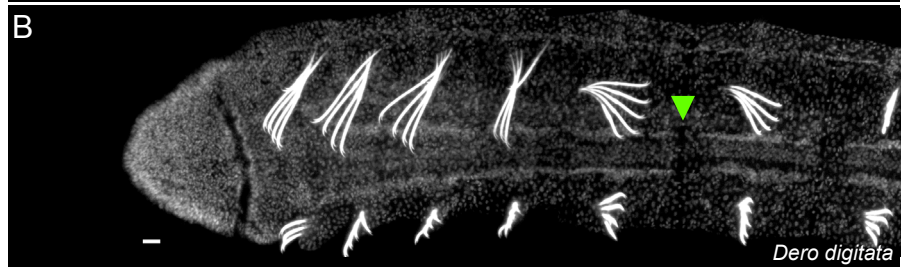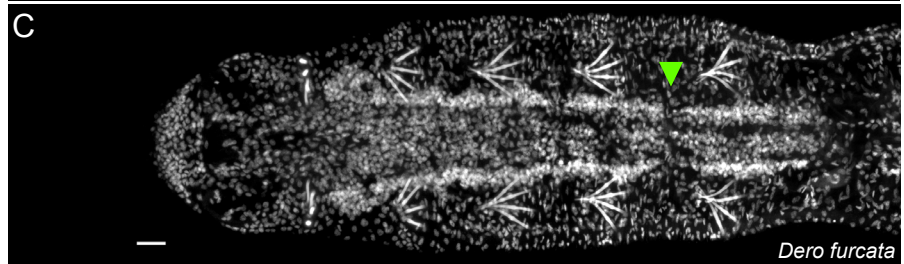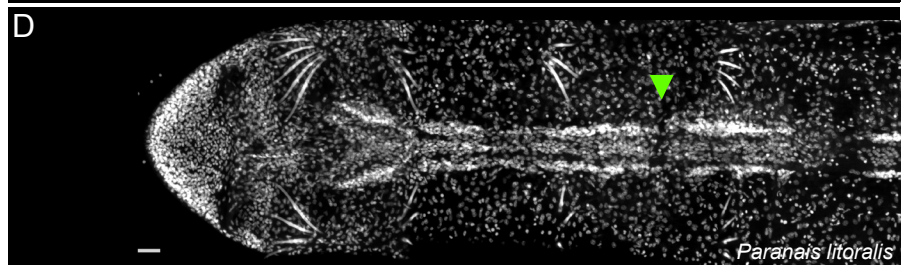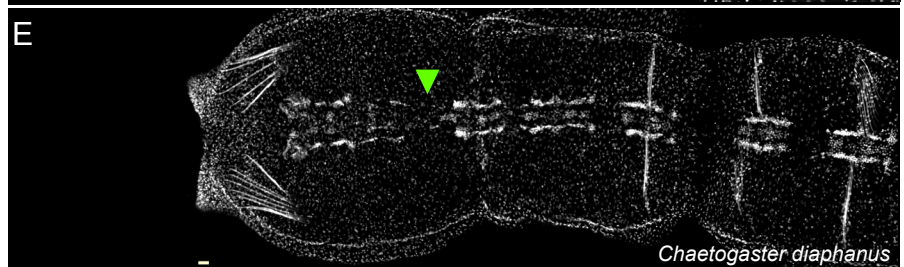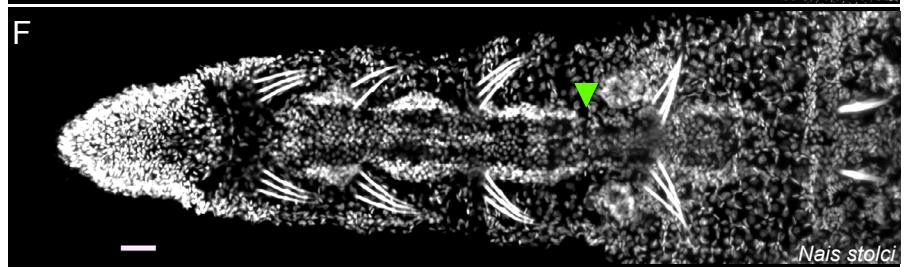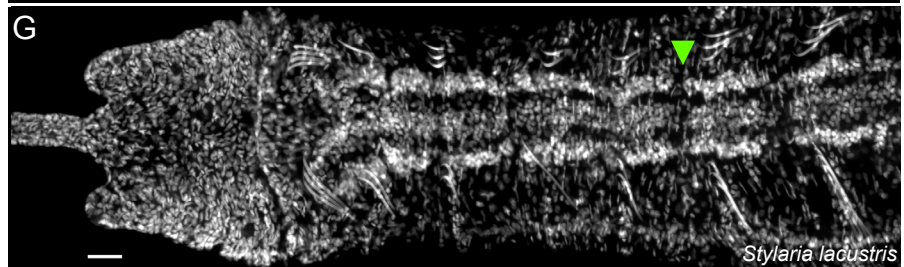

Supplement: Additional file 17: Figure S17. — Diversity in architecture of anterior ventral nerve cord ganglia. Images are intensity sum projections of ventral Z-stacks showing cell nuclei. The green arrowhead marks the location of the anterior-most connective. Images are from Pristina aequiseta (A), Dero digitata (B), Dero furcata (C), Paranais litoralis (D), Chaetogaster diaphanus (E), Nais stolci (F) and Stylaria lacustris (G). Scale bars: 25 μm. [file 12983_2015_100_MOESM17_ESM.pdf]

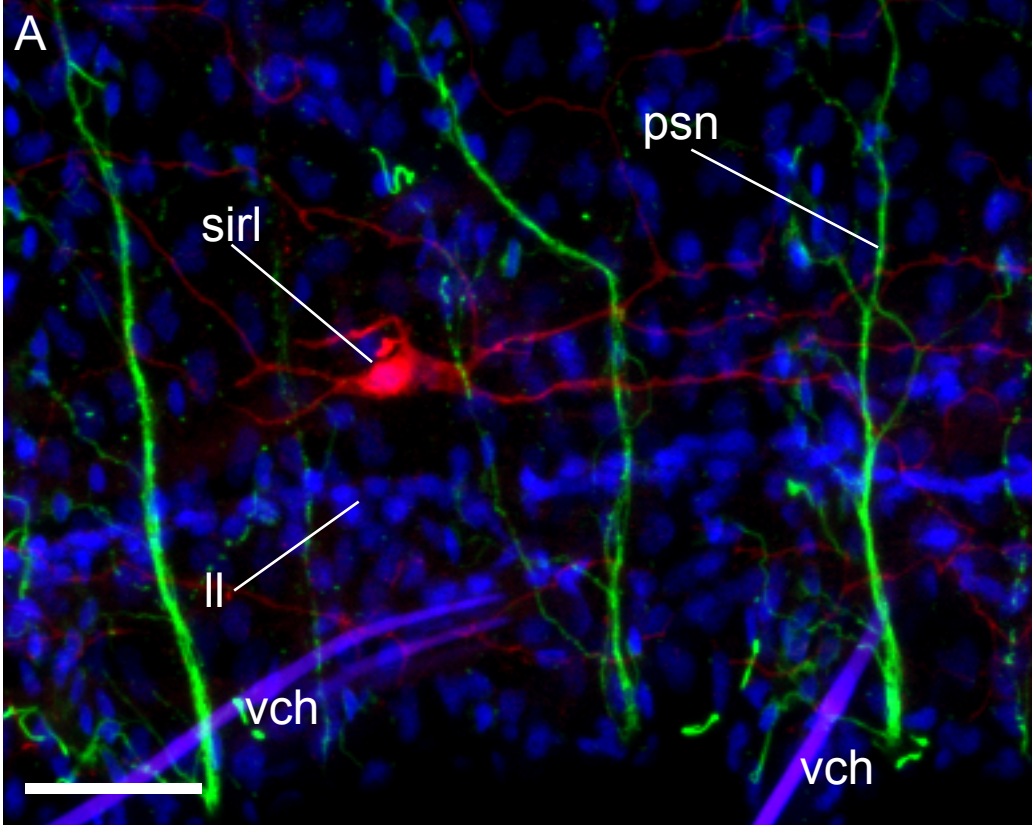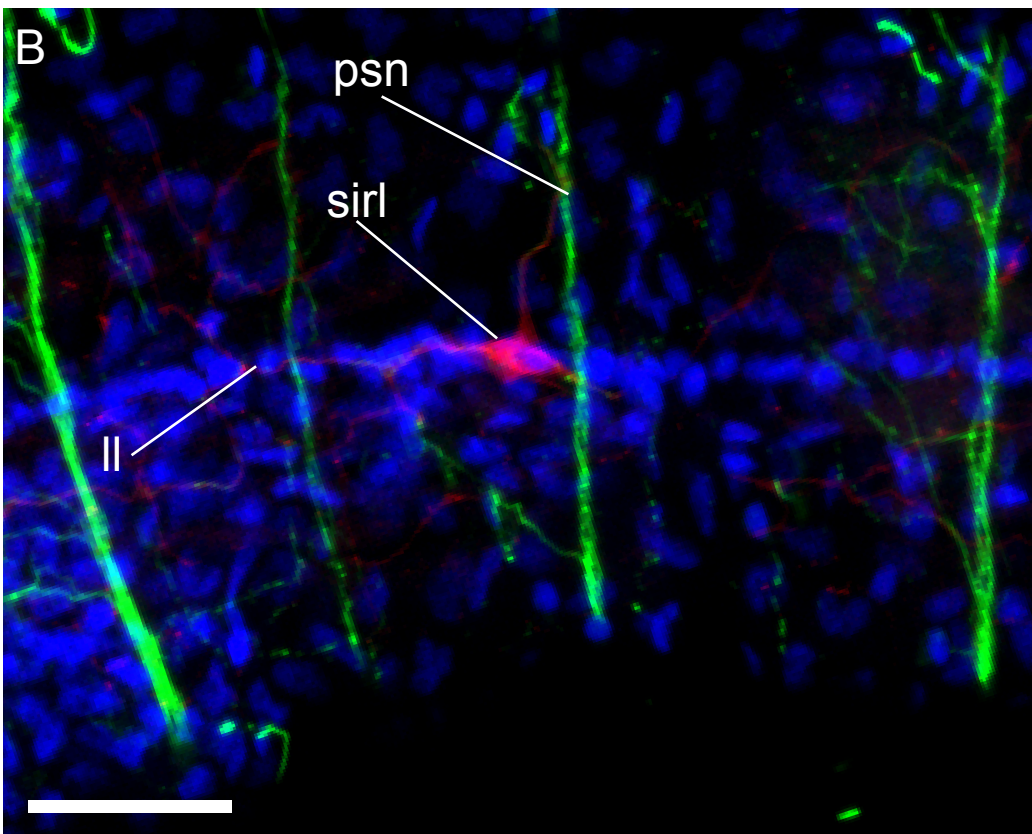

Supplement: Additional file 18: Figure S18. — Single serotonin-immunoreactive cells in the lateral body wall of Nais stolci. A,B) Representative images of two different segments from different individuals; maximum intensity projections of sagittal Z-stacks of representing thick optical sections level to the lateral body wall of mid-trunk segments. Specimens were stained for DNA (blue), serotonin (red) and acetyl-tubulin (green). ll: lateral line; psn: peripheral segmental nerve; sirl: serotonin-immunoreactive cell; vch: ventral chaetae. Scale bars: 25 μm. [file 12983_2015_100_MOESM18_ESM.pdf]
