# Supplementary material for: Fine taxonomic sampling of nervous systems within Naididae (Annelida: Clitellata) reveals evolutionary lability and revised homologies of annelid neural components
Source: Front Zool. 2015 Apr 18;12:8. doi: 10.1186/s12983-015-0100-6 (PMC4424535; doi:10.1186/s12983-015-0100-6)
Supplement: Additional file 19: — Supplementary results. [file 12983_2015_100_MOESM19_ESM.pdf]

Supplementary Information

Supplementary Information to the article:

Fine taxonomic sampling of nervous systems within Naididae (Annelida: Clitellata) reveals evolutionary lability and revised homologies of annelid neural components.

Eduardo E. Zattara & Alexandra E. Bely.

Frontiers in Zoology (DOI: 10.1186/s12983-015-0100-6)

Table of Contents

Supplementary Results 1
- Tubifex tubifex 2
- Pristina aequiseta 4
- Pristina leidyi 7
- Monopylephorus rubroniveus 10
- Dero digitata 12
- Dero furcata 14
- Allonais paraguayensis 17
- Paranais litoralis 19
- Amphichaeta sp. 22
- Chaetogaster diaphanus 24
- Nais stolci 27
- Stylaria lacustris 30

Supplementary Results

We characterized the morphology of the nervous system in 12 species of naidids using immunohistochemistry and confocal laser scanning microscopy. Detailed descriptions for each species follow below. We accompany these descriptions with diagrams of the nervous system of 10 out of 12

species (Fig. S1-S10; our staining and imaging of *Monopylephorus* and *Amphichaeta* failed to yield data of enough quality to support complete diagrams), an overview of a generalized naid body segment (Fig. S11) and image panels showing data for all species (Fig. S12-S18, plus data from Fig. 2, 3, 5 and 6). In our descriptions, we use whenever possible the terminology defined by Richter *et al.* [22]. While each species description stands alone, we use a parallel structure for each to facilitate comparisons across species. A summary of the character states for all neural traits we found to be variable can be found in Table 1.

Tubifex tubifex

The nervous system of *Tubifex tubifex* has three main components (Fig. S1): the anterior brain and associated peripheral nervous system, the ganglionated ventral nerve cord, and the segmental peripheral nerves.

Anterior nervous system

The brain is a paired bilobed structure located dorsal to the mouth; its anterior edge is within the prostomium; its posterior edge is approximately at the peristomium/first segment boundary (Fig. 3A, Fig. S13A). It is composed of an outer cell cortex surrounding an inner neuropil (Fig. 2C, Fig. S12A), and is linked to the ventral nerve cord by paired circumesophageal connectives, which also connect to paired sets of prostomial peripheral nerves (Fig. S14A). The anterior part of the brain neuropil has a network of serotonin immunoreactive (SIR) neurites, while acetyl-tubulin immunoreactive (acTIR) neurites form an arc across the posterior part (Fig. 2C, Fig. S12A). In the posterior lobe behind the neuropil, we found one or three pairs of SIR perikarya in recently hatched or older specimens, respectively. At the posterior edge of the brain is a set of acTIR hairs that are often associated with

muscle fibers attached to that point and to the dorsal body wall. A single, unpaired acTIR ciliary sense organ is located in front of the brain (Fig. 2C, Fig. S12A, Fig. S13A, Fig. S14A). The prostomium is cone-shaped (Fig. 3A, Fig. S13A); no eyes were detected. The acTIR tracts in the brain neuropil and the connectives arborize forward into a set of prostomial nerves that innervate a dense collection of epidermal acTIR sensory hair cells on the prostomial epidermis (Fig. S14A; see also diagram in Fig. 4).

Ventral nerve cord

The ventral nerve cord runs longitudinally down the length of the animal. There is one ganglion per segment, plus a subesophageal ganglion at the anterior end of the cord; the cell cortex is trough-shaped and a neuropil containing acTIR and SIR neurites runs through the trough. Along the ventral nerve cord, longitudinal acTIR neurites are found lateroventrally while longitudinal SIR neurites tend to be medial and dorsal; longitudinal nerve tracts are linked by segmentally iterated transverse commissures of variable acTIR neurite density (Fig. S15A, Fig. 6A).

In all ventral nerve cord ganglia a number of SIR perikarya are found connected to the longitudinal SIR nerve tracts (Fig. S1D, Fig. S15A). While the exact number and position varies both between and within individuals, we found usually 1-3 cells in the anterior third of the ganglion, 0-2 cells located laterally to the right or left of the middle third of the ganglion, 0-1 cell near the midline of the middle third of the ganglion, and 0 to several cells in the posterior third of the ganglion (see summary diagram in Fig. 5D). The distribution of these cells within a single ganglion is asymmetric, but consecutive ganglia show mirror symmetry, mostly due to alternating left or right location of the lateral cells in the middle third of the ganglion.

The anterior portion of the ventral nerve cord shows substantial differences from more posterior segments. The subesophageal ganglion and ganglia from segments 1-3 show a pattern of SIR perikarya

different from more posterior segments, with a larger number of cells that show bilaterally symmetric distribution (Fig. 6A). The subesophageal ganglion and ganglia from segments 1-4 have no obvious intervening connectives, thus forming an anterior medullary cord (Fig. 6D).

Starting at the boundary of segments 2 and 3, a muscular septum is found to separate the segmental coelomic compartments of the worm. In this species, each septum is located behind the posterior third of the ganglion, so that the whole ganglion is located within the coelomic compartment, a condition we denominate non-septate ganglia (Fig. 5B, 5D).

Peripheral segmental nerves

In each segment, four peripheral and segmentally iterated acTIR nerves branch off from the neuropil of the ventral nerve cord, exit the ganglia, pass ventrally through the body wall muscle layer and run subepidermally towards the segment's dorsum (Fig. S1C, S15A). Each nerve innervates a number of epidermal sensory hairs. Nerve II is the thickest, branching off the middle third of the ganglion and innervating hairs around the base of the chaetae; nerve IV and I are thinner, with nerve III being the thinnest (Fig. S1D, S15A). Nerve IV is associated with the septum. The pattern of segmental nerves differs in the anterior region of the worm, with segment 1-2 having 3 rather than 4 nerves (Fig. 6A; Fig. S1A-B).

Pristina aequiseta

The nervous system of *Pristina aequiseta* has three main components (Fig. S2): the anterior brain and associated peripheral nervous system, the ganglionated ventral nerve cord, and the segmental peripheral nerves.

Anterior nervous system

The brain is a paired bilobed structure located dorsal to the mouth; its anterior edge is within the prostomium; its posterior edge is approximately at the peristomium/first segment boundary (Fig. S13B). It is composed of an outer cell cortex surrounding an inner neuropil (Fig. S12B), and is linked to the ventral nerve cord by paired circumesophageal connectives, which also connect to paired sets of prostomial peripheral nerves (Fig. S14B). The anterior part of the brain neuropil has a network of serotonin immunoreactive (SIR) neurites, while acetyl-tubulin immunoreactive (acTIR) neurites form an arc across the posterior part (Fig. S12B). In the posterior lobe behind the neuropil is a single pair of SIR perikarya. At the posterior edge of the brain is a set of acTIR hairs that are often associated with muscle fibers attached to that point and to the dorsal body wall. Two acTIR ciliary sense organs are located in front of the brain (Fig. S12B, Fig. S13B, Fig. S14B). The prostomium is cone-shaped and its anterodorsal tip elongates into a trunk-like proboscis (Fig. S12B, Fig. S13B, Fig. S14B); no eyes were detected. The acTIR tracts in the brain neuropil and the connectives arborize forward into a set of prostomial nerves that innervate a dense collection of epidermal acTIR sensory hair cells on the prostomial epidermis (Fig. S14B; see also diagram in Fig. 4); one pair of nerves (denominated D in Fig. 4) extends the length of the proboscis.

Ventral nerve cord

The ventral nerve cord runs longitudinally down the length of the animal. There is one ganglion per segment, plus a subesophageal ganglion at the anterior end of the cord; the cell cortex is trough-shaped and a neuropil containing acTIR and SIR neurites runs through the trough. Along the ventral nerve cord, longitudinal acTIR neurites are found lateroventrally while longitudinal SIR neurites tend to

be medial and dorsal; longitudinal nerve tracts are linked by segmentally iterated transverse commissures of variable acTIR neurite density (Fig S15B, Fig. 6B).

In all ventral nerve cord ganglia a number of SIR perikarya are found connected to the longitudinal SIR nerve tracts (Fig. S2D, Fig S15B). While the exact number and position varies both between and within individuals, we found usually 0-2 cells in the anterior third of the ganglion, 1 cell located laterally to the right or left of the middle third of the ganglion, 0-2 cells near the midline of the middle third of the ganglion, and 0-1 cell at the posterior third of the ganglion (see summary diagram in Fig. 5D). The distribution of these cells within a single ganglion is asymmetric, but consecutive ganglia show mirror symmetry, mostly due to alternating left or right location of the lateral cells at the middle third of the ganglion.

The anterior portion of the ventral nerve cord shows substantial differences from more posterior segments. The subesophageal ganglion and ganglia from segments 1-4 show a different pattern of SIR perikarya, with a larger number of cells that show bilaterally symmetric distribution (Fig. 6B). The subesophageal ganglion and ganglia from segments 1-4 have no obvious intervening connectives, thus forming an anterior medullary cord (Fig. S17A).

Starting at the boundary of segments 2 and 3, a muscular septum is found to separate the segmental coelomic compartments of the worm. In this species, each septum is located in front of the posterior third of the ganglion, so that the anterior two-thirds of the ganglion are located within the coelomic compartment of its corresponding segment, while the posterior third is located in the compartment of the following segment, a condition we denominate septate ganglia (Fig. 5B, 5D).

Peripheral segmental nerves

In each segment, four peripheral and segmentally iterated acTIR nerves branch off from the neuropil of the ventral nerve cord, exit the ganglia, pass ventrally through the body wall muscle layer and run subepidermally towards the segment's dorsum (Fig. S2C, S15B). Each nerve innervates a number of epidermal sensory hairs. Nerve II is the thickest, branching off the middle third of the ganglion and innervating hairs around the base of the chaetae; nerve IV and I are thinner (Fig. S2D, S15B). Nerve III is very small and sometimes not detectable or absent, and is associated with the septum. The pattern of segmental nerves differs in the anterior region of the worm, with segment 1-3 showing 2 nerves and segment 3-4 having 3 nerves (Fig. 6B; Fig. S2A-B).

Pristina leidyi

The nervous system of *Pristina leidyi* has three main components (Fig. S3): the anterior brain and associated peripheral nervous system, the ganglionated ventral nerve cord, and the segmental peripheral nerves.

Anterior nervous system

The brain is a paired bilobed structure located dorsal to the mouth; its anterior edge is within the prostomium; its posterior edge is approximately at the peristomium/first segment boundary (Fig. S13C). It is composed of an outer cell cortex surrounding an inner neuropil (Fig. 2D, Fig. S12C), and is linked to the ventral nerve cord by paired circumesophageal connectives, which also connect to paired sets of prostomial peripheral nerves (Fig. S14C). The anterior part of the brain neuropil has a network of serotonin immunoreactive (SIR) neurites, while acetyl-tubulin immunoreactive (acTIR) neurites form an

arc across the posterior part (Fig. S12C). In the posterior lobe behind the neuropil is a single pair of SIR perikarya. At the posterior edge of the brain is a set of acTIR hairs that are often associated with muscle fibers attached to that point and to the dorsal body wall. Two acTIR ciliary sense organs are located in front of the brain (Fig. S12C, Fig. S13C, Fig. S14C).The prostomium is cone-shaped and its anterodorsal tip elongates into a trunk-like proboscis (Fig. S12C, Fig. S13C, Fig. S14C); no eyes were detected. The acTIR tracts in the brain neuropil and the connectives arborize forward into a set of prostomial nerves that innervate a dense collection of epidermal acTIR sensory hair cells on the prostomial epidermis (Fig. S14C; see also diagram in Fig. 4); one pair of nerves (denominated D in Fig. 4) extends the length of the proboscis.

Ventral nerve cord

The ventral nerve cord runs longitudinally down the length of the animal. There is one ganglion per segment, plus a subesophageal ganglion at the anterior end of the cord; the cell cortex is trough-shaped and a neuropil containing acTIR and SIR neurites runs through the trough. Along the ventral nerve cord, longitudinal acTIR neurites are found lateroventrally while longitudinal SIR neurites tend to be medial and dorsal; longitudinal nerve tracts are linked by segmentally iterated transverse commissures of variable acTIR neurite density (Fig S15C, Fig. S16A).

In all ventral nerve cord ganglia a number of SIR perikarya are found connected to the longitudinal SIR nerve tracts (Fig. S3D, Fig S15C). While the exact number and position varies both between and within individuals, we found usually 1-2 cells in the anterior third of the ganglion, 1 cell located laterally to the right or left of the middle third of the ganglion, 1-2 cells near the midline of the middle third of the ganglion, and 0-1 cell at the posterior third of the ganglion (see summary diagram in Fig. 5D). The distribution of these cells within a single ganglion is asymmetric, but consecutive ganglia

show mirror symmetry, mostly due to alternating left or right location of the lateral cells at the middle third of the ganglion.

The anterior portion of the ventral nerve cord shows substantial differences from more posterior segments. The subesophageal ganglion and ganglia from segments 1-4 show a different pattern of SIR perikarya, with a larger number of cells that show bilaterally symmetric distribution (Fig. S16A). The subesophageal ganglion and ganglia from segments 1-4 have no obvious intervening connectives, thus forming an anterior medullary cord (Fig. 6F).

Starting at the boundary of segments 2 and 3, a muscular septum is found to separate the segmental coelomic compartments of the worm. In this species, each septum is located in front of the posterior third of the ganglion, so that the anterior two-thirds of the ganglion are located within the coelomic compartment of its corresponding segment, while the posterior third is located in the compartment of the following segment, a condition we denominate septate ganglia (Fig. 5B, 5D).

Peripheral segmental nerves

In each segment, four peripheral and segmentally iterated acTIR nerves branch off from the neuropil of the ventral nerve cord, exit the ganglia, pass ventrally through the body wall muscle layer and run subepidermally towards the segment's dorsum (Fig. S3C, S15C). Each nerve innervates a number of epidermal sensory hairs. Nerve II is the thickest, branching off the middle third of the ganglion and innervating hairs around the base of the chaetae; nerve IV and I are thinner (Fig. S3D, S15C). Nerve III is very small and usually not detectable or absent; when present, is associated with the septum. The pattern of segmental nerves differs in the anterior region of the worm, with segments 1-4 showing 2 (Fig. S16A; Fig. S3A-B).

Monopylephorus rubroniveus

The nervous system of *Monopylephorus rubroniveus* has three main components: the anterior brain and associated peripheral nervous system, the ganglionated ventral nerve cord, and the segmental peripheral nerves.

Anterior nervous system

The brain is a paired bilobed structure located posterior and dorsal to the mouth; its anterior edge is level with the chaetae of segment 1; its posterior edge is approximately at the anterior half of segment 2 (Fig. S13D). It includes an inner neuropil that is linked to the ventral nerve cord by paired circumesophageal connectives, which also connect to paired sets of prostomial peripheral nerves (Fig. S13D, Fig. S14D). The specimens we had available stained poorly and did not allow for detailed analysis of the brain structure, but we found evidence in the posterior brain lobe for a single pair of SIR perikarya (Fig. S12D). We found no evidence of ciliary sense organs. The prostomium is blunt (Fig. S13D, Fig. S14D); no eyes were detected. The acTIR tracts in the brain neuropil and the connectives arborize forward into a set of prostomial nerves that innervate a dense collection of epidermal acTIR sensory hair cells on the prostomial epidermis (Fig. S14D).

Ventral nerve cord

The ventral nerve cord runs longitudinally down the length of the animal. There is one ganglion per segment, plus a subesophageal ganglion at the anterior end of the cord. Along the ventral nerve cord, longitudinal acTIR neurites are found lateroventrally while longitudinal SIR neurites tend to be medial and dorsal; longitudinal nerve tracts are linked by segmentally iterated transverse commissures of variable acTIR neurite density (Fig. S16B).

In all ventral nerve cord ganglia a number of SIR perikarya are found connected to the longitudinal SIR nerve tracts (Fig. S16B). While the exact number and position varies both between and within individuals, we found usually medial 1-2 cells and 0-2 lateral cells in the anterior third of the ganglion, 1 cell located laterally to the right or left of the middle third of the ganglion, sometimes with a more weakly staining contralateral cell, 1-2 cells near the midline of the middle third of the ganglion, and 1-2 cells in the posterior third of the ganglion (see summary diagram in Fig. 5D). The distribution of these cells within a single ganglion is asymmetric, but consecutive ganglia show mirror symmetry, mostly due to alternating left or right location of the more intense staining lateral cells at the anterior and middle third of the ganglion.

The anterior portion of the ventral nerve cord shows substantial differences from more posterior segments. The subesophageal ganglion and ganglia from segments 1-4 show a different pattern of SIR perikarya, with a larger number of cells that show bilaterally symmetric distribution (Fig. S16B). Due to poor nuclear and acetyl-tubulin staining, we could not verify the presence of an anterior medullary cord as seen in other species.

Starting at the boundary of segments 3 and 4, a muscular septum is found to separate the segmental coelomic compartments of the worm. While deficient nuclear staining kept us from delineating the boundaries of the ganglion, careful observation using SIR perikarya and peripheral nerves supports that each septum is located behind the posterior third of the ganglion, so that the whole ganglion is located within the coelomic compartment: thus, this species is likely to have non-septate ganglia (Fig. 5B, 5D).

Peripheral segmental nerves

Our specimens showed extensive, non-neural acetyl-tubulin staining in the body wall, which significantly masked peripheral segmental nerves. However, by following the nerve roots as they exit from the ventral ganglion we were able to detect four peripheral nerves per segment (Fig. 5D). In contrast to other naidid species, we found this pattern in all segments, including the anterior ones.

Dero digitata

The nervous system of *Dero digitata* has three main components (Fig. S4): the anterior brain and associated peripheral nervous system, the ganglionated ventral nerve cord, and the segmental peripheral nerves.

Anterior nervous system

The brain is a paired bilobed structure located dorsal to the mouth; its anterior edge is within the peristomium; its posterior edge is at middle of segment 1 (Fig. S13E). It is composed of an outer cell cortex surrounding an inner neuropil (Fig. S12E), and is linked to the ventral nerve cord by paired circumesophageal connectives, which also connect to paired sets of prostomial peripheral nerves (Fig. S14E). The anterior part of the brain neuropil has a network of serotonin immunoreactive (SIR) neurites, while acetyl-tubulin immunoreactive (acTIR) neurites form an arc across the posterior part (Fig. S12E). In the posterior lobe behind the neuropil there are two pairs of SIR perikarya. At the posterior edge of the brain is a set of acTIR hairs that are often associated with muscle fibers attached to that point and to the dorsal body wall. Two acTIR ciliary sense organs are located in front of the brain (Fig. S12E, Fig. S13E, Fig. S14E). The prostomium is cone-shaped (Fig. S13E); no eyes were detected.

The acTIR tracts in the brain neuropil and the connectives arborize forward into a set of prostomial nerves that innervate a dense collection of epidermal acTIR sensory hair cells on the prostomial epidermis (Fig. S14E; see also diagram in Fig. 4).

Ventral nerve cord

The ventral nerve cord runs longitudinally down the length of the animal. There is one ganglion per segment, plus a subesophageal ganglion at the anterior end of the cord; the cell cortex is trough-shaped and a neuropil containing acTIR and SIR neurites runs through the trough. Along the ventral nerve cord, longitudinal acTIR neurites are found lateroventrally while longitudinal SIR neurites tend to be medial and dorsal; longitudinal nerve tracts are linked by segmentally iterated transverse commissures of variable acTIR neurite density (Fig S15D).

In all ventral nerve cord ganglia a number of SIR perikarya are found connected to the longitudinal SIR nerve tracts (Fig. S4D, Fig S15D). While the exact number and position varies both between and within individuals, we found usually 2-4 cells in the anterior third of the ganglion, 1 cell located laterally to the right or left of the middle third of the ganglion, 1-3 cells near the midline of the middle third of the ganglion, and 1 cell at the posterior third of the ganglion (see summary diagram in Fig. 5D). The distribution of these cells within a single ganglion is asymmetric, but consecutive ganglia show mirror symmetry, mostly due to alternating left or right location of the lateral cells at the middle third of the ganglion.

The anterior portion of the ventral nerve cord shows substantial differences from more posterior segments. The subesophageal ganglion and ganglia from segments 1-4 show a pattern of SIR perikarya different from more posterior segments, with a larger number of cells that show bilaterally symmetric

distribution (Fig. S16C). The subesophageal ganglion and ganglia from segments 1-5 have no obvious intervening connectives, thus forming an anterior medullary cord (Fig. S17B).

Starting at the boundary of segments 3 and 4, a muscular septum is found to separate the segmental coelomic compartments of the worm. In this species, each septum is located behind the posterior third of the ganglion, so that the whole ganglion is located within the coelomic compartment, a condition we denominate non-septate ganglia (Fig. 5B, 5D).

Peripheral segmental nerves

In each segment, four peripheral and segmentally iterated acTIR nerves branch off from the neuropil of the ventral nerve cord, exit the ganglia, pass ventrally through the body wall muscle layer and run subepidermally towards the segment's dorsum (Fig. S4C, S15D). Each nerve innervates a number of epidermal sensory hairs. Nerves I, II and IV are similar in thickness, while nerve III is much thinner; nerve II innervates hairs around the base of the chaetae (Fig. S4D, S15D). Nerve IV is associated with the septum. The pattern of segmental nerves differs in the anterior region of the worm, with segment 1-4 showing 3 rather than 4 nerves (Fig. S16C; Fig S4A-B).

Dero furcata

The nervous system of *Dero furcata* has three main components (Fig. S5): the anterior brain and associated peripheral nervous system, the ganglionated ventral nerve cord, and the segmental peripheral nerves.

Anterior nervous system

The brain is a paired bilobed structure located dorsal to the mouth; its anterior edge is at the boundary between prostomium and peristomium; its posterior edge is at middle of segment 1 (Fig. S13F). It is composed of an outer cell cortex surrounding an inner neuropil (Fig. S12F), and is linked to the ventral nerve cord by paired circumesophageal connectives, which also connect to paired sets of prostomial peripheral nerves (Fig. S14F). The anterior part of the brain neuropil has a network of serotonin immunoreactive (SIR) neurites, while acetyl-tubulin immunoreactive (acTIR) neurites form an arc across the posterior part (Fig. S12F). In the posterior lobe behind the neuropil there are two or three pairs of SIR perikarya. At the posterior edge of the brain is a set of acTIR hairs that are often associated with muscle fibers attached to that point and to the dorsal body wall. Two acTIR ciliary sense organs are located in front of the brain (Fig. S12F, Fig. S13F, Fig. S14F). The prostomium is cone-shaped (Fig. S13F); no eyes were detected. The acTIR tracts in the brain neuropil and the connectives arborize forward into a set of prostomial nerves that innervate a dense collection of epidermal acTIR sensory hair cells on the prostomial epidermis (Fig. S14F; see also diagram in Fig. 4).

Ventral nerve cord

The ventral nerve cord runs longitudinally down the length of the animal. There is one ganglion per segment, plus a subesophageal ganglion at the anterior end of the cord; the cell cortex is trough-shaped and a neuropil containing acTIR and SIR neurites runs through the trough. Along the ventral nerve cord, longitudinal acTIR neurites are found lateroventrally while longitudinal SIR neurites tend to be medial and dorsal; longitudinal nerve tracts are linked by segmentally iterated transverse commissures of variable acTIR neurite density (Fig S15E).

In all ventral nerve cord ganglia a number of SIR perikarya are found connected to the longitudinal SIR nerve tracts (Fig. S5D, Fig S15E). While the exact number and position varies both between and within individuals, we found usually 3-4 cells in the anterior third of the ganglion, 1 cell located laterally to the right or left of the middle third of the ganglion, 2 cells near the midline of the middle third of the ganglion, and 1 cell at the posterior third of the ganglion (see summary diagram in Fig. 5D). The distribution of these cells within a single ganglion is asymmetric, but consecutive ganglia show mirror symmetry, mostly due to alternating left or right location of the lateral cells at the middle third of the ganglion.

The anterior portion of the ventral nerve cord shows substantial differences from more posterior segments. The subesophageal ganglion and ganglia from segments 1-4 show a pattern of SIR perikarya different from more posterior segments, with a larger number of cells that show bilaterally symmetric distribution (Fig. S16D). The subesophageal ganglion and ganglia from segments 1-4 have no obvious intervening connectives, thus forming an anterior medullary cord (Fig. S17C).

Starting at the boundary of segments 3 and 4, a muscular septum is found to separate the segmental coelomic compartments of the worm. In this species, each septum is located in front of the posterior third of the ganglion, so that the anterior two-thirds of the ganglion are located within the coelomic compartment of its corresponding segment, while the posterior third is located in the compartment of the following segment, a condition we denominate septate ganglia (Fig. 5B, 5D).

Peripheral segmental nerves

In each segment, four peripheral and segmentally iterated acTIR nerves branch off from the neuropil of the ventral nerve cord, exit the ganglia, pass ventrally through the body wall muscle layer and run subepidermally towards the segment's dorsum (Fig. S5C, S15E). Each nerve innervates a

number of epidermal sensory hairs. Nerves I, II and IV are similar in thickness, while nerve III is much thinner; nerve II innervates hairs around the base of the chaetae (Fig. S5D, S15E). Nerve III is associated with the septum. The pattern of segmental nerves differs in the anterior region of the worm, with segment 1-4 showing 2-3 rather than 4 nerves (Fig. S16D; Fig S5A-B).

Allonais paraguayensis

The nervous system of *Allonais paraguayensis* has three main components (Fig. S6): the anterior brain and associated peripheral nervous system, the ganglionated ventral nerve cord, and the segmental peripheral nerves.

Anterior nervous system

The brain is a paired bilobed structure located dorsal to the mouth; its anterior edge is within the peristomium; its posterior edge is at middle of segment 1 (Fig. 3B, Fig. S13G). It is composed of an outer cell cortex surrounding an inner neuropil (Fig. S12G), and is linked to the ventral nerve cord by paired circumesophageal connectives, which also connect to paired sets of prostomial peripheral nerves (Fig. S14G). The anterior part of the brain neuropil has a network of serotonin immunoreactive (SIR) neurites, while acetyl-tubulin immunoreactive (acTIR) neurites form an arc across the posterior part (Fig. S12G). In the posterior lobe behind the neuropil there is one pair of SIR perikarya. At the posterior edge of the brain is a set of acTIR hairs that are often associated with muscle fibers attached to that point and to the dorsal body wall. Two acTIR ciliary sense organs are located in front of the brain (Fig. S12G, Fig. S13G, Fig. S14G). The prostomium is cone-shaped (Fig. 3B , Fig. S13G); no eyes were detected. The acTIR tracts in the brain neuropil and the connectives arborize forward into a set of prostomial

nerves that innervate a dense collection of epidermal acTIR sensory hair cells on the prostomial epidermis (Fig. S14G; see also diagram in Fig. 4).

Ventral nerve cord

The ventral nerve cord runs longitudinally down the length of the animal. There is one ganglion per segment, plus a subesophageal ganglion at the anterior end of the cord; the cell cortex is trough-shaped and a neuropil containing acTIR and SIR neurites runs through the trough. Along the ventral nerve cord, longitudinal acTIR neurites are found lateroventrally while longitudinal SIR neurites tend to be medial and dorsal; longitudinal nerve tracts are linked by segmentally iterated transverse commissures of variable acTIR neurite density (Fig 5A).

In all ventral nerve cord ganglia a number of SIR perikarya are found connected to the longitudinal SIR nerve tracts (Fig. S6D, Fig 5A). While the exact number and position varies both between and within individuals, we found usually 2-6 cells in the anterior third of the ganglion (sometimes close to the middle third), 1-2 cells located laterally to the right or left of the middle third of the ganglion, 1-2 cells near the midline of the middle third of the ganglion, and 1 cell at the posterior third of the ganglion (see summary diagram in Fig. 5D). The distribution of these cells within a single ganglion is asymmetric, but consecutive ganglia show mirror symmetry, mostly due to alternating left or right location of the lateral cells at the middle third of the ganglion.

The anterior portion of the ventral nerve cord shows substantial differences from more posterior segments. The subesophageal ganglion and ganglia from segments 1-4 show a pattern of SIR perikarya different from more posterior segments, with a larger number of cells that show bilaterally symmetric distribution (Fig. 6C). The subesophageal ganglion and ganglia from segments 1-5 have no obvious intervening connectives, thus forming an anterior medullary cord (Fig. 6G).

Starting at the boundary of segments 3 and 4, a muscular septum is found to separate the segmental coelomic compartments of the worm. In this species, each septum is located behind the posterior third of the ganglion, so that the whole ganglion is located within the coelomic compartment, a condition we denominate non-septate ganglia (Fig. 5B, 5D).

Peripheral segmental nerves

In each segment, four peripheral and segmentally iterated acTIR nerves branch off from the neuropil of the ventral nerve cord, exit the ganglia, pass ventrally through the body wall muscle layer and run subepidermally towards the segment's dorsum (Fig. 5A). Each nerve innervates a number of epidermal sensory hairs. Nerves I, II and IV are more or less similar in thickness, while nerve III is reduced and sometimes absent; nerve II innervates hairs around the base of the chaetae (Fig. 5A). Nerve IV is associated with the septum. The pattern of segmental nerves differs in the anterior region of the worm, with segment 1-4 showing 2 rather than 4 nerves (Fig. 6C).

Paranais litoralis

The nervous system of *Paranais litoralis* has three main components (Fig. S7): the anterior brain and associated peripheral nervous system, the ganglionated ventral nerve cord, and the segmental peripheral nerves.

Anterior nervous system

The brain is a paired bilobed structure located posterior to the mouth and anterodorsal to the pharyngeal sac; its anterior edge is level with the chaetae of segment 1; its posterior edge is level with the chaetae of segment 2 (Fig. 3D, Fig. S13H). It is composed of an outer cell cortex surrounding an

inner neuropil (Fig. 2H, Fig. S12H), and is linked to the ventral nerve cord by paired circumesophageal connectives, which also connect to paired sets of prostomial peripheral nerves (Fig. S14H). The anterior part of the brain neuropil has a network of serotonin immunoreactive (SIR) neurites, while acetyl-tubulin immunoreactive (acTIR) neurites form an arc across the posterior part (Fig. 2H, Fig. S12H). In the posterior lobe behind the neuropil there are five pairs of SIR perikarya. At the posterior edge of the brain is a set of acTIR hairs that are often associated with muscle fibers attached to that point and to the dorsal body wall. Two acTIR ciliary sense organs are located in the prostomium, well anterior to the brain and connected to it by acTIR neurites (Fig. S12H, Fig. S13H, Fig. S14H). The prostomium is small and blunt shaped (Fig. 3D, Fig. S13H); no eyes were detected. The acTIR tracts in the brain neuropil and the connectives arborize forward into a set of prostomial nerves that innervate a dense collection of epidermal acTIR sensory hair cells on the prostomial epidermis (Fig. S14H; see also diagram in Fig. 4).

Ventral nerve cord

The ventral nerve cord runs longitudinally down the length of the animal. There is one ganglion per segment, plus a subesophageal ganglion at the anterior end of the cord; the cell cortex is trough-shaped and a neuropil containing acTIR and SIR neurites runs through the trough. Along the ventral nerve cord, longitudinal acTIR neurites are found lateroventrally while longitudinal SIR neurites tend to be medial and dorsal; longitudinal nerve tracts are linked by segmentally iterated transverse commissures of variable acTIR neurite density (Fig S15F).

In all ventral nerve cord ganglia a number of SIR perikarya are found connected to the longitudinal SIR nerve tracts (Fig. S7D, Fig S15F). While the exact number and position varies both between and within individuals, we found usually 1 cell at the anterior third of the ganglion (usually

close to the middle third), 1-2 cells located laterally to the right or left of the middle third of the ganglion, 0-2 cells near the midline of the middle third of the ganglion, and 1 cell at or close to the posterior edge of the middle third of the ganglion (see summary diagram in Fig. 5D). The distribution of these cells within a single ganglion is asymmetric, but consecutive ganglia show mirror symmetry, mostly due to alternating left or right location of the lateral cells at the middle third of the ganglion.

The anterior portion of the ventral nerve cord shows substantial differences from more posterior segments. The subesophageal ganglion and ganglia from segments 1-4 show a pattern of SIR perikarya different from more posterior segments, with a larger number of cells that show bilaterally symmetric distribution (Fig. S16E). The subesophageal ganglion and ganglia from segments 1-3 have no obvious intervening connectives, thus forming an anterior medullary cord (Fig. 17D).

Starting at the boundary of segments 3 and 4, a muscular septum is found to separate the segmental coelomic compartments of the worm. In this species, each septum is located in front of the posterior third of the ganglion, so that the anterior two-thirds of the ganglion are located within the coelomic compartment of its corresponding segment, while the posterior third is located in the compartment of the following segment, a condition we denominate septate ganglia (Fig. 5B, 5D).

Peripheral segmental nerves

In each segment, four peripheral and segmentally iterated acTIR nerves branch off from the neuropil of the ventral nerve cord, exit the ganglia, pass ventrally through the body wall muscle layer and run subepidermally towards the segment's dorsum (Fig. S15F). Each nerve innervates a number of epidermal sensory hairs. Nerves II and IV are thicker. And nerves I and III are thinner; nerve II innervates hairs around the base of the chaetae (Fig. S15F). Nerve III is associated with the septum. The

pattern of segmental nerves differs in the anterior region of the worm, with segment 1 showing 3 rather than 4 nerves (Fig. S16E).

Amphichaeta sp.

The nervous system of *Amphichaeta* sp. has three main components: the anterior brain and associated peripheral nervous system, the ganglionated ventral nerve cord, and the segmental peripheral nerves.

Anterior nervous system

The brain is a paired bilobed structure located dorsal to the mouth; its anterior edge is at the posterior part of the prostomium; its posterior edge is located within the peristomium, and is continuous with paired medullary nerve cords that innervate the pharyngeal sac (Fig. S12I, Fig. S13I). It is composed of an outer cell cortex surrounding an inner neuropil (Fig. S12I), and is linked to the ventral nerve cord by paired circumesophageal connectives, which also connect to paired sets of prostomial peripheral nerves (Fig. S14I). The outer cortex is in contact with the inner side of the body wall. The central part of the brain neuropil has a network of serotonin immunoreactive (SIR) neurites, while acetyl-tubulin immunoreactive (acTIR) neurites form both a median connective and an arc across the posterior part (Fig. S12I). In the posterior lobe behind the neuropil there is a pair of SIR perikarya. The acTIR hairs that are conspicuous in other species were not obvious in our specimens. Two acTIR ciliary sense organs are located in the prostomium, somewhat anterior to the brain; they are formed by a looser coil than what observed in most other species (Fig. S12I, Fig. S13I, Fig. S14I). The prostomium is small and shaped as a pointed cone (Fig. S13I); no eyes were detected. The acTIR tracts in the brain neuropil

and the connectives arborize forward into a set of short prostomial nerves that innervate a dense collection of epidermal acTIR sensory hair cells on the prostomial epidermis (Fig. S14I).

Ventral nerve cord

The ventral nerve cord runs longitudinally down the length of the animal. There is one ganglion per segment, plus a subesophageal ganglion at the anterior end of the cord; the cell cortex is trough-shaped and a neuropil containing acTIR and SIR neurites runs through the trough. The distribution of acTIR and SIR neurites within the ventral nerve cord was qualitatively different to that seen in other species, with fine SIR neurites distributed within a “matrix” of acTIR neuropil showing no clear axonal structure (Fig. S15G). We lack enough independently processed specimens to tell whether this difference is an actual unique trait of the species, or a technical staining artifact.

In all ventral nerve cord ganglia a number of SIR perikarya are found connected to the longitudinal SIR nerve tracts (Fig S15G). We found 2 weakly staining cells in the anterior third of the ganglion, 1 cell located laterally to the right or left of the middle third of the ganglion, and 1 cell located in the posterior third of the ganglion (see summary diagram in Fig. 5D). The distribution of these cells within a single ganglion is asymmetric, but consecutive ganglia show mirror symmetry, mostly due to alternating left or right location of the lateral cells at the middle third of the ganglion.

The anterior portion of the ventral nerve cord shows substantial differences from more posterior segments. The subesophageal ganglion and ganglia from segments 1-4 show a pattern of SIR perikarya different from more posterior segments, with a larger number of cells that show bilaterally symmetric distribution (Fig. S16F). The subesophageal ganglion and ganglia from segments 1-2 have no obvious intervening connectives, thus forming an anterior medullary cord (Fig. 6E). Furthermore, this anterior

region of the ventral nerve cord is partially separated by muscle bundles that separate the neuropil into two parallel tracts (Fig. S16F).

Starting at the boundary of segments 2 and 3, a muscular septum is found to separate the segmental coelomic compartments of the worm. In this species, each septum is located behind the posterior third of the ganglion, so that the whole ganglion is located within the coelomic compartment, a condition we denominate non-septate ganglia (Fig. 5B, 5D).

Peripheral segmental nerves

In each segment, four peripheral and segmentally iterated acTIR nerves branch off from the neuropil of the ventral nerve cord, exit the ganglia, pass ventrally through the body wall muscle layer and run subepidermally towards the segment's dorsum (Fig. S15G). Each nerve innervates a number of epidermal sensory hairs. Interestingly, and in contrast with all other species described in this study except *Chaetogaster*, it is the third nerve the one that innervates hairs around the base of the chaetae (in contrast with nerve II in most other species). The fourth nerve is associated with the septum. The pattern of segmental nerves differs in the anterior region of the worm, with segment 1 showing 2 nerves and segment 2 showing 3 nerves (Fig. S16F).

Chaetogaster diaphanus

The nervous system of *Chaetogaster diaphanus* has three main components (Fig. S8): the anterior brain and associated peripheral nervous system, the ganglionated ventral nerve cord, and the segmental peripheral nerves.

Anterior nervous system

The brain is a paired bilobed structure located dorsally, posterior to the mouth; both its anterior and posterior edges are located within segment 1 (Fig. 3C, Fig S13J). It is composed of an outer cell cortex surrounding an inner neuropil (Fig. S12J), and is linked to the ventral nerve cord by paired circumesophageal connectives, which also connect to paired sets of prostomial peripheral nerves (Fig. S14J). The central part of the brain neuropil has a network of serotonin immunoreactive (SIR) neurites, while acetyl-tubulin immunoreactive (acTIR) neurites form an arc across the posterior part (Fig. S12J). We found no SIR perikarya associated to the brain. The acTIR hairs that are conspicuous in other species were not obvious in our specimens. Four or six acTIR ciliary sense organs are located in front of the brain or projecting forward and connected by acTIR neurites (Fig. 2F, Fig. S12J, Fig. S13J, Fig. S14J). The prostomium is much reduced and divided in two “lips” lining a very large mouth (Fig. S12J, Fig. S13J); no eyes were detected. The acTIR tracts in the brain neuropil and the connectives arborize forward into a set of prostomial nerves that innervate a dense collection of epidermal acTIR sensory hair cells on the prostomial epidermis (Fig. S14J).

Ventral nerve cord

The ventral nerve cord runs longitudinally down the length of the animal. There is one ganglion per segment, plus a subesophageal ganglion at the anterior end of the cord; the cell cortex is trough-shaped and a neuropil containing acTIR and SIR neurites runs through the trough. Along the ventral nerve cord, longitudinal acTIR neurites are found lateroventrally while longitudinal SIR neurites tend to be medial and dorsal; longitudinal nerve tracts are linked by segmentally iterated transverse commissures of variable acTIR neurite density (Fig S15H).

In all ventral nerve cord ganglia a number of SIR perikarya are found connected to the longitudinal SIR nerve tracts (Fig S15H). We found 1-2 weakly staining cells in the anterior third of the ganglion, 1 cell located laterally to the right or left of the middle third of the ganglion, 1 cell lateral to the midline within the middle third of the ganglion and 1 cell located in the posterior third of the ganglion (see summary diagram in Fig. 5D). The distribution of these cells within a single ganglion is asymmetric, but consecutive ganglia show mirror symmetry, mostly due to alternating left or right location of the lateral cells at the middle third of the ganglion.

The anterior portion of the ventral nerve cord shows substantial differences from more posterior segments. The subesophageal ganglion and ganglia from segments 1-3 show a pattern of SIR perikarya different from more posterior segments, with a larger number of cells that show bilaterally symmetric distribution (Fig. S16G). The subesophageal ganglion and ganglia from segments 1-2 have no obvious intervening connectives, thus forming an anterior medullary cord (Fig. S17E). Furthermore, at this anterior region the ventral nerve cord separates into paired parallel hemiganglia (Fig. S16G).

The whole anterior region of the body of this species is considerably different from other species described here, mostly due to a very large, muscular pharynx that occupies almost completely segments 1 and 2, and one half of segment 3. A muscular septum divides segment 3 in two and separates the anterior third of its ganglion from the posterior two thirds. The next septum is located between segments 4 and 5, and this and all following septa are located behind the posterior third of the ganglion, so that the whole ganglion is located within the coelomic compartment, a condition we denominate non-septate ganglia (Fig. 5B, 5D).

Peripheral segmental nerves

In each segment, five peripheral and segmentally iterated acTIR nerves branch off from the neuropil of the ventral nerve cord, exit the ganglia, pass ventrally through the body wall muscle layer and run subepidermally towards the segment's dorsum (Fig. S15H). Each nerve innervates a number of epidermal sensory hairs. Interestingly, and in contrast with all other species described in this study except *Amphichaeta*, it is the third nerve the one that innervates hairs around the base of the chaetae (in contrast with nerve II in most other species). The fifth nerve is associated with the septum. The pattern of segmental nerves differs in the anterior region of the worm, with segment 1-2 showing 2 nerves and segments 3-4 showing 4 nerves (Fig. S16G).

Nais stolci

The nervous system of *Nais stolci* has three main components (Fig. S9): the anterior brain and associated peripheral nervous system, the ganglionated ventral nerve cord, and the segmental peripheral nerves.

Anterior nervous system

The brain is a paired bilobed structure located dorsal to the mouth; its anterior edge is slightly behind the boundary between prostomium and peristomium; its posterior edge is at the middle of segment 1 (Fig. S13K). It is composed of an outer cell cortex surrounding an inner neuropil (Fig. S12K), and is linked to the ventral nerve cord by paired circumesophageal connectives, which also connect to paired sets of prostomial peripheral nerves (Fig. S14K). The anterior part of the brain neuropil has a network of serotonin immunoreactive (SIR) neurites, while acetyl-tubulin immunoreactive (acTIR)

neurites form an arc across the posterior part (Fig. S12K). In the posterior lobe behind the neuropil there are four pairs of SIR perikarya. At the posterior edge of the brain is a set of acTIR hairs that are often associated with muscle fibers attached to that point and to the dorsal body wall. Two acTIR ciliary sense organs are located in front of the brain (Fig. S12K, Fig. S13K, Fig. S14K). The prostomium is cone-shaped (Fig. S13K); *Nais stolci* has a pair of lateral pigment-cup eyes located near the posterior edge of the prostomium (Fig. S9A). The acTIR tracts in the brain neuropil and the connectives arborize forward into a set of prostomial nerves that innervate a dense collection of epidermal acTIR sensory hair cells on the prostomial epidermis (Fig. S14K; see also diagram in Fig. 4).

Ventral nerve cord

The ventral nerve cord runs longitudinally down the length of the animal. There is one ganglion per segment, plus a subesophageal ganglion at the anterior end of the cord; the cell cortex is trough-shaped and a neuropil containing acTIR and SIR neurites runs through the trough. Along the ventral nerve cord, longitudinal acTIR neurites are found lateroventrally while longitudinal SIR neurites tend to be medial and dorsal; longitudinal nerve tracts are linked by segmentally iterated transverse commissures of variable acTIR neurite density (Fig S15I).

In all ventral nerve cord ganglia a number of SIR perikarya are found connected to the longitudinal SIR nerve tracts (Fig. S9D, Fig S15I). While the exact number and position varies both between and within individuals, we found usually 1-2 cells in the anterior third of the ganglion, 1 cell (weakly stained) located laterally to the right or left of the middle third of the ganglion, 1-2 cells near or lateral to the midline of the middle third of the ganglion, and 1 cell at the posterior third of the ganglion (see summary diagram in Fig. 5D). The distribution of these cells within a single ganglion is

asymmetric, but consecutive ganglia show mirror symmetry, mostly due to alternating left or right location of the lateral cells at the middle third of the ganglion.

The anterior portion of the ventral nerve cord shows substantial differences from more posterior segments. The subesophageal ganglion and ganglia from segments 1-4 show a pattern of SIR perikarya different from more posterior segments, with a larger number of cells that show bilaterally symmetric distribution (Fig. S16H). The subesophageal ganglion and ganglia from segments 1-3 have no obvious intervening connectives, thus forming an anterior medullary cord (Fig. S17F); ganglia from segments 4 and 5 are also continuous, while all remaining ganglia have clear connectives.

Starting at the boundary of segments 3 and 4, a muscular septum is found to separate the segmental coelomic compartments of the worm. In this species, each septum is located in front of the posterior third of the ganglion, so that the anterior two-thirds of the ganglion are located within the coelomic compartment of its corresponding segment, while the posterior third is located in the compartment of the following segment, a condition we denominate septate ganglia (Fig. 5B, 5D).

Peripheral segmental nerves

In each segment, four peripheral and segmentally iterated acTIR nerves branch off from the neuropil of the ventral nerve cord, exit the ganglia, pass ventrally through the body wall muscle layer and run subepidermally towards the segment's dorsum (Fig. S15I). Each nerve innervates a number of epidermal sensory hairs. Nerve II is the thickest, and innervates hairs around the base of the chaetae. Nerves I, III and IV are more or less similar in thickness, but nerve III tends to be shorter. Nerve III is associated with the septum. The pattern of segmental nerves differs in the anterior region of the worm, with segments 1-2 showing 2 nerves and segment 3-4 having 3 nerves (Fig. S16H).

A unique trait of the peripheral nervous system of *Nais stolci* is the segmentally iterated presence of a large SIR neuron on the lateral body wall (Fig. S9C, Fig. S18). This neuron is found close to the body wall's lateral line (see Fig. S11A for the location of the lateral line), level with the chaetae and slightly anterior to segmental nerve II.

Stylaria lacustris

The nervous system of *Stylaria lacustris* has three main components (Fig. S10): the anterior brain and associated peripheral nervous system, the ganglionated ventral nerve cord, and the segmental peripheral nerves.

Anterior nervous system

The brain is a paired bilobed structure located dorsal to the mouth; its anterior edge is slightly behind the boundary between prostomium and peristomium; its posterior edge is around the boundary between peristomium and segment 1 (Fig. S13L). It is composed of an outer cell cortex surrounding an inner neuropil (Fig. 2G, Fig. S12L), and is linked to the ventral nerve cord by paired circumesophageal connectives, which also connect to paired sets of prostomial peripheral nerves (Fig. S13L, Fig. S14L). The anterior part of the brain neuropil has a network of serotonin immunoreactive (SIR) neurites, while acetyl-tubulin immunoreactive (acTIR) neurites form an arc across the posterior part (Fig. 2G, Fig. S12L). In the posterior lobe behind the neuropil there are two pairs of SIR perikarya. At the posterior edge of the brain is a set of acTIR hairs that are often associated with muscle fibers attached to that point and to the dorsal body wall. Two acTIR ciliary sense organs are located in front of the brain (Fig. 2G, Fig. S12L, Fig. S13L, Fig. S14L). The prostomium is highly modified, forming paired horizontal lobes that flank a thin, long proboscis (Fig. 2G, Fig. S12L, Fig. S13L); *Stylaria lacustris* has a pair of lateral

pigment-cup eyes located near the posterior edge of the prostomium (Fig. S10A). The acTIR tracts in the brain neuropil and the connectives arborize forward into a set of prostomial nerves that innervate a dense collection of epidermal acTIR sensory hair cells on the prostomial epidermis (Fig. S14L; see also diagram in Fig. 4); one pair of nerves (denominated D in Fig. 4) extends the length of the proboscis.

Ventral nerve cord

The ventral nerve cord runs longitudinally down the length of the animal. There is one ganglion per segment, plus a subesophageal ganglion at the anterior end of the cord; the cell cortex is trough-shaped and a neuropil containing acTIR and SIR neurites runs through the trough. Along the ventral nerve cord, longitudinal acTIR neurites are found lateroventrally while longitudinal SIR neurites tend to be medial and dorsal; longitudinal nerve tracts are linked by segmentally iterated transverse commissures of variable acTIR neurite density (Fig S15J).

In all ventral nerve cord ganglia a number of SIR perikarya are found connected to the longitudinal SIR nerve tracts (Fig. S10D, Fig S15J). While the exact number and position varies both between and within individuals, we found usually 1-2 cells in the anterior third of the ganglion, 1 cell located laterally to the right or left of the middle third of the ganglion, 1-2 cells near or lateral to the midline of the middle third of the ganglion, and 1 cell at the posterior third of the ganglion (see summary diagram in Fig. 5D). The distribution of these cells within a single ganglion is asymmetric, but consecutive ganglia show mirror symmetry, mostly due to alternating left or right location of the lateral cells at the middle third of the ganglion.

The anterior portion of the ventral nerve cord shows substantial differences from more posterior segments. The subesophageal ganglion and ganglia from segments 1-4 show a pattern of SIR perikarya

different from more posterior segments, with a larger number of cells that show bilaterally symmetric distribution (Fig. S16I). The subesophageal ganglion and ganglia from segments 1-4 have no obvious intervening connectives, thus forming an anterior medullary cord; however, this trait is often obscured due to the tendency of *Stylaria* individuals to contract during fixation (Fig. S17G). We corroborated the location of the first connective by observing a number of additional individuals in a widefield epifluorescence microscope (data not shown).

Starting at the boundary of segments 3 and 4, a muscular septum is found to separate the segmental coelomic compartments of the worm. In this species, each septum is located behind the posterior third of the ganglion, so that the whole ganglion is located within the coelomic compartment, a condition we denominate non-septate ganglia (Fig. 5B, 5D).

Peripheral segmental nerves

In each segment, four peripheral and segmentally iterated acTIR nerves branch off from the neuropil of the ventral nerve cord, exit the ganglia, pass ventrally through the body wall muscle layer and run subepidermally towards the segment's dorsum (Fig. S15J). Each nerve innervates a number of epidermal sensory hairs. Nerve II is noticeably thicker, and innervates hairs around the base of the chaetae. Nerves I and IV are thinner, and nerve III is very short and sometimes absent or not detected. Nerve IV is associated with the septum. The pattern of segmental nerves differs in the anterior region of the worm, with segment 1 showing 2 nerves and segment 2 showing 3 nerves (Fig. S16I).
